# Elimination of a closed population of the yellow fever mosquito, *Aedes aegypti*, through releases of self-limiting male mosquitoes

**DOI:** 10.1371/journal.pntd.0010315

**Published:** 2022-05-16

**Authors:** Prabhakargouda B. Patil, Shaibal Kumar Dasgupta, Kevin Gorman, Angela Pickl-Herk, Mirel Puinean, Andrew McKemey, Bharat Char, Usha B. Zehr, Shirish R. Barwale

**Affiliations:** 1 GBIT Limited, Jalna, India; 2 Oxitec Limited, Abingdon, Oxfordshire, United Kingdom; International Atomic Energy Agency, AUSTRIA

## Abstract

Establishment of novel mosquito control technologies such as the use of genetically engineered insects typically involves phased testing to generate robust data-sets that support its safe and effective use as a vector control tool. In this study, we demonstrate the ability of the transgenic self-limiting OX513A *Aedes aegypti* strain to suppress a wild type *Ae*. *aegypti* population in an outdoor containment facility in India. OX513A is a genetically engineered *Ae*. *aegypti* strain with a repressible dominant self-limiting gene. When male adult OX513A mate with wild female adults, a single copy of the self-limiting gene is inherited by all the progeny, leading to death of >95% of progeny during larval/pupal development. A wild-type population of *Ae*. *aegypti* was established and stabilized during a 14 week period in five paired field cage units, each consisting of control and treatment cages, followed by weekly releases of OX513A male adults to suppress the target population. The successive introductions of OX513A male adults led to a consistent decline in wild type numbers eventually resulting in the elimination of *Ae*. *aegypti* from all treated cages within 10 to 15 weeks of release. This study demonstrates that *Ae*. *aegypti* elimination may be a realistic and achievable target in relatively isolated environments.

## Author summary

*Aedes aegypti* L. species is the primary vector responsible for transmission of the dengue virus worldwide including chikungunya, yellow fever and Zika virus. The experiment presented in the manuscript represents a study undertaken to demonstrate suppression of the wild type *Ae*. *aegypti* population in large outdoor field cages with natural exposure to the environment (physically-contained field cage facility) by sustained releases of male adults of OX513A *Ae*. *aegypti* strain. This investigation is a phase-2 contained study as per the World Health Organization guidelines for evaluation of genetically modified organisms and was recommended by the Indian regulatory board. This experiment demonstrates suppression of wild type *Ae*. *aegypti* population by sustained releases of OX513A male adults in a contained facility. The prospect of the project is to demonstrate and implement the technology for controlling/suppression of the *Ae*. *aegypti* vector in the open field environment.

## Introduction

Advancements in biotechnology have paved the way for novel techniques targeted towards safeguarding human and environmental health, such as the use of genetically modified organisms (GMOs), also termed as living modified organisms (LMOs) [1]. LMO’s could play a important role for providing global food security and could underpin the management of specific pest insects and vectors [2]. *Aedes aegypti* (Linnaeus), commonly known as the yellow fever mosquito, is a primary vector of several dangerous arboviruses including those that cause dengue, chikungunya and Zika in humans and animals [3–4]. In India, outbreaks of dengue and chikungunya have been increasing in frequency in recent decades with annual reported cases of 188,401 and 67,769, respectively, during the year 2017 (NVBDCP [5]). A recent study suggests that the actual number of dengue infections is higher than the number of cases reported [6].

The development of vaccines against dengue virus has shown promising results under trials, however, implementation has identified difficulties in mitigating risks, due to the complications caused by incomplete protection [7]. The presence of multiple dengue viral serotypes can increase the likelihood of severe forms of dengue viral infection (i.e. dengue hemorrhagic fever and dengue shock syndrome) if a previously infected person is subsequently infected with a different strain [8]. The World Health Organization has reported that controlling vector populations is the key factor to combat transmission of dengue [9].

Efforts to manage *Ae*. *aegypti* populations using current methods pose several challenges due to their oviposition behaviour and development in small water containers, anthropophilic nature, and multiple host biting behavior, which together result in an ability to transmit disease with a threshold level as low as 0.5 female *Ae*. *aegypti*/person, which corresponds to 0.25 pupae/person [10–11]. Current control methods include larval breeding site destruction, use of *Bacillus thuringiensis* and use of insecticides mostly involving application of non-specific broad-spectrum synthetic insecticides like pyrethroids, carbamates and DDT, in spite of their identified risks for resistance development and potential for undesirable effects on non-target organisms [12]. One promising alternative to conventional vector control strategies is the use of genetically modified mosquitoes [13], such as the OX513A *Ae*. *aegypti* strain, also known as the “Friendly Mosquito”. OX513A is a self-limiting strain with a tetracycline-repressible dominant self-limiting gene. Progeny fathered by OX513A male adult mosquitoes inherit a copy of the self-limiting gene, with >95% dying before reaching adulthood in the absence of the antidote, tetracycline [13]. This self-limiting approach means that there is no significant persistence of the transgene, and monitoring following field releases of OX513A has documented the complete disappearance of the transgene from the environment [14–17]. In addition, OX513A mosquitoes have been shown to be susceptible to currently used insecticides [18]. In 2016, the WHO Vector Control Advisory Group (VCAG) recommended the use of OX513A for pilot deployments in operational contexts in response to the Public Health of International Concern triggered by the Zika pandemic (http://www.who.int/neglected_diseases/news/mosquito_vector_control_response/en/).

The study reported here was a phase-2 evaluation of OX513A under natural exposure to the environment in physically-contained large field cages. The strain was tested for its mating competitiveness and its effectiveness in suppressing a local population of *Ae*. *aegypti*.

## Materials and methods

### Ethics statement

All experiments were performed in accordance with the approved experimental design and protocols, received from the Review Committee for Genetic Manipulation, the competent authority under the Department of Biotechnology, Government of India that monitors the safety related aspects of the projects involving genetically modified organisms (Letter No.BT/BS/17/328/2008-PID/Vol.2 dated; 15 Feb 2017). Representatives from five nearby villages, together with government representatives from State and District Health Departments, and the District Malaria Officer, were invited to the experimental site to disseminate information on the ongoing activities and raise awareness of this technology.

### Physically-contained field cage facility

The physically-contained field cage facility, hereafter referred as “Contained Facility”, was developed at the GBIT (Gangabishan Bhikulal Investment and Trading Limited) campus, located in a rural area near Dawalwadi village, Jalna District, Maharashtra State, India (S1 Fig).

The site was isolated from human habitation by more than 400 m and is 1 km west of Dawalwadi village (19°51’53.68"N, 75°47’30.80"E). There are typically three seasons during the year: the summer, between March and June, with limited rainfall and temperature between 35–45°C; the wet (monsoon) season between July and September with average rainfall between 650 to 750 mm, and the winter season, between October and February, with minimum temperatures between 9 to 10°C and maximum temperatures around 30 to 31°C. The contained facility was designed following the methods reported by Facchinelli et al. [19–20] with slight modifications (S2 Fig).

The contained facility consisted of six field cages fastened to a wooden platform of 580 m^2^ (29 m × 20 m) elevated 1 m above the ground on steel support poles. The footings of the supporting poles had ant traps containing detergent water. The ground below the platform was covered by a thick plastic sheet to prevent grass and other plants growing up to the platform. Six stitched cages of 8×8×3 m (l×w×h) size, constructed of white tricot knitted polyester woven UV (+40) treated mesh cloth (108 × 33 tpi) were supported over metal frames. Within each of these cages, experimental cages were erected by tying the nets to the metal frame supporting the outer cage with manila ropes at the sides, thereby creating a double layer of containment. The experimental inner cages were 6×6×2 m (l×w×h) stitched of white tricot mesh cloth (mesh count of 625 per square inch) partitioned into halves, providing paired cages with each half measuring 6×3×2 m (l×w×h), and the cages were reinforced at the angles and seams with white canvas. Each inner paired cage had vestibules at the opposite ends measuring 1.5 m×1.5 m (except one cage unit which was used as a field laboratory) with zippered opening to access into cages through the sleeves in the vestibules. Lockable zippered openings were installed for each cage on the opposite side of the vestibules for direct access into the internal space of cages when required. For all paired five cage units with vestibules, one half served as an untreated control and the other half as a treatment cage. The cage bases were fixed to the wooden platform by aluminum strips and screws to completely seal the cages. One cage without a vestibule was used as a field laboratory for handling of OX513A male adult mosquitoes, eggs, blood meal and other experimental materials. The outer cages served as double containment to ensure no escapees by providing a buffer zone of 1 m around the experimental inner cages, into which two BG Sentinel traps (Biogents) holding BG lure cartridge as attractant were placed to capture escaped mosquitoes, if any, the attractant were replaced at 16 to 20 weeks interval and electric mosquito bats provided to manually kill mosquitoes as required.

The height of the roof at the center of the platform was 6.5 m, made of translucent corrugated plastic sheets to provide shade and to protect the cages from rain and exposure to direct sunlight. White color floor mats were affixed on the whole platform. The whole physically-contained structure was protected by a chain link fencing on all sides upto to half of the height of the structure from platform having a lockable gate and a single entry point to protect from any unauthorized entry or animal intrusion. The upper half of all the sides of the structure were also covered with green shade net (50%) to provide additional UV protection to the cages.

Data loggers (HTC RH–Temp Data Logger Easy Log) were placed in each section of the cage and one placed outside the cage in the contained facility for recording temperature and humidity. One small potted plastic plant, a stack of clay bricks and clay pots were placed in each of the paired experimental cages as refuges for mosquitoes (S1 Fig). Each cage has a steel rack affixed inside the cage near the vestibule that could be accessed through the opening sleeves. A black plastic container with a clay pot containing water and covered by mesh was placed inside each cage on the rack as a refuge and to create a humid atmosphere for mosquitoes. Ovitraps were placed outside surrounding the whole unit for detection of potential escapees from the field cages. Rodent traps were placed at the bottom of the platform to ensure safety of the contained facility. Rodent traps used were lockable tamper-proof “Rodabox” baiting containing glue board (Trubble Gum) for trapping.

### Biosafety

Transport of all insect life stages during the experimental period between the laboratory and contained facility was within escape-proof, sealed containers/bags. All waste materials including mosquitoes were autoclaved before disposal.

### Mosquito strains

#### OX513A *Aedes aegypti* strain

Eggs of OX513A strain, transformed from *Ae*. *aegypti* Rockefeller strain, a parent strain, were imported from Oxitec, UK in accordance with the import permit (No.BT/BS/17/328/2008-PID) issued by the Department of Biotechnology (DBT), Government of India, New Delhi in November 2011 and the culture was maintained at an Arthropod Containment Level-2 (ACL-2) laboratory facility [21–23], Dawalwadi, Jalna District, Maharashtra State. The OX513A strain was tested to ensure homozygosity condition by screening for presence of fluorescence in heterozygous progeny of OX513A females mated with wild type males. As the OX513A strain possesses a dominant lethal gene and all the heterozygous progeny are expected to carry a copy of the lethal gene expressing fluorescence. In addition to fluorescence screening, genotype analysis was done to analyze the presence of transgene in homozygous OX513A progeny and heterozygous progeny through transgene specific primers and wild type allele specific primers.

#### *Aedes aegypti* wild type strain

Aquatic stages and eggs of *Ae*. *aegypti* were collected locally from Aurangabad during 2011 to establish the culture. Since then, the strain (hereafter referred as AWD) has been maintained under ACL-2 laboratory conditions.

#### Mosquito rearing

OX513A was maintained under ACL-2 laboratory conditions with a temperature of 27±2°C and relative humidity (RH) of 70–80%.

OX513A eggs were hatched under reduced atmospheric pressure for synchronized hatching and reared using tap water supplemented with 30 μg/ml tetracycline (chlortetracycline hydrochloride, 75%, Sigma), an antidote used for repressing the expression of the transgene. The larvae were provided with 2–3 drops of Liquifry (Interpet, UK) for the first day of development and later fed with ground “TetraMin Tropical Flakes” (Tetra Spectrum brands, Germany) during the rest of the developmental period until pupation, under a standardized feeding schedule. Male and female pupae were distinguished and separated based on the size difference and introduced into rearing cages of 30cm x 30cm x 30cm at 1:2 male/female ratio, prior to adult emergence. Introduction of male/female in the ratio 1:2 is followed, as male mosquitoes mate with multiple females without effecting the reproductive rate, and introducing adult females two fold that of males per cage increases egg productivity per cage [24]. The adults were subsequently provided with 10% sucrose solution and the female adults were fed with goat blood using a membrane feeding technique twice per week [24]. Eggs were collected once per week using water containers lined with seed germination paper–a substrate preferred by gravid females. Following collection, the papers were allowed to dry for 2 days before storing under laboratory conditions. AWD *Ae*. *aegypti* (wild-type) were reared as indicated above for OX513A, except for the addition of chlortetracycline to the rearing water.

### Experiment 1

#### Mating competitiveness experiment

Mating competitiveness experiments were performed to evaluate the performance of OX513A strain male adults versus male adults of AWD strain in the field cages.

Experiments were conducted as three replicates during the calendar month of March by introducing adult mosquitoes in a ratio of 100:100:100 (OX513A♂: AWD ♂; AWD ♀) into the cage. Experiments were initiated by first introducing virgin 3–4 day old male adult mosquitoes of both OX513A/AWD strains and allowing them to acclimatize for one hour. This was followed by the introduction of 100 female adult AWD mosquitoes. After 24 hours female adults were fed with goat blood. Adult females were collected post blood meal using a standard manual aspirator. When entering the cages, operators used protective clothing and full cover protective masks (Mutex Light+). The female adult collections were done simultaneously in the experimental cages to ensure similarity in the duration of collection of mosquitoes post mating period and the number of adults collected in each cage were recorded. Collected mosquitoes were transferred to ACL-2 insectary and introduced in rearing cages (30×30×30cm), and were fed with a second blood meal. Female mosquitoes were then transferred into individual mesh screened oviposition boxes [6.5 cm (Ø) and 8.0 cm (h)], each provided with a container of water [2.7 cm (Ø) and 3.0 cm (h)] lined with filter paper for oviposition. Eggs collected from each individual female were hatched and reared separately in tetracycline containing water, and screened during third instar larval stage for the presence of DsRed2 fluorescence (S3 Fig) using a fluorescence microscope (Leica MZ10F stereomicroscope with fluorescence filter set ET-DsRed composed with an excitation filter model ET545/30x peak wavelength at 545 nm with 30 nm bandwidth and emission filter peak ET620/60m at 620 nm with 60 nm bandwidth). Fluorescent progeny indicated that the offspring were fathered by OX513A males, and non-fluorescent progeny indicated offspring fathered by AWD wild type males. Presence of both fluorescent and non-fluorescent larvae indicated double mating by female adults with both AWD wild type and OX513A male adults.

#### Pupal size

The size of male pupae from the experimental rearing lot was determined by measurement of the dorsum of the thorax (S4 Fig). Pupae were transferred to a small petri dish holding cold water to immobilize the pupae and a measuring scale along the side. Images were captured manually using a 14 megapixel camera and the dorsal thoracic region measured using “ImageJ” software to determine the pupal size.

#### Wing measurement

Right wing length was used as an indicator of adult size. Adult mosquitoes emerging from the experimental rearing lot were preserved in 90% alcohol for dissecting the wing for measurements. Wings were removed under a dissection microscope and mounted on a slide in lactophenol and sealed with transparent nail polish. The images of the mounted wings were taken alongside a measurement scale. The images were analyzed in Image-J software and measurements of the wing taken from the alular notch to the apex margin of the wing, excluding the fringe (S5 Fig).

#### Population suppression experiment

The population suppression experiment consisted of two stages; (1) Establishment of a stable population of wild type AWD strain and (2) Attempted suppression of wild type AWD population by frequent releases of OX513A male adults in treatment cages.

#### Population stabilization

The method for stabilization of the AWD population was adapted from that reported by Wise de Valdez et al. [25]. Initially all 10 cages were populated by the introduction of 200 male and 200 female adults (AWD strain) and provided with 10% sucrose. Female mosquitoes were fed with goat blood twice a week using feeding membranes. Following the blood feed, black plastic containers containing water and lined with a paper strip were added to collect eggs. Eggs laid by females were collected weekly, dried, counted and hatched. The hatched larvae were reared and returned to their respective cages as second-instar larvae (L2) at a return rate of 200/week. Only during the initial period of population establishment and to ensure the same number of larval introduction into all cages, the number of larvae introduced (i.e. 200 larvae) was supplemented, if required, with larvae from the laboratory AWD colony. Weekly egg counts were used to determine establishment of stable populations.

To monitor the population dynamics, adults were sampled weekly using BG Sentinel traps (Biogents, Germany) placed in each cage for sixty minutes and the adult males and females captured were counted and returned to their respective cage. Mortality, if any, during adult monitoring was noted. Weekly monitoring of the population using BG sentinel traps was conducted throughout the experimental period (i.e. population stabilization and population suppression period).

#### Population suppression

In all the control cages, the weekly replenishment rate of 200 L2 larvae from the eggs collected weekly in the respective cages was held constant throughout the experimental period.

In treatment cages following the population stabilization, in addition to the weekly introduction of 200 L2 larvae, OX513A adult male introduction was initiated. The released OX513A male adults were reared under laboratory conditions and were sex-sorted during the pupal stage. The sorted male pupae (100/container) were added to plastic containers containing water and covered with a mesh, and allowed to emerge. Emerged adults were visually inspected and female adults (if any) were removed using an aspirator. Male cohorts for release were transferred to the contained facility for releasing at a ratio of 10:1 (OX513A males: AWD males) in each treatment cage (i.e. 1,000 male adults/treatment cage/week), approximately 10 times the weekly return rate of 200 second instar AWD larvae/week, with a male/female emergence ratio of 1:1 (approximately 100 AWD males). The release number of OX513A male adults was constant throughout the experimental duration.

After initiating the releases of OX513A male adults, L2 larvae equivalent to 10% (if egg count was >2,000) of the egg yield or 200 L2 larvae (if egg count is <2,000) and or all the larvae hatched (if egg count was <200) in each cage were screened for the presence of DsRed fluorescence. After fluorescent larvae were observed, thereby confirming mating with OX513A strain male adults, the number of larvae replenished back to respective treatment cage was proportionally adjusted to reflect the egg yield and larval introduction rate (i.e. 200 larvae) of respective paired control cage [25]. For example, if a control cage produces 1,000 eggs one week with constant return rate of 200 larvae per week (1/5), and a treatment cage produced 2,000 eggs in the same week than a similar proportion of the total number of eggs (1/5) is returned as L2 larvae to the cage (i.e. 400 larvae). If a treated cage in the same week produces 500 eggs then 100 larvae will be returned to the cage.

As discussed in previously published literature [25], if 200 larvae are returned constantly to each cage in both control and treated cages, the return rate will increase the population in the treated cages if the egg density is low, or decrease it if the egg density is high.

#### Wing measurement

During weeks 1–3 following release initiation, 25 emerged adults from each OX513A male adult release cohort and 25 male adults from each control cage were collected to determine and compare the body size based on wing measurement.

### Statistical analysis

#### Mating competitiveness

The t-test was used to compare the numbers of females mated with OX513A, AWD males and double-mated females (both with OX513A and AWD strain male adults). Chi-square tests were applied to test the observed mating index against the expected mating index. Relative mating index was calculated as the number of females mating OX513A males/total number of females mated [26].

#### Population suppression experiment

Statistical comparisons of two independent groups, such as wing size between OX513A and AWD, egg production and egg hatch between treatment and control cages, were performed using the independent t-test. The distributions of weekly eggs produced in cages were assessed for normality using the Kolmogorov-Smirnov (K-S) test. Weekly variation of egg production in treatment and control cages were analyzed by one-way analysis of variance (ANOVA) followed by post-hoc Tukey’s B test.

To compare the egg production between paired treated and control cages during the suppression phase, the data was normalized first by converting it to square root and analyzed using one-way analysis of covariance (ANCOVA). Any effect of OX513A releases in treatment cages was detected by comparing weekly egg production with the respective paired control cage.

The weekly change in the numbers of male and female adults captured by the BG Sentinel traps in the paired control and treated cages was analyzed using the non-parametric Mann-Whitney U test. The aim was to determine whether the number of males and females differ significantly between the paired control and treatment cages before and after initiating weekly introduction of OX513A males. In addition, for the graphical representation of the adult variation in each cage, the values were log-transformed.

To determine whether the temperature and humidity influenced the mosquitoes’ egg production, a correlation analysis (Pearson correlation coefficient) of the weekly average temperature/humidity (maximum and minimum) and egg production in control and treatment cages before and after OX513A releases was carried out. Adverse humidity and temperature impact egg production significantly and could mislead the suppression trials results. The weekly maximum and minimum averages for temperature and humidity were calculated from the daily maximum and minimum temperatures recorded between Wednesday to Tuesday in each respective cage (the day range was determined on the ovitrap retrieval day—Tuesday). At the end of the experiment the sucrose feeders were removed for a period of 10 days to ensure all mosquitoes were dead, the cages were cleaned and the biological waste was autoclaved for disposal.

Pearson correlation coefficients were also calculated for the relation between the number of fluorescent larvae and the egg production in each of the treatment cages as well as between the number of fluorescent larvae and the larval return, to indicate whether and how the variables tested influenced each other. Statistical analyses of the data were conducted using statistical software SPSS version 20 (IBM Corporation, New York, USA).

## Results

### Mating competitiveness

Assessment of the mating competitiveness provides data pertinent to the effectiveness of the strain for population suppression strategy. Mating experiments between male adults of the AWD and OX513A strains in equal proportion were conducted for a period of 24 hours.

Measurements of the pupal size and right wing of adults indicated no significant difference between the two strains, with a mean pupal size of 0.981±0.01 mm and 0.977±0.012 mm (p = 0.789, two tailed independent T test) and mean right wing length of 2.04±0.01 mm and 2.02±0.02 mm (p = 0.447, two tailed independent T test) for OX513A and AWD males respectively (S1 and S2 Tables).

**Fig 1:**
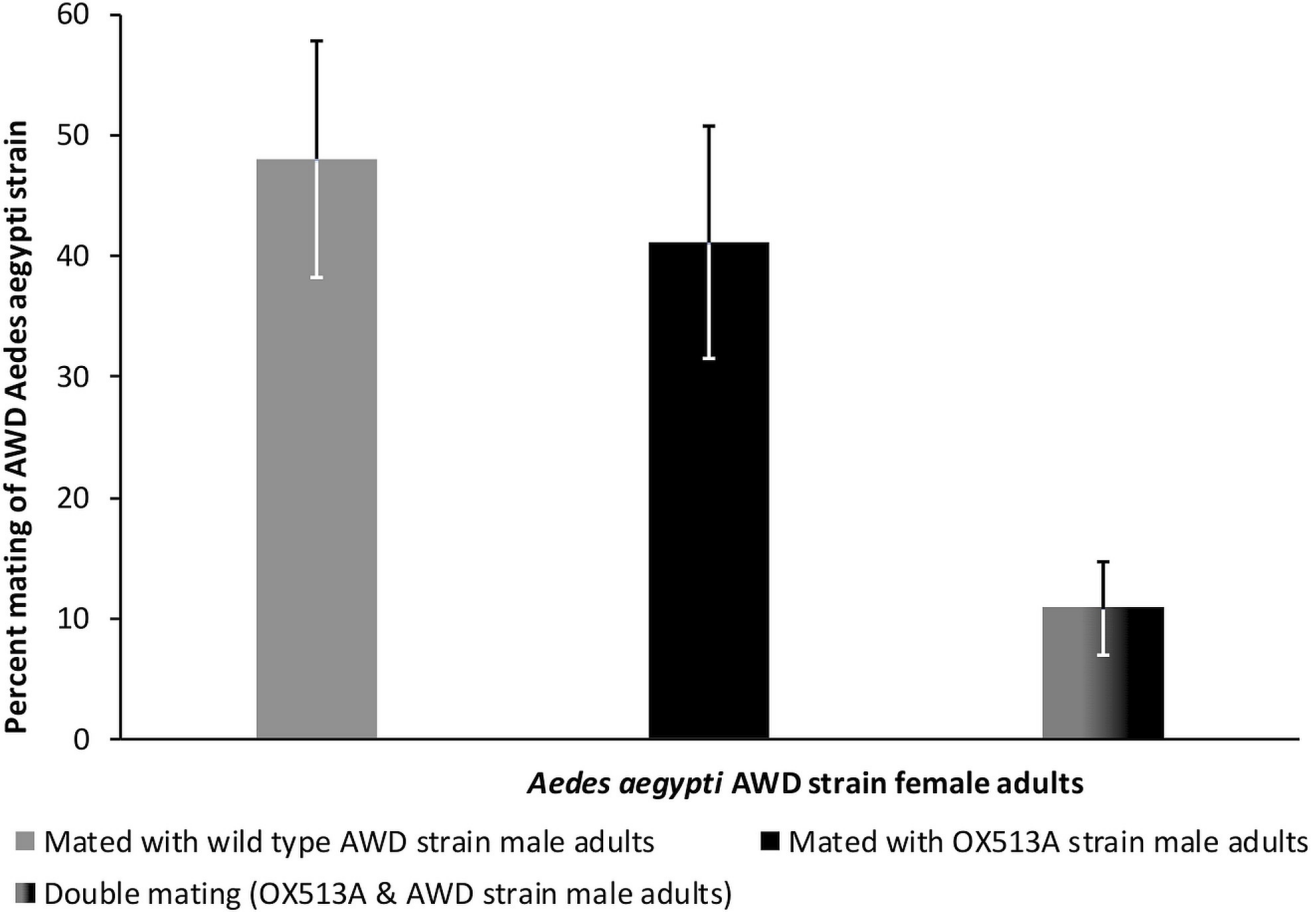
Percent mating of *Ae. aegypti* female adults with male adults of OX513A/wild type AWD *Ae. aegypti* strain and error bar representing standard error.

Twenty-four hours after the mating release period, out of 79.7% of the female adults recaptured, 60.3% females laid eggs with an average of 58 eggs per female adult (S3 Table). Eggs laid by individual females showed a hatching rate of 76.6%. Fluorescence screening revealed that 41% and 48% of the AWD female adults mated only with OX513A males and AWD males respectively (F value = 0.681; p = 0.691), while 10.9% of the AWD adult females mated with both OX513A and AWD males (Fig 1).

Chi-square analysis (S4 Table) of the observed mating proportions revealed non-significant deviation from the expected mating proportion of 1:1:0 (AWD males: OX513A males: double mating) (X^2^ = 2.886; p = 0.2362). In addition, the paternal origin (OX513A male / AWD male) did not impact on the number of eggs laid by the wild females (Table 1).

**Table 1:** Average number of eggs laid by AWD females mated with OX513A males, AWD males and those double-mated.

	AWD female adults mated by		
	AWD strain ♂	OX513A Strain ♂	Both [AWD & OX513A strain] ♂
Number of eggs laid/female (Mean±SE)	58.5±1.97^a^ (n = 4914)	60.6±1.85^a^ (n = 4360)	59.7±3.97^a^ (n = 1134)
95% confidence interval	54.6–62.4	56.9–64.2	51.3–68.0
One-way ANOVA	df = 2; F = 0.284; p>0.05		

Figures within the rows indicated with the same letter (i.e. "a") show non-significant differences between the values by one-way ANOVA following Tukey’s-b test.

### Population suppression experiment

**Fig 2:**
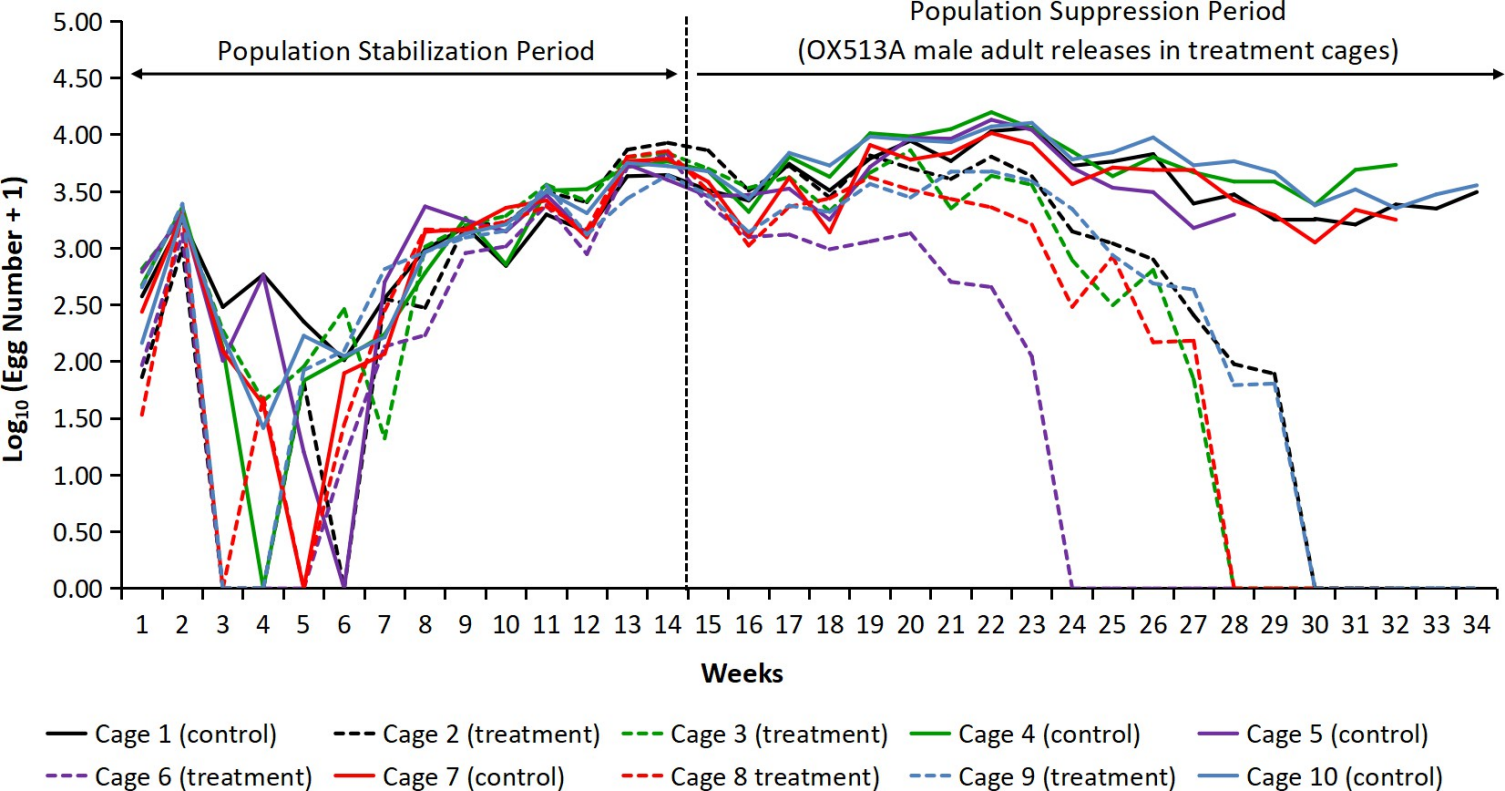
Weekly egg production in control and treatment cages during experiment. Egg production number increased from week 9 reflecting establishment and stable adult mosquito population during subsequent weeks in all cages. Post establishment, OX513A male adult releases weekly were initiated (weekly 15 onwards) (vertical dashed line) in the all 5 treatment cages.

The suppression experiment was initiated on 13^th^ April 2017 and terminated on 12^th^ December 2017 after 34 weeks. Experiments were performed in five paired cage units (Unit A to E) with a control and treatment cage in each unit. Each paired unit had the control and treatment cages randomly assigned—cages 1, 4, 5, 7 and 10 were controls while 2, 3, 6, 8 and 9 were treatment cages. Establishment of a stable population of wild AWD strain–defined as stable egg output, was achieved in 14 weeks in all the paired cage units (Fig 2). Number of eggs produced in each cage was analyzed by one-way ANOVA, which indicated a significant increase of the mosquito population from week 8 while egg production was stabilized by week 14 (S5 Table).

The K-S test for normality of the distribution on weekly egg production showed non-significant difference between cages indicating a normal egg production rate during the population stabilization period from week 1–14 (p >0.05). One exception was week 4 where the egg production varied significantly (p<0.05) between the cages (S5 Table). Analysis of the weekly mean egg production during the stabilization period showed no significant differences between paired control and treatment cages (p>0.05). Egg production during week 13 and 14 increased significantly in control and treatment cages compared to the previous weeks but mean egg production for weeks 13 and 14 was not significantly different between the control and treatment cages indicating stabilization of the AWD population (S5 Table).

Across the whole stabilization period (14 weeks) the weekly egg production in paired cages did not vary significantly (p>0.05) indicating no bias of the cage position on egg production (S6 Fig). Stabilization of the AWD population in cages was followed by weekly introduction (from week 15) of 1,000 OX513A male adults in each treatment cage, corresponding to a release of 10 fold of AWD male adult emergence in the control cages (ratio of 10:1 OX513A males/AWD males), based on the weekly introductory rate of 200 L2 larvae per cage and considering our earlier laboratory observations on the sex ratio of adult emerging from the eggs laid by females (i.e. 1:1 male/female ratio). OX513A male releases in treatment cages were terminated when no egg production was observed consecutively for a period of 5weeks.

**Fig 3:**
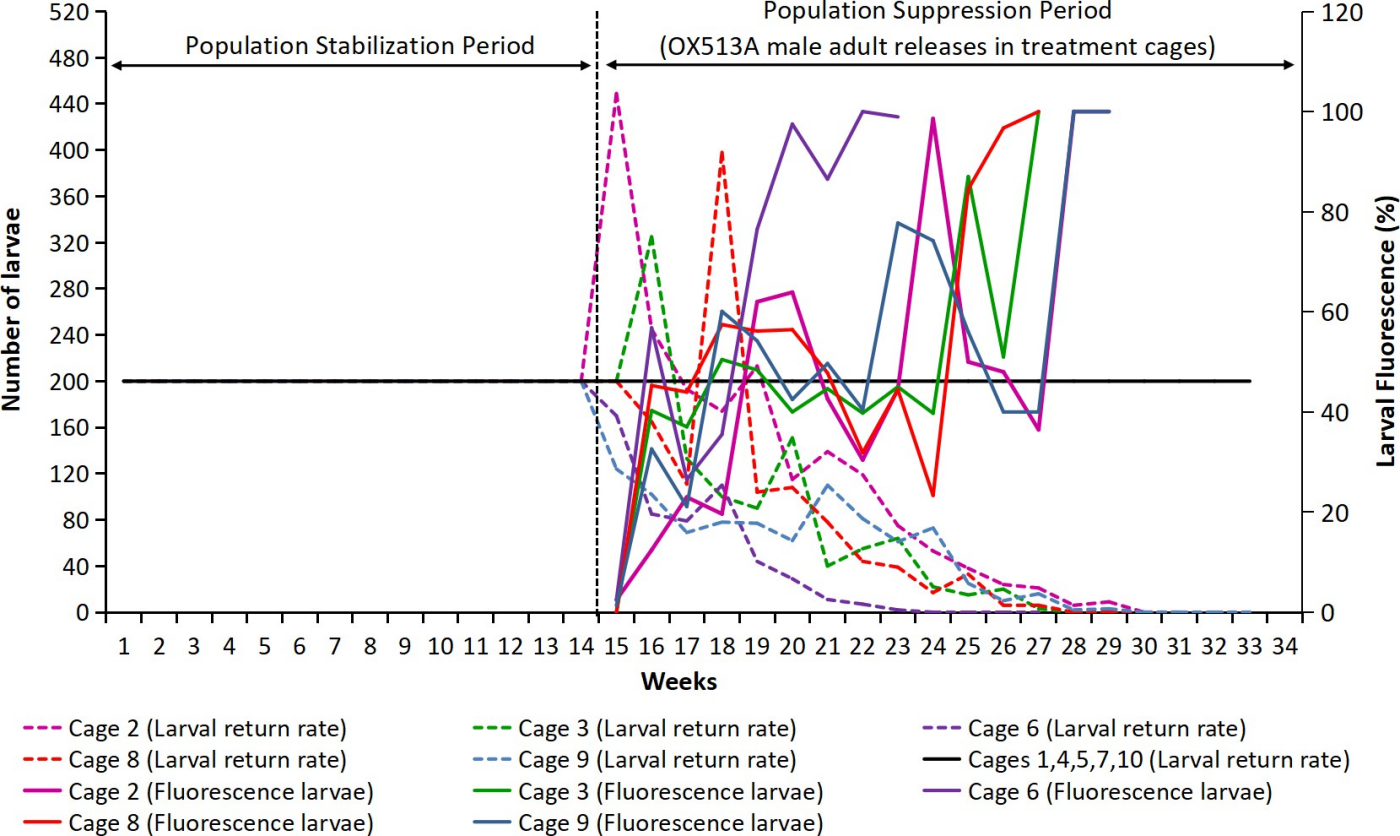
Weekly larval return rate in control and treatment cages before and after OX513A male adult releases and fluorescence observation. Post OX513A male adult release initiation in treatment cages and following first observation of introgression of OX513A based on fluorescence screening of progeny, the larval return rate was calculated for the treatment cage in proportion to paired control cage.

Following the weekly introduction of OX513A male adults in treatment cages, the eggs collected weekly were hatched and screened for presence of DsRed2 fluorescence. Calculations of weekly larval return rate were initiated for the treatment cages following the identification of the first fluorescent larvae (Fig 3).

Weekly larval return rate in treatment cages for cage 2 and 3 was high during the first two weeks (i.e. week 15 and 16) post OX513A release initiation, corresponding to the egg production rate and gradually decreased in the subsequent weeks. In cage 6, 8 and 9 the larval return rate decreased proportionately with the decrease in egg production followed by the increase in percent fluorescent (Figs 3 and S7). Mortality during the development stages following weekly introduction of larvae (L2) into the cages was analyzed from week 18 onwards. Mortality in the treatment cages observed during the week 18 to week 24 fluctuated between 46.8% and 59.4% and reached 100% by week 28 and 29 (S8 Fig).

Analysis of egg hatching rate in control and treatment cages during pre- and post-release period showed non-significant differences (p>0.05) (S9 Fig).

Correlation analysis of larval fluorescence against egg production (Table 2) was found to be significant and negatively correlated in treatment cage 2, 3 and 6 and non-significant in cages 8 and 9 (p<0.05). The correlation analysis between the larval fluorescence and larval return rate found significant and negative correlation in cages 2, 3, 6 and 9 (p<0.05).

**Table 2:** Correlation between fluorescence with egg production and fluorescence with larval return rate post initiation of OX513A male adult introduction in treatment cages from week 15.

Correlation	Treatment cages				
	Cage 2	Cage 3	Cage 6	Cage 8	Cage 9
Fluorescence with Egg production					
Pearson Correlation (r value)	*-0.543	*-0.646	*-0.769	-0.455	-0.311
Sig. (2-tailed)	0.036	0.017	0.016	0.118	0.26
Fluorescence with Larval return rate					
Pearson Correlation (r value)	**-0.685	*-0.554	**-0.947	-0.298	*-0.583
Sig. (2-tailed)	0.005	0.05	0.0001	0.323	0.023

*Correlation (negative) is significant at 0.05 (2-tailed).

**Correlation (negative) is significant at 0.01 (2-tailed).

**Fig 4:**
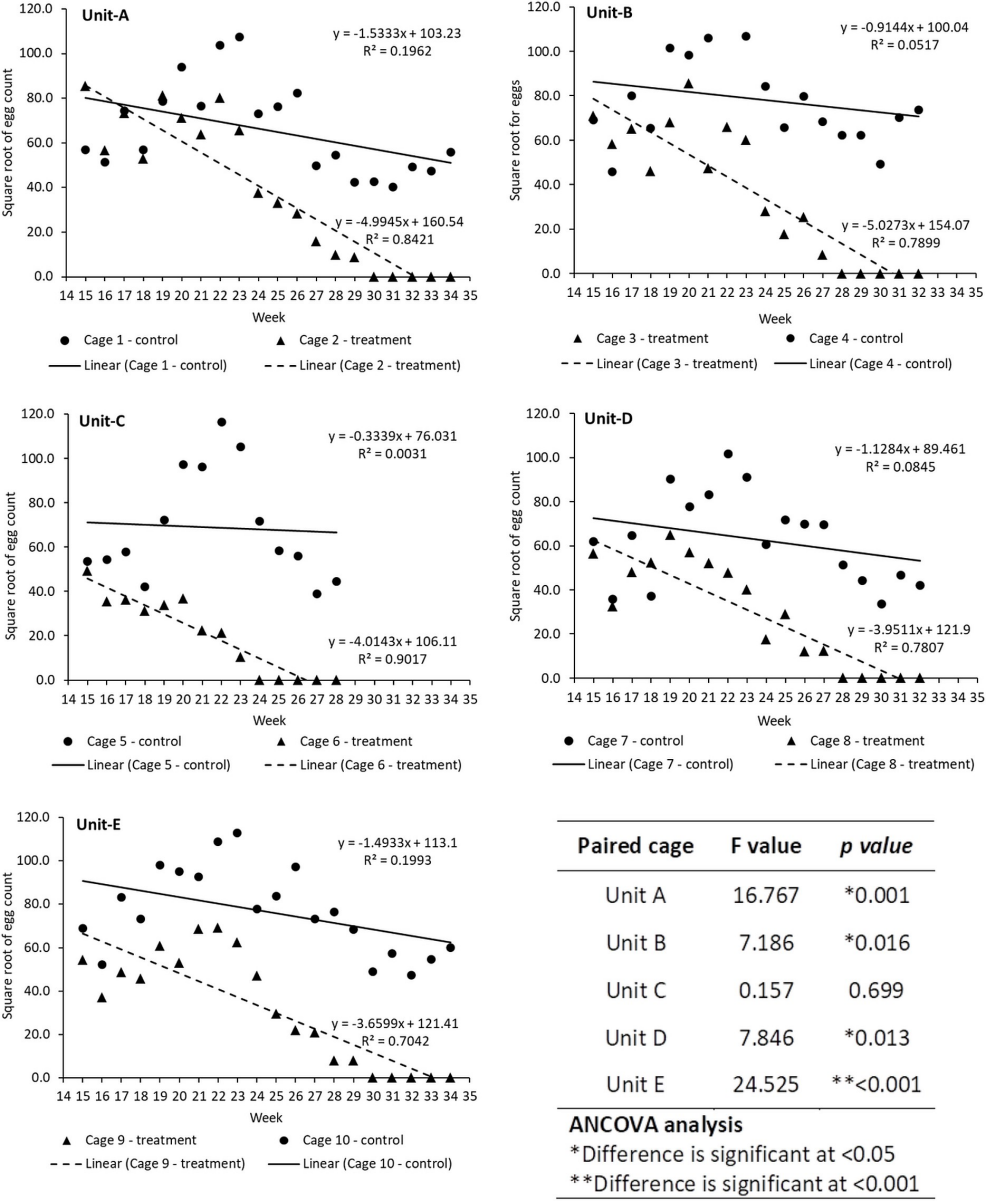
Egg production of *Ae. aegypti* AWD strain mosquito in paired cage (control versus treatment) units (A-E) during suppression period (OX513A male adult releases in treatment cages) from week 15 and ANCOVA analysis.

Following the initiation of OX513A male releases in treatment cages, average egg production decreased significantly (p = 0.005) from week 20 (Fig 2 and S6 Table), while for cage 6 the decrease in egg production was visible from week 17. Egg production analysis by ANCOVA in paired cage units A, B, D and E was significant (p<0.05) except cage unit C, indicating significant reduction in egg production in respective treatment cages post initiation of OX513A male adult releases (Fig 4). In cage 6 egg production significantly decreased and no egg production was observed post 9 weeks of OX513A releases i.e., from week 24 and no further egg production was observed subsequent for 4 weeks till the week 28 from the start of the experiment.

The AWD population was considered eliminated when no egg production was recorded for a period of 5 consecutive weeks in the treated cages. The first treatment cage with no egg production was Cage 6 in week 24, followed by Cages 3 and 8 by week 28, and Cages 2 and 9 by week 30. In all control cages, the AWD egg production continued until they too were terminated after 5 weeks of no egg production in the paired treated cage (i.e. the control cages were terminated 5 weeks after the production ceased in the paired treated cage) (Fig 2).

**Fig 5:**
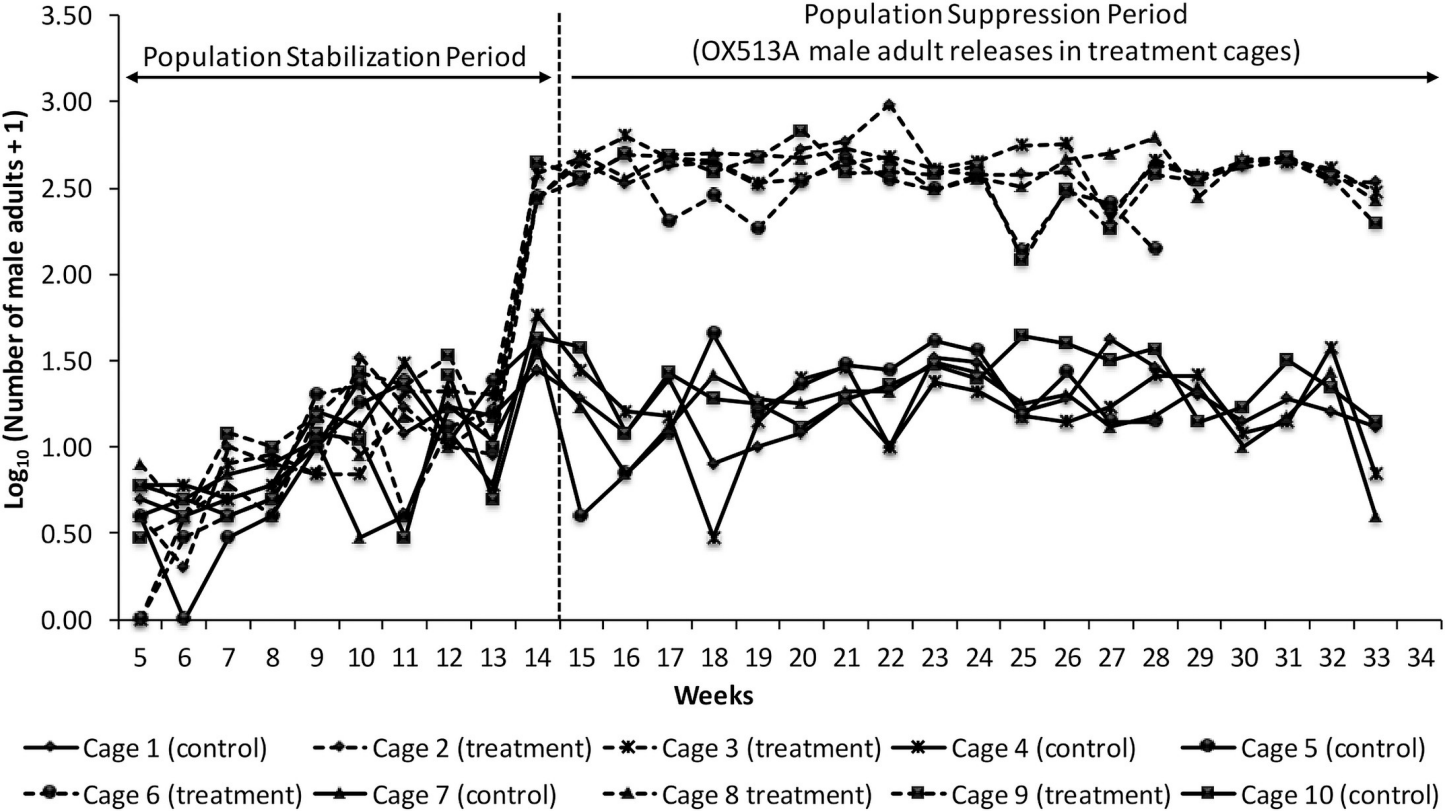
Male adult sampling using BG sentinel traps in control and treatment cages (weeks 5 to 33).

**Fig 6:**
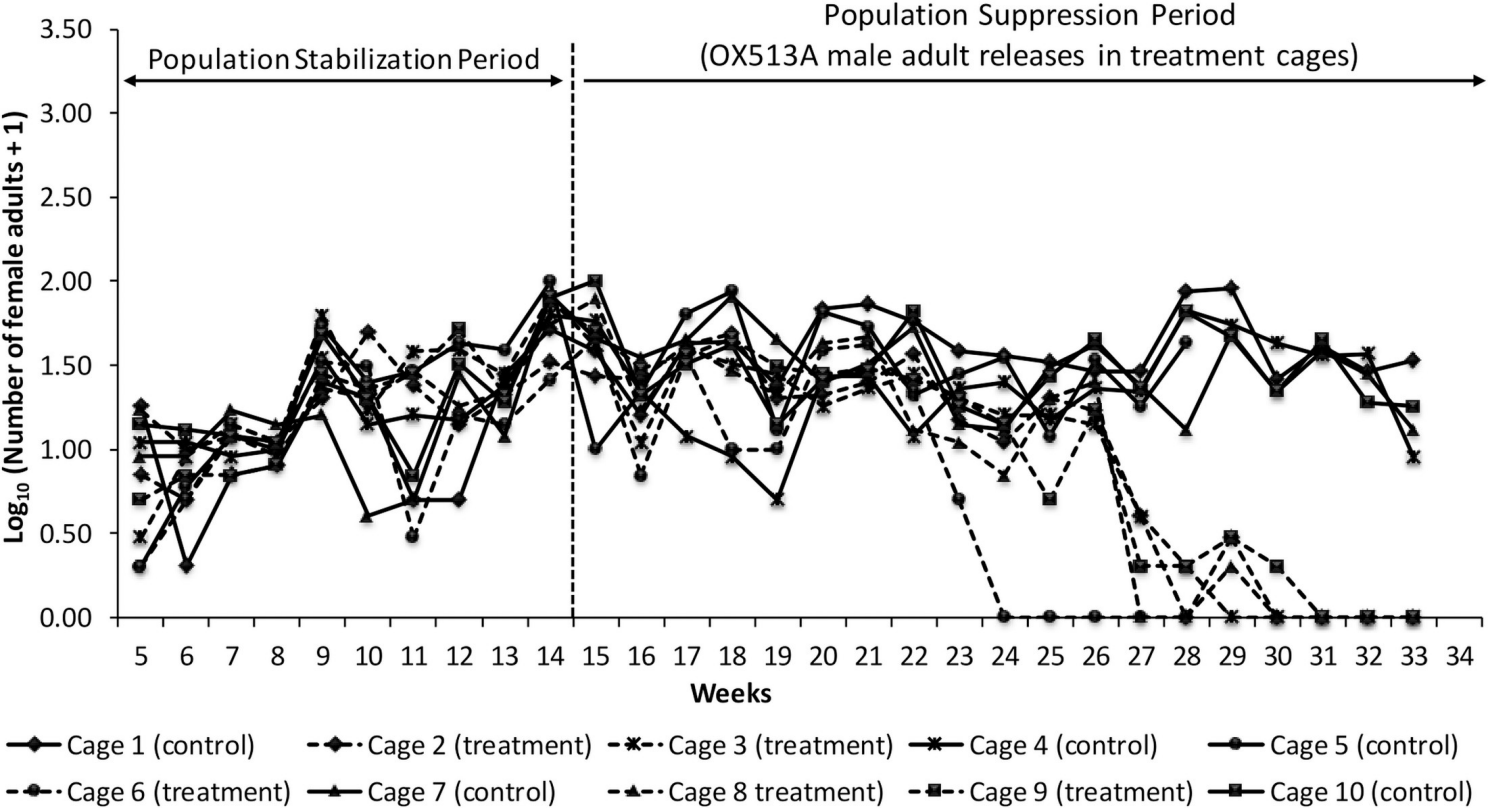
Female adult sampling using BG sentinel traps in control and treatment cages (week 5 to 33).

Adult sampling using BG Sentinel traps was performed weekly in all the cages from week 5 to determine the variation in male and female numbers. Following the initiation of OX513A releases (week 15), the number of adult males captured in the treatment cages significantly increased compared to control cages (Fig 5). The number of adult females captured in the treated cages decreased significantly compared to the control paired cages starting from week 23 (cage 6) and week 27 (cages 2, 3, 8 and 9). No female mosquitoes were captured in treated cages from weeks 30 (cage 2), 29 (cage 3), 24 (cage 6), 30 (cage 8) and 31 (cage 9) (Fig 6).

Statistical analysis of total males and females captured in paired cages has indicated that before releases the number of males and females captured was not significantly different between the paired treated and control cages (S7 Table). However, during the release period, both the total number of males and females captured was significantly (p<0.05) different between the paired control and treated cages (except for Unit B which narrowly missed the significance threshold–p = 0.06) (S8 Table).

To determine whether the environmental factors had an impact on the mosquito egg production, the temperature and humidity were recorded hourly in all the cages throughout the experimental period, using data loggers. The conditions in the treated and control cages were found to be similar for both temperature and humidity (S10 Fig). The correlation analysis between the egg production and the temperature and humidity variation revealed a number of statistically significant positive and negative correlations, however, none that would provide support to a decrease in egg production in the treated cages due to environmental conditions (S9 Table).

## Discussion

Our earlier laboratory assessments of OX513A strain for mating competitiveness and self- limiting gene expression have shown the OX513A strain to be effective and equally fit in comparison with wild type *Ae*. *aegypti* mosquitoes [27]. Testing under confined conditions that limit the release of the organisms into the environment forms part of the phased testing approach recommended by some authorities to assess the safety and efficacy of novel technologies [28]. A lack of fitness could potentially undermine performance by reducing the competitiveness with wild type males under open field conditions. Consequently, for developmental and deployment of such biological approaches it is useful to define and test fitness, to help predict the likelihood of success of an intervention strategy [29]. In contrast, another topic that arose is the fitness of the AWD wild type strain used to populate the cages for demonstrating population suppression. Our studies on mitochondrial cytochrome oxidase I (COI) region of laboratory strains including AWD strain and field collected wild type strains have shown genetic similarity with field collected wild type strains and Indian/worldwide isolates [30]. Our earlier laboratory studies on fitness parameters support the AWD strain to be an ideal wild type strain for cage studies [27]. In the present study, we demonstrate the OX513A strain of *Ae*. *aegypti* is equally competitive for mating counter to wild type males and can cause elimination of target wild type population by sustained release of OX513A release within a time frame in field cages.

For the mating competitiveness studies, the sizes of OX513A males and AWD males under test conditions were analyzed by comparing the size of the pupae during development and wing length for emerging adult size. Some reports suggest that size of the mosquitoes is associated with fitness and reproductivity, and that larger mosquitoes could be at an advantage over smaller ones [31–33]. Measurements of OX513A and AWD pupae and adults for comparisons of size indicated no significant difference between the two strains. Results of the mating experiments indicated OX513A male adults to be equally competitive in comparison with the wild AWD strain, with 41.1% of the females being mated by OX513A males compared to 48.0% mated by AWD males. Although a small percent of females (10.9%) were double-mated, the chi-squared test showed that the deviation from relative mating proportion (observed versus expected—1:1:0, OX513A mated: AWD mated: double mated) was non-significant. The calculated expected proportion for double mating was considered to be zero based on the fact that the *Ae*. *aegypti* are monoandrous and typically mate once in their life span, and are commonly refractory towards a second mating over time post first mating [34–35]. This refractory/unreceptive behavior towards mating is induced due to the secretion of a protein/factor called “matrone” by male mosquitoes during insemination into the female adult mosquitoes [36]. In an earlier laboratory study, Massonnet-Bruneel et al. (2013) [37] found that the OX513A males are as efficient as wild-type males at inducing refractoriness to re-mating in wild-type females. Statistical analysis of the double mating found in the present study indicated that the observed proportion of the female mating with both AWD and OX513A males was non-significant by analysis of deviation from expected proportion of double mating as zero. In this context, it is important to emphasize that double mating observed in the cage study may be attributable to the restriction of movement for mated female adults in the cage which could have led to frequent encounters with the males of both the strains leading to second mating.

Short-term laboratory competition studies have been shown to underestimate the fitness costs of the strains analyzed and that a better way to assess the mating competitiveness before field release is through large caged experiments under field conditions [20]. Our earlier laboratory studies on the mating competitiveness have shown the OX513A strain male adults to be equally competitive against wild type males [18]. The results of the mating studies under field cages further support that the transgenic OX513A males have similar mating competitiveness, and the AWD females do not preferentially discriminate between the two strains indicating the strain to be competitive.

To demonstrate population suppression, stabilization of AWD population was achieved in a 14 weeks period with an average of >5,000 eggs production per cage. The stabilization point for the mosquito population (weekly egg output) in the cages was determined following the reproductive data from the mating competitiveness experiment and weekly larval return regimen. An average of >5,000 eggs at week 14, at the end of July 2017, with the start of rainy season and favorable climatic conditions (temperature/humidity) for mosquito breeding, was considered ideal for initiation of OX513A male releases in the treatment cages. Previous large cage studies using *Ae. aegypti* populations have also used pre-release periods of up to 20 weeks with the mosquito population showing stability in egg output after 10 weeks of maintenance [20,38–39].

Previous studies on open field releases [15] have used a 10:1 ratio (OX513A males: AWD males) that was shown to favor population extinction. Here we used a similar over-flooding ratio with a weekly release of 1,000 OX513A adult males and the return of 200 larvae, thus the release ratio increased as the experiment proceeded, due to the fact that the release ratio was maintained constant. Following the detection of fluorescent larvae and the decrease in overall cage reproductive output, the number of larvae returned represented 10% of the total number of eggs produced in a week. This strategy was previously used in other studies [25] to avoid the artificial increase or decrease of a population by using a constant larval return. OX513A male adults in the treated cages increased significantly during the OX513A release period, while the number of females started to decrease after week 20. These results were consistent with the progression of the fluorescent proportion in the population. In effect, the negative correlation identified between the fluorescence level and egg production supports the observation that the release of OX513A males has led to a gradual reduction of AWD female numbers. The reduction in the number of AWD females impacted on the production of eggs, which followed a similar decreasing pattern after the initiation of OX513A releases: by week 19 the number of eggs has decreased significantly in the treated cages while by week 22 the difference was significant.

Our experimental findings clearly demonstrate that OX513A strain is competitive under natural environmental conditions in India, indicating its potential to produce the desired effect under open field environment and suppress natural populations of *Ae. aegypti* in India effectively. OX513A has previously been shown to be effective in suppressing wild populations of *Ae. aegypti* in Brazil, the Cayman Islands and Panama [14–17,40–41].

All the experiments were conducted with the approval of the regulatory authority and disseminated the information on the experiments to state government health officials and representatives of nearby villages for community engagement. This approach helped us to gain confidence of the local community, support and build the trust that the transgenic strain is being tested step by step with all the precautions, which would help us to gain support of the local residents for the next stage open release trials of the strain.

Notably, with the advancement of the technology and wealth of knowledge gained over more than a decade of deploying the first generation technology (OX513A), there is a transition from first generation technology to an advanced second generation technology, OX5034 strain [42]. It is important to emphasize here that the new generation technology retains characteristics of its first generation, which has passed stringent safety evaluation in many countries worldwide. Although the second generation strain has been introduced, the experience gained with the OX513A strain from the laboratory studies, field cage studies, and community engagement would help and support to evaluate and deploy either of the generation strains with approval from regulatory bodies.

## Conclusion

The present study demonstrated that OX513A strain has a similar mating competitiveness with the mosquito population of local strain and that it is an effective tool for the control of the wild population of *Ae. aegypti* by completely suppressing the wild populations in the 5 replicated cages within 10 to 16 weeks of releases. The findings from the experiments are encouraging and support to proceed demonstrating suppression of wild type target populations under open field conditions by sustained releases of OX513A males in the future in India. For conducting open field release trials, it is important to emphasize here that transparency and public involvement opportunities are essential for gaining the confidence of the local communities and a sustainable implementation of the technology in the future.

## Supporting information

S1 Fig (a) physically-contained field cage facility, (b) field cages, (c) arrows indicate larval container covered with mesh and sucrose feeding rods, (d) arrows indicate refuges for mosquitoes, (e) ant traps surrounding the supporting pillars, and rodent traps, (f) arrows indicate sucrose feeding cotton pads, and a black plastic container, holding an earthen pot containing water and covered with mesh cloth as a refuge. (TIF)

S2 Fig Physically-contained field cage facility design and specifications. (TIF)

S3 Fig Fluorescence imaging of OX513A *Ae. aegypti* strain larvae in comparison with AWD strain *Ae. aegypti*. OX513A larvae display bright punctate fluorescence throughout the larva, which is distinct from auto fluorescence from the larval gut that is also observed in the AWD larvae.
(TIF)

S4 Fig Measurement of *Ae*. *aegypti* pupal width on the dorsal side of cephalothoracic region. (TIF)

S5 Fig Right wing measurement of *Ae*. *aegypti* male adults of OX513A and AWD strains. (TIF)

S6 Fig Average number of eggs produced in paired cage units per week in control and treatment cages during stabilization period (week 1–14) with error bar representing standard error. (TIF)

S7 Fig Average of weekly larval return rate in control and treatment cages before and after OX513A male adult releases and fluorescence observation. Post OX513A male adult release initiation in treatment cages and following first observation of introgression of OX513A based on fluorescence screening of progeny, the larval return rate was calculated for the treatment cage in proportion to paired control cage. Error bar represents standard error.
(TIF)

S8 Fig Average of total mortality (from week 18) and fluorescence observed (from week 15) in control and treatment cages during suppression period (OX513A male adult releases in treatment cages). Total mortality represents larval/pupal mortality. Error bar represents standard error.
(TIF)

S9 Fig Hatching percent of eggs produced in control and treatment cages during the pre-release and post release period of OX513A male adults in treatment cages. The analysis indicates no significant difference between the Control and Treatment cages (p>0.05). Error bar represents standard error.
(TIF)

S10 Fig Effect of temperature and humidity on average of weekly egg production in control (A) and treatment cages before and after OX513A release (B & C) during week 1–34. *The temperature / humidity represented here is average of maximum / minimum during the week (Wednesday to Tuesday) and average for control and treatment cages.
(TIF)

S1 Table Pupal width measurement (mm) on cephalothoracic region. (PDF)

S2 Table Measurements of right wing of OX513A and AWD strain *Ae. aegypti* male adults. (PDF)

S3 Table Reproductivity of adult females recaptured post mating period in field cages. (PDF)

S4 Table Mating competitiveness between *Ae. aegypti* male adults of transgenic OX513A and wild type AWD strain with wild type AWD strain *Ae. aegypti*. (PDF)

S5 Table Mean egg count of *Ae. aegypti* AWD strain mosquitoes in control and treatment cages during the population establishment and stabilization period (week 1 to 14). (PDF)

S6 Table Average egg count of *Ae. aegypti* AWD strain mosquito in control and treatment cages during the population suppression period, post initiation of OX513A male adult releases in treatment cages from week 15 to 34. (PDF)

S7 Table Male adult sampling weekly by BG sentinel traps in paired cage units during pre-release of OX513A in treatment cages. (PDF)

S8 Table Female adult sampling weekly by BG sentinel traps in paired cage units during pre-release of OX513A in treatment cages. (PDF)

S9 Table Correlation between egg laying and temperature and humidity in control and treatment cages (before and after OX513A release). (PDF)
