# Supplementary figures and images for: Elimination of a closed population of the yellow fever mosquito, Aedes aegypti, through releases of self-limiting male mosquitoes
Source: PLoS Negl Trop Dis. 2022 May 16;16(5):e0010315. doi: 10.1371/journal.pntd.0010315 (PMC9135344; doi:10.1371/journal.pntd.0010315)

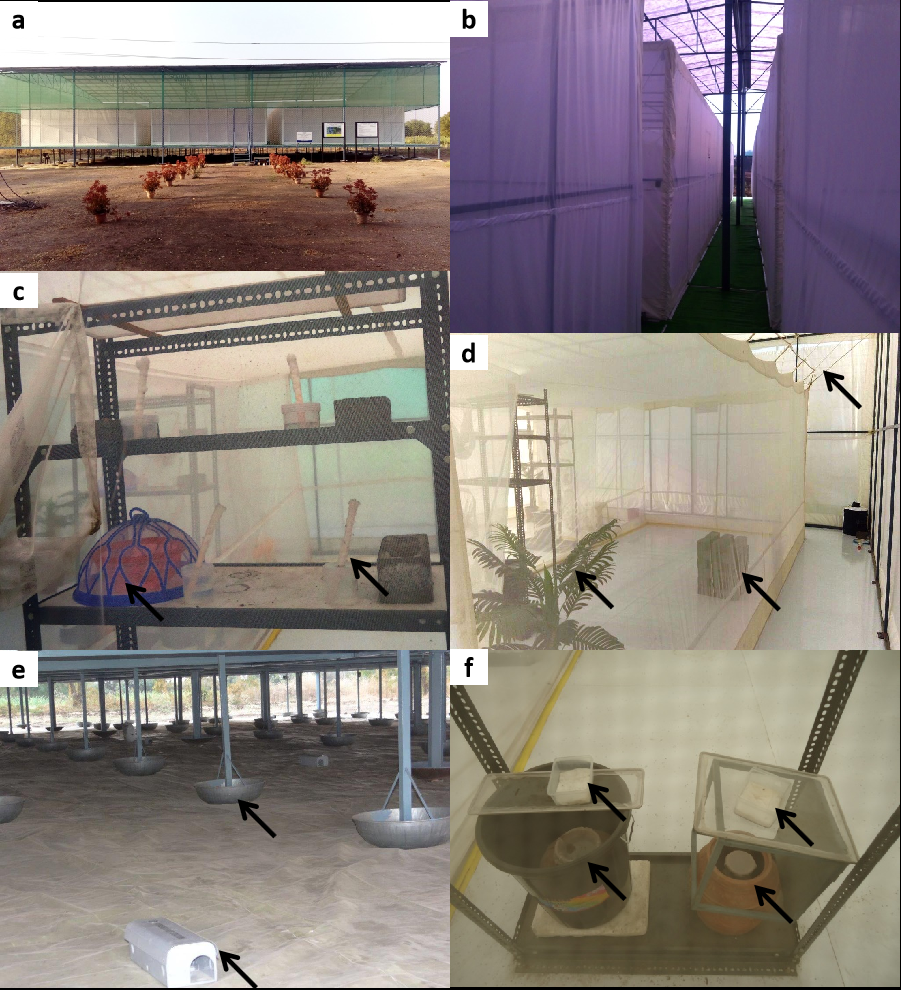

Supplement: S1 Fig — (TIF) [file pntd.0010315.s001.tif]

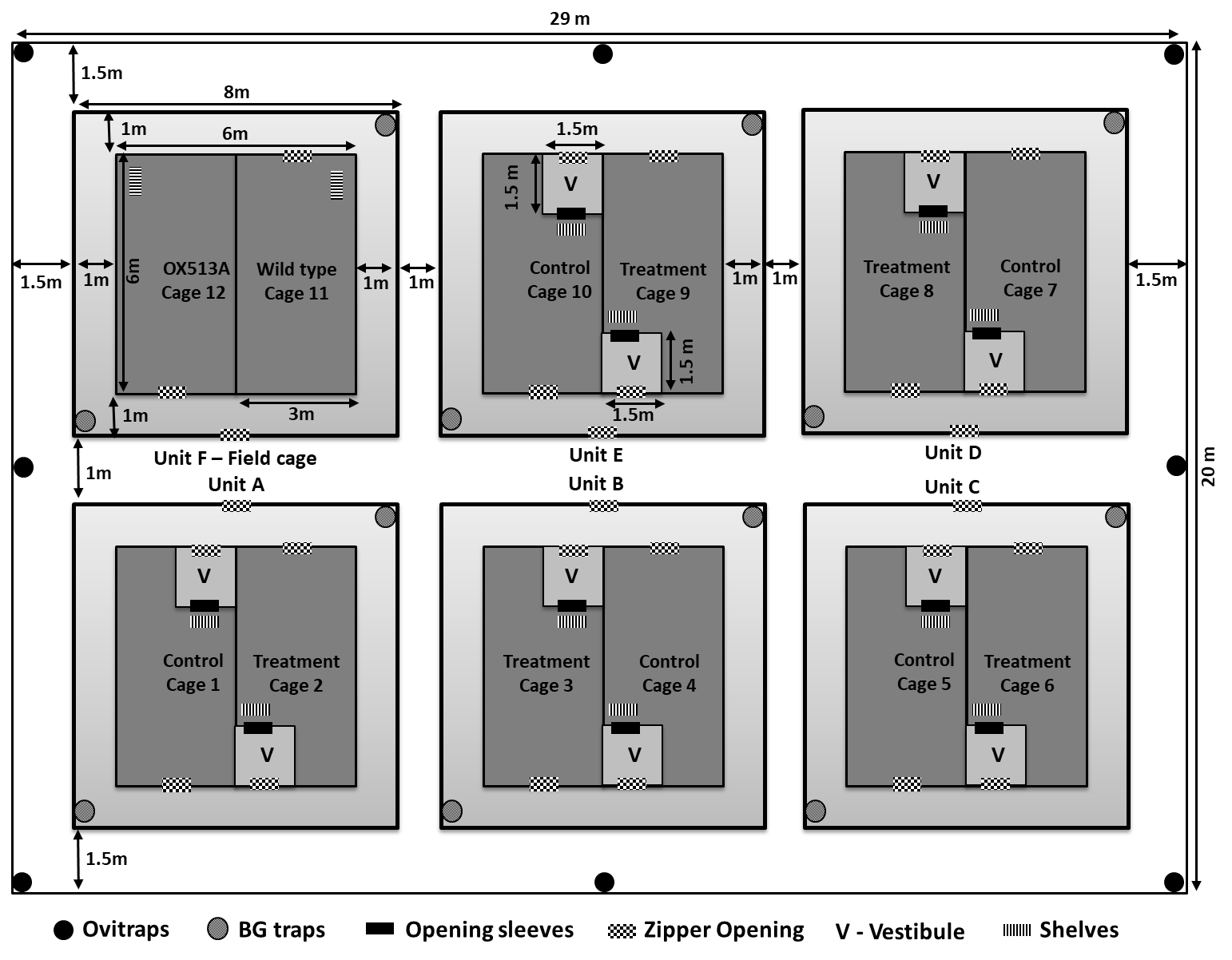

Supplement: S2 Fig — (TIF) [file pntd.0010315.s002.tif]

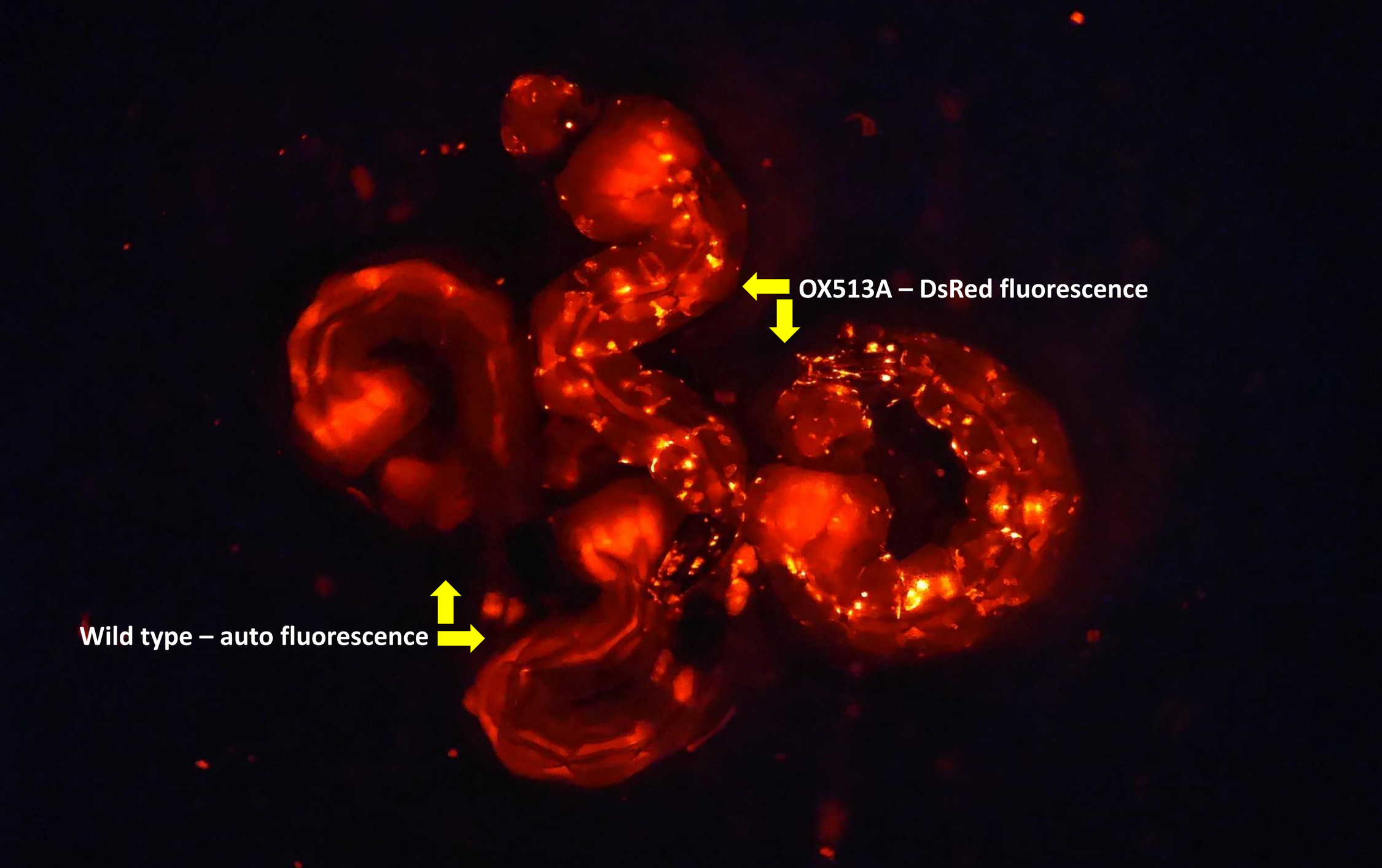

Supplement: S3 Fig — OX513A larvae display bright punctate fluorescence throughout the larva, which is distinct from auto fluorescence from the larval gut that is also observed in the AWD larvae. (TIF) [file pntd.0010315.s003.tif]

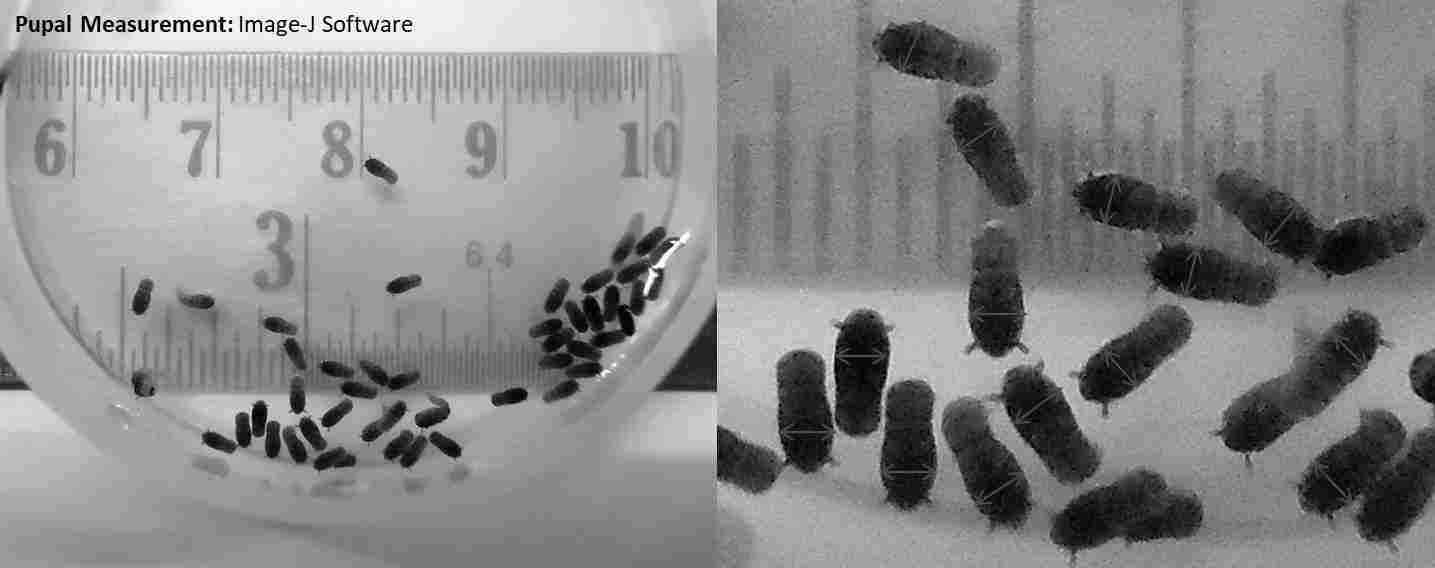

Supplement: S4 Fig — (TIF) [file pntd.0010315.s004.tif]

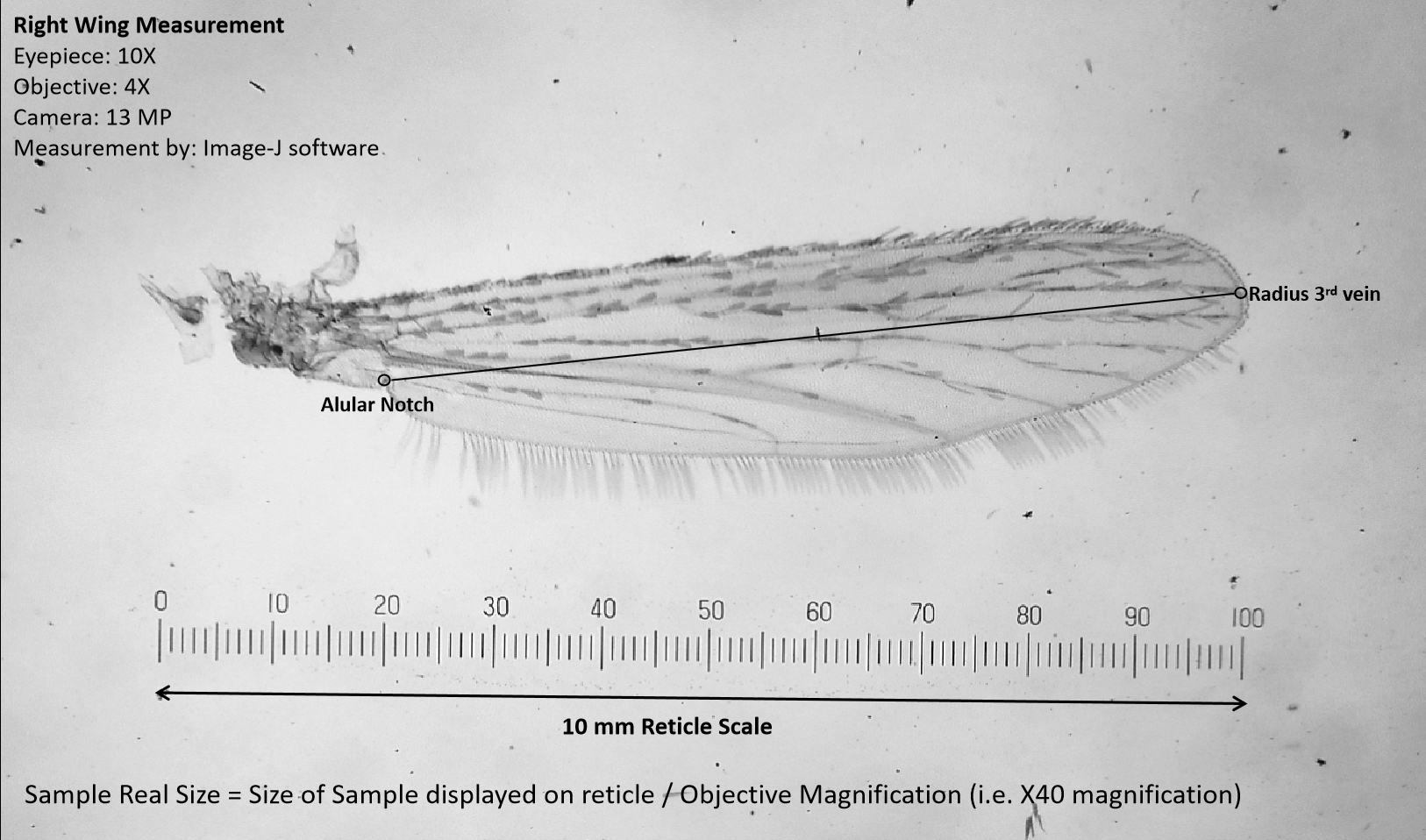

Supplement: S5 Fig — (TIF) [file pntd.0010315.s005.tif]

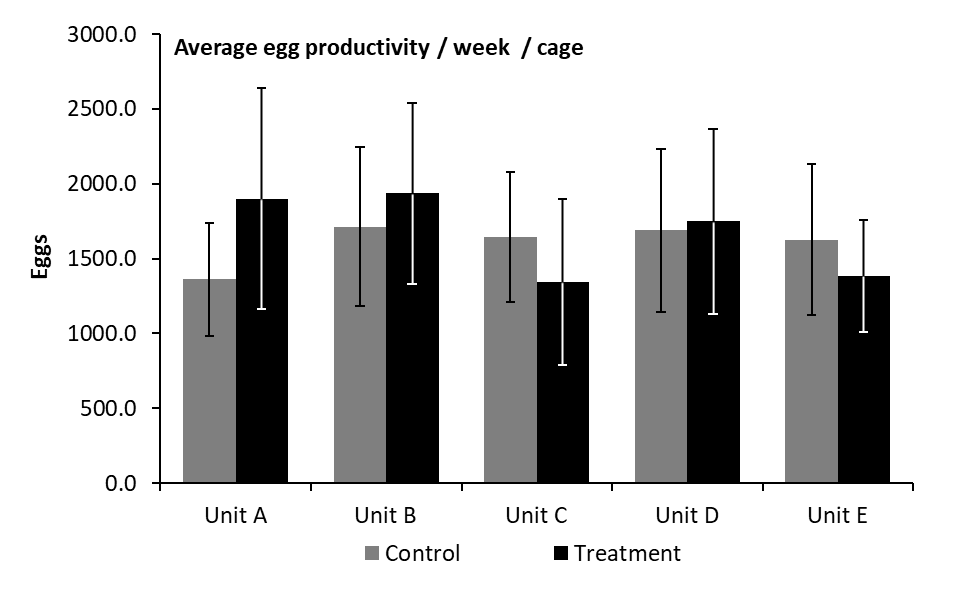

Supplement: S6 Fig — (TIF) [file pntd.0010315.s006.tif]

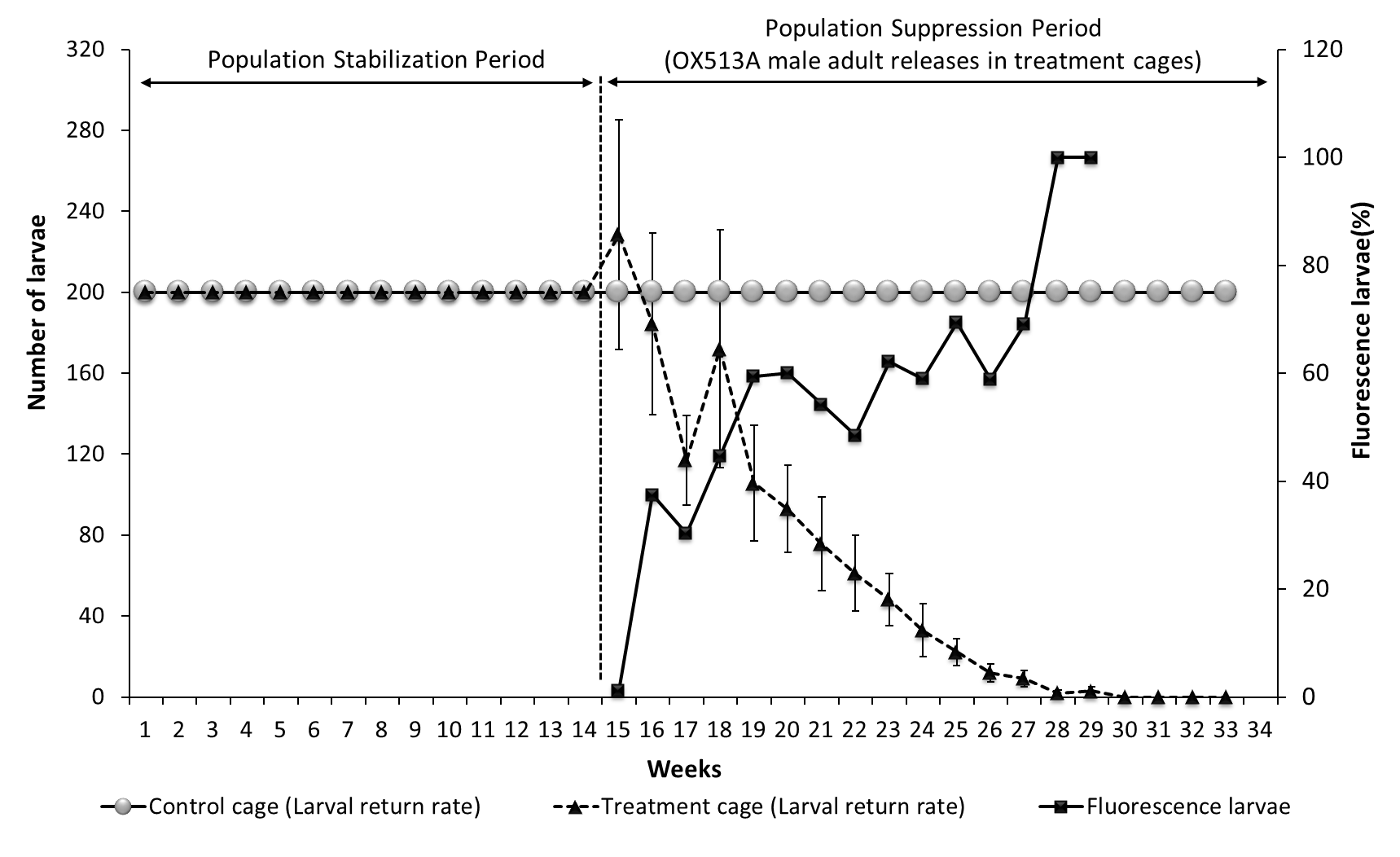

Supplement: S7 Fig — Post OX513A male adult release initiation in treatment cages and following first observation of introgression of OX513A based on fluorescence screening of progeny, the larval return rate was calculated for the treatment cage in proportion to paired control cage. Error bar represents standard error. (TIF) [file pntd.0010315.s007.tif]

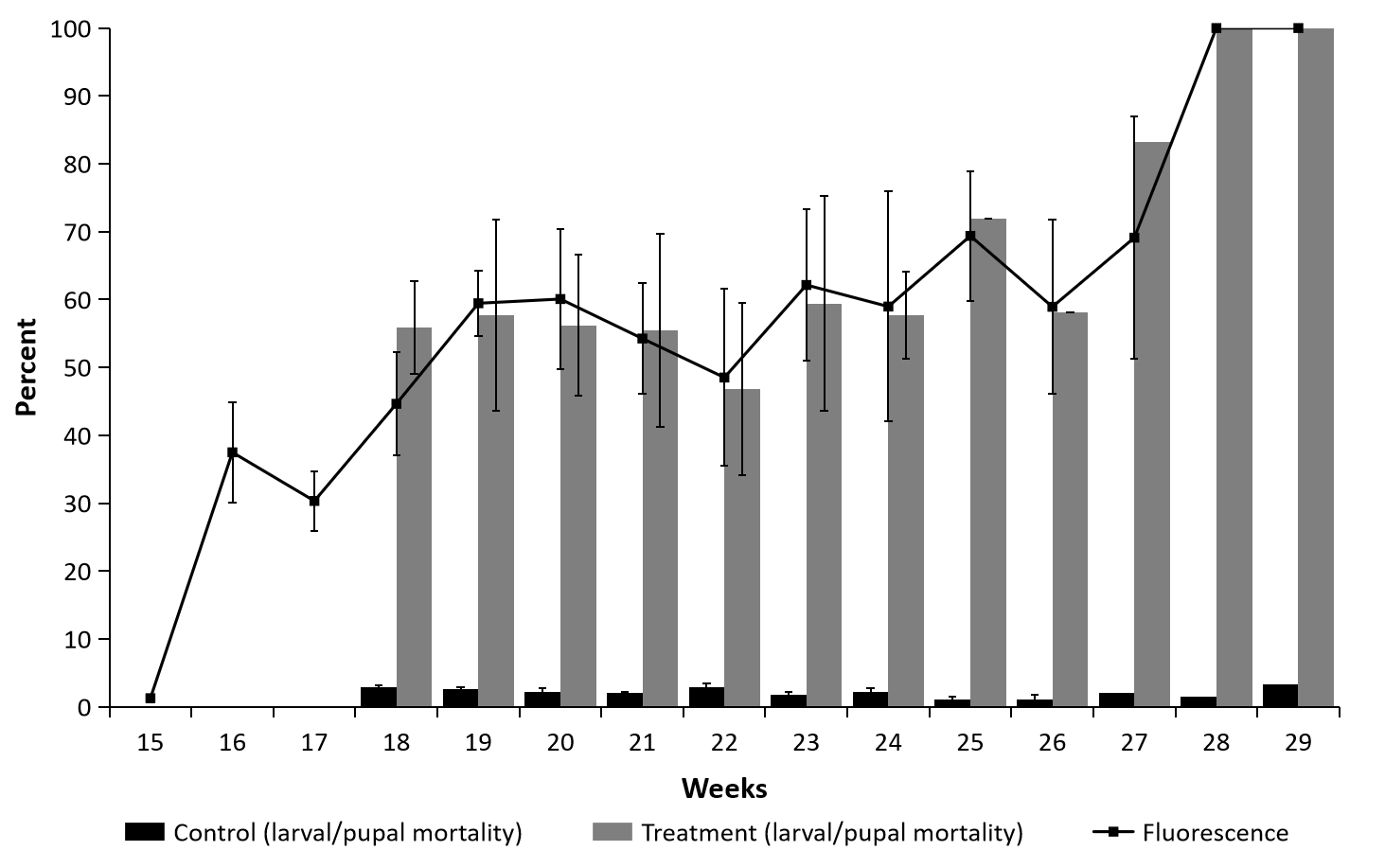

Supplement: S8 Fig — Total mortality represents larval/pupal mortality. Error bar represents standard error. (TIF) [file pntd.0010315.s008.tif]

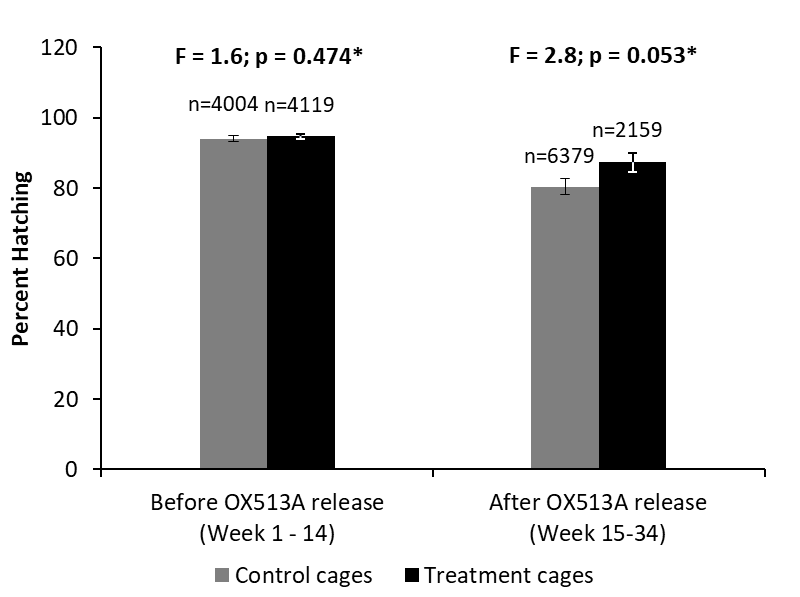

Supplement: S9 Fig — The analysis indicates no significant difference between the Control and Treatment cages (p>0.05). Error bar represents standard error. (TIF) [file pntd.0010315.s009.tif]

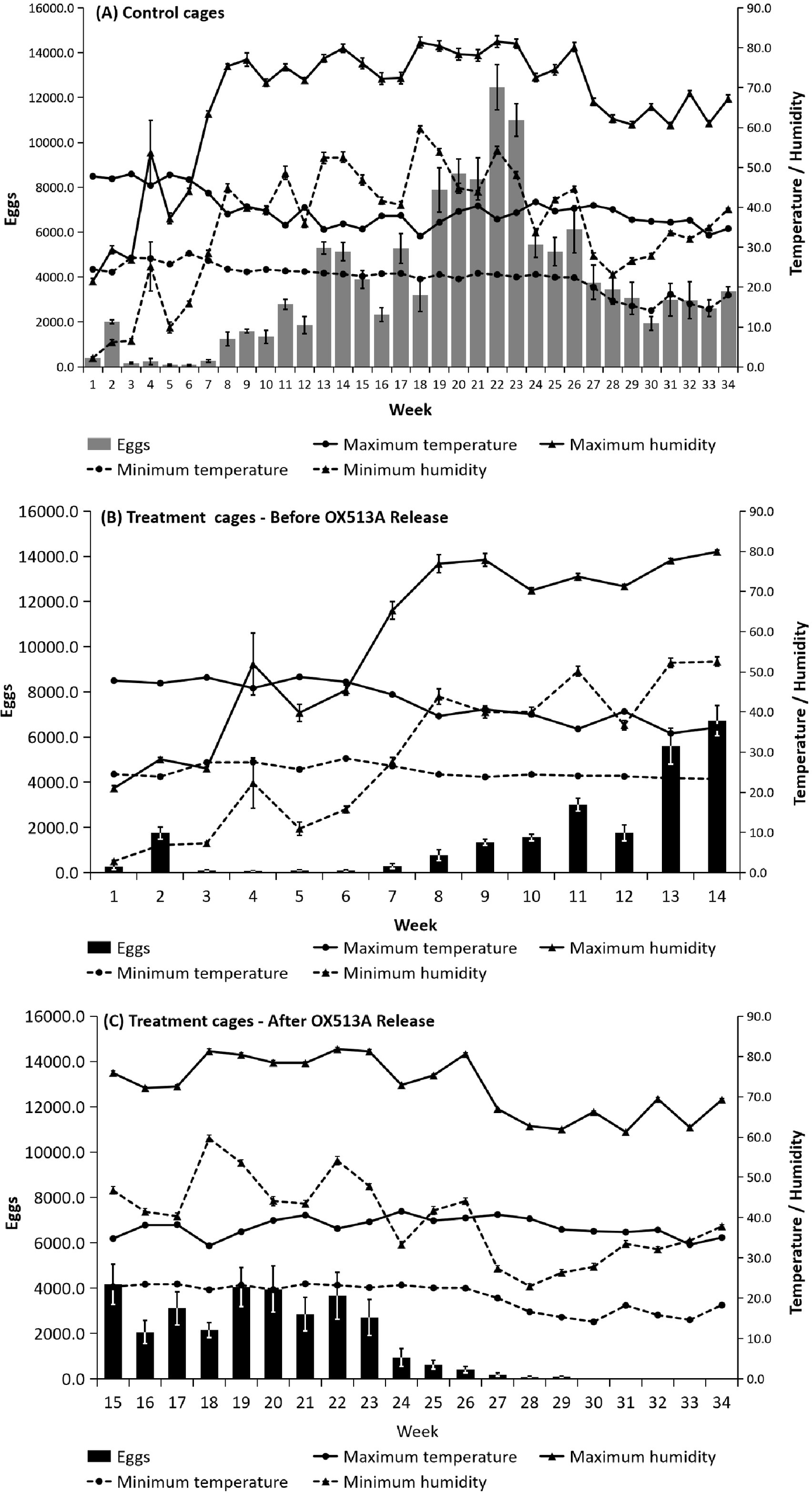

Supplement: S10 Fig — *The temperature / humidity represented here is average of maximum / minimum during the week (Wednesday to Tuesday) and average for control and treatment cages. (TIF) [file pntd.0010315.s010.tif]
