# Supplementary material for: Elimination of a closed population of the yellow fever mosquito, Aedes aegypti, through releases of self-limiting male mosquitoes
Source: PLoS Negl Trop Dis. 2022 May 16;16(5):e0010315. doi: 10.1371/journal.pntd.0010315 (PMC9135344; doi:10.1371/journal.pntd.0010315)
Supplement: S1 Table — (PDF) [file pntd.0010315.s011.pdf]

S1 Table

Dorsal side thoracic width of male pupae		F value	*Sig. (2-tailed) p value
**OX513A pupae Mean ± SE**	**AWD pupae Mean ± SE**		
0.981±0.010 (27)	0.977±0.012 (27)	0.951	0.789

*Higher value (>0.05) of Sig (2-Tailed) indicate no significant difference between AWD and OX513A strain male pupae size by Independent T Test.
Figures in the parentheses indicate total number of pupae.
