# Supplementary material for: Elimination of a closed population of the yellow fever mosquito, Aedes aegypti, through releases of self-limiting male mosquitoes
Source: PLoS Negl Trop Dis. 2022 May 16;16(5):e0010315. doi: 10.1371/journal.pntd.0010315 (PMC9135344; doi:10.1371/journal.pntd.0010315)
Supplement: S2 Table — (PDF) [file pntd.0010315.s012.pdf]

S2 Table

Right wing measurement of male adults		F value	*Sig. (2-tailed) p value
**OX513A Mean ± SE**	**AWD Mean ± SE**		
2.04±0.01 (25)	2.02±0.02 (25)	0.517	0.447

*Higher value (>0.05) of Sig (2-Tailed) indicate no significant difference between right wing of AWD and OX513A strain male adults by Independent T Test.
Figures in the parentheses indicate total number of adults sampled for measurement.
