# Supplementary material for: Elimination of a closed population of the yellow fever mosquito, Aedes aegypti, through releases of self-limiting male mosquitoes
Source: PLoS Negl Trop Dis. 2022 May 16;16(5):e0010315. doi: 10.1371/journal.pntd.0010315 (PMC9135344; doi:10.1371/journal.pntd.0010315)
Supplement: S3 Table — (PDF) [file pntd.0010315.s013.pdf]

S3 Table

Percent females recaptured	Percent females survived	Percent females laid eggs	Unhatched egg batches	Average eggs laid per female	Percent egg hatching
79.7 (n=239)	61 (n=183)	60.3 (n=181)	*4.0 (n=88)	58.0 (n=10496)	76.6 (n=8044)

*Figure represents number of females laid eggs, which did not hatch.
