# Supplementary material for: Elimination of a closed population of the yellow fever mosquito, Aedes aegypti, through releases of self-limiting male mosquitoes
Source: PLoS Negl Trop Dis. 2022 May 16;16(5):e0010315. doi: 10.1371/journal.pntd.0010315 (PMC9135344; doi:10.1371/journal.pntd.0010315)
Supplement: S4 Table — (PDF) [file pntd.0010315.s014.pdf]

S4 Table

**Mosquito strains**	**OX513A Strain ♂ Mean±SE**	**Wild type strain ♂ Mean±SE**	**Double mating [Wild type & OX513A strain] ♂ Mean±SE**	**F value (df)**	**p value**	
Number of female adults mated with	24.0±7.5 (n= 72)	28.0±5.5 (n= 84)	6.3±1.9 (n= 19)	4.4 (2)	0.067	
Percent female adults mated with	41.1	48.0	10.9	--	--	
**#Independents Sample T-test**	F value = 0.681; Sig. (2-tailed)=0.691					
Relative Mating Index	0.41	0.48	0.10	--	--	
Relative Mating Index [calculated excluding double mated females]	0.46	0.54	--	--	--	
**Observed Mating Index**	**Expected Mating index**	**Chi-square test (X^2)**	**p value**	***df***	**--**	**--**
72 : 84 : 19 [OX513A ♂: WT ♂: Double mating (OX513A & WT ♂)]	87.5: 87.5: 0	2.886	0.2362	2	--	--

Means (±SE) are the results of 3 replicates of mating experiment conducted at a ratio of 100:100:100 respectively.

#Higher value (>0.05) of Sig (2-Tailed) indicate no significant difference between mean number of female adults mated with OX513A and AWD male adult mosquitoes by Independent T Test.
