# Supplementary material for: Elimination of a closed population of the yellow fever mosquito, Aedes aegypti, through releases of self-limiting male mosquitoes
Source: PLoS Negl Trop Dis. 2022 May 16;16(5):e0010315. doi: 10.1371/journal.pntd.0010315 (PMC9135344; doi:10.1371/journal.pntd.0010315)
Supplement: S5 Table — (PDF) [file pntd.0010315.s015.pdf]

S5 Table

Control / Treatment	Mean / SE	Week													
		1	2	3	4	5	6	7	8	9	10	11	12	13	14
*Control (Eggs)	**Mean	376.2^{a}	2012.0^{bc}	165.2^{a}	245.4^{a}	94.8^{a}	79.4^{a}	263.2^{a}	1245.6^{b}	1593.8^{b}	1341.4^{b}	2790.0^{c}	1862.8^{b}	5302.2^{d}	5130.0^{d}
	SE	80.7	85.9	35.9	137.0	43.7	20.6	73.0	299.7	93.6	295.7	227.3	390.9	269.4	404.5
**One-way ANOVA**	df – 13; F value -64.1; *p value* - <0.000														
*Treatment (Eggs)	**Mean	261.0^{ab}	1751.0^{bc}	37.4^{a}	18.2^{a}	47.4^{a}	90.8^{a}	289.2^{ab}	777.2^{ab}	1349.4^{ab}	1553.6^{ab}	3011.8^{c}	1761.0^{bc}	5605.8^{d}	6727.0^{d}
	SE	124.3	263.3	37.4	11.2	19.7	54.4	108.2	240.4	128.6	152.2	278.2	343.1	796.9	661.5
**One-way ANOVA**	df – 13; F value – 42.7; *p value* - <0.000														
**Independent sample T-test**															
F value		2.69	6.73	.016	76.01	6.16	3.82	.29	.09	.46	2.11	.74	.00	3.46	0.20
p value		.140	.032	.903	.000	.038	.086	.605	.770	.516	.185	.416	.978	.100	0.669
#Sig (2-Tailed)		.459	.374	.039	.137	.352	.850	.847	.257	.163	.541	.554	.850	.728	0.073
**Test for normality - Kolmogorov-Smirnov Test**															
df		10	10	10	10	10	10	10	10	10	10	10	10	10	10
##*p value*		0.20	0.20	0.112	<0.001	2.00	0.153	2.00	2.00	2.00	2.00	2.00	0.032	2.00	2.00

*Control cages (cage 1, 4, 5, 7 & 10); treatment cages (cage 2, 3, 6, 8, & 9)

**Figures within the rows indicated with same alphabets show non-significant difference between the values by one-way ANOVA following Tukey's B Test.

#Higher value (>0.05) of Sig (2-Tailed) indicate no significant difference between treatment and control groups in mean egg counts in respective week by Independent T Test.

##The high value (>0.05) of Sig (2-Tailed) indicate normal egg production in the cages weekly for test for normality by Kolmogorov-Smirnov test.
