# Supplementary material for: Elimination of a closed population of the yellow fever mosquito, Aedes aegypti, through releases of self-limiting male mosquitoes
Source: PLoS Negl Trop Dis. 2022 May 16;16(5):e0010315. doi: 10.1371/journal.pntd.0010315 (PMC9135344; doi:10.1371/journal.pntd.0010315)
Supplement: S6 Table — (PDF) [file pntd.0010315.s016.pdf]

S6 Table

	Control	Treatment	Independent T-test		
Week	Mean ± SE	Mean ± SE	F value	*p value*	#Sig (2-Tailed)
15	3899.0±386^{ab}	4178.6±891^{d}	4.0	.08	.781
16	2329.6±298^{a}	2062.4±512^{abcd}	6.4	.04	.664
17	5277.2±667^{abc}	3120.8±730^{abcd}	0.1	.75	.061
18	3201.4±745^{ab}	2146.2±328^{abcd}	3.5	.10	.231
19	7888.0±976^{cd}	4053±875^{cd}	0.4	.55	.019
20	8601.2±660^{cd}	3957.2±1024^{d}	1.3	.28	.005
21	8371.2±930^{cd}	2852.6±739^{abcd}	0.3	.61	.002
22	12464.6±1004^{e}	3655.8±1035^{bcd}	0.0	.95	.000
23	11002.6±734^{de}	2714.2±800^{abcd}	0.5	.51	.000
24	5457.0±566^{abc}	939.6±396^{abc}	0.3	.61	.000
25	5138.8±617^{abc}	624.8±202^{ab}	3.3	.11	.000
26	6126.2±1054^{bc}	414.6±149^{ab}	5.1	.05	.001
27	3772.0±752^{ab}	181.6±75^{ab}	27.8	.00	.001
28	3461.0±669^{ab}	31±19.7^{a}	8.5	.02	.001
29	3068.3±712^{ab}	35±20.4^{a}	51.2	.00	.005
30	1935.8±304^{a}	0^{a}	10.8	.02	.001
31	3001.0±725^{ab}	0^{a}	10.0	.02	.006
32	2972.5±831^{ab}	0^{a}	8.1	.03	.012
33	2609.5±374^{a}	0^{a}	--	--	0.02
34	3356.5±229^{a}	0^{a}	--	--	0.005
*One-way ANOVA (Tukey' b test)					
Df	19	19			
F value	16.2	6.9			
p value	<0.000	<0.000			

#Lower value for Sig (2-Tailed) indicate significant difference between treatment and control groups in mean egg counts in respective week following independent sample T-test.
*Figures within the columns indicated by same alphabets show non-significant difference between the values following one-way ANOVA by Tukey's b test.
