# Supplementary material for: Elimination of a closed population of the yellow fever mosquito, Aedes aegypti, through releases of self-limiting male mosquitoes
Source: PLoS Negl Trop Dis. 2022 May 16;16(5):e0010315. doi: 10.1371/journal.pntd.0010315 (PMC9135344; doi:10.1371/journal.pntd.0010315)
Supplement: S7 Table — (PDF) [file pntd.0010315.s017.pdf]

S7 Table

	Before OX513A release period in treatment cages				
	**Unit A**	**Unit B**	**Unit C**	**Unit D**	**Unit E**
**Male adult sampling**					
Mann-Whitney U (U value)	38	37	38	31.5	25.5
*Asymptotic Sig. (2-tailed) p value	0.83	0.76	0.82	0.42	0.18
**Female adult sampling**					
Mann-Whitney U (U value)	31	31	29.5	21	39
*Asymptotic Sig. (2-tailed) p value	0.40	0.40	0.33	0.08	0.89

*Higher value (p>0.05) indicate no significant difference between treatment and control groups for number of adult analysis
