# Supplementary material for: Elimination of a closed population of the yellow fever mosquito, Aedes aegypti, through releases of self-limiting male mosquitoes
Source: PLoS Negl Trop Dis. 2022 May 16;16(5):e0010315. doi: 10.1371/journal.pntd.0010315 (PMC9135344; doi:10.1371/journal.pntd.0010315)
Supplement: S8 Table — (PDF) [file pntd.0010315.s018.pdf]

S8 Table

	After OX513A release period in treatment cages				
	**Unit A**	**Unit B**	**Unit C**	**Unit D**	**Unit E**
**Male adult sampling**					
Mann-Whitney U (U value)	0	0	0	0	0
*Asymptotic Sig. (2-tailed) p value	<0.001	<0.001	<0.001	<0.001	<0.001
**Female adult sampling**					
Mann-Whitney U (U value)	53	117	49.5	95	120.5
*Asymptotic Sig. (2-tailed) p value	<0.001	0.06	0.009	0.012	0.031

*Lower value ($p<0.05$) indicate significant difference between treatment and control groups for number of adult analysis.
