# Supplementary material for: Elimination of a closed population of the yellow fever mosquito, Aedes aegypti, through releases of self-limiting male mosquitoes
Source: PLoS Negl Trop Dis. 2022 May 16;16(5):e0010315. doi: 10.1371/journal.pntd.0010315 (PMC9135344; doi:10.1371/journal.pntd.0010315)
Supplement: S9 Table — (PDF) [file pntd.0010315.s019.pdf]

S9 Table

[##]Temperature / Humidity		Egg production vs *Temperature/Humidity		
		Control Cages (Week 1-34)	Treatment cages	
			Before OX513A release (Week 1-14)	After OX513A release (Week 15-34)
**Temperature**				
Maximum	Pearson Correlation (r value)	*-0.416	*-0.786	[#]-0.013
	Sig. (2-tailed)	0.014	0.001	0.957
Minimum	Pearson Correlation (r value)	-0.14	*-0.665	**0.739
	Sig. (2-tailed)	0.429	0.009	<0.001
**Humidity**				
Maximum	Pearson Correlation (r value)	**0.619	**0.568	**0.737
	Sig. (2-tailed)	<0.001	0.034	<0.001
Minimum	Pearson Correlation (r value)	**0.628	**0.713	**0.753
	Sig. (2-tailed)	<0.001	0.004	<0.001

*Negative correlation; **Positive correlation; [#]correlation is non-significant

[##]The temperature / humidity represented is average of maximum / minimum during the week (Wednesday to Tuesday) and average for control and treatment cages.

[##]Weekly average was considered from Wednesday to Tuesday as ovitraps (eggs laid by female mosquitoes in ovitraps placed in the cages) were retrieved on Tuesday.
