# Supplementary material for: Elimination of a closed population of the yellow fever mosquito, Aedes aegypti, through releases of self-limiting male mosquitoes
Source: PLoS Negl Trop Dis. 2022 May 16;16(5):e0010315. doi: 10.1371/journal.pntd.0010315 (PMC9135344; doi:10.1371/journal.pntd.0010315)
Supplement: Attachment — Submitted filename: Review of.pdf [file pntd.0010315.s020.pdf]

# Review of:

# Eradication of a closed population of the yellow fever mosquito, Aedes aegypti, through releases of self-limiting male mosquitoes

By

Patil et al.

This is a nicely done study but a daunting undertaking given its long duration and intensive activity required on a consistent basis. The design and write up are very good for the most part and I have no problem with the statistical analysis generally. Well done. Go on holiday.

This study reports the continued testing of the OX513A strain but in a new country as part of the testing procedure that is generally recommended.

In several places in the Discussion I have noted repetition of results that need to be deleted.

I have made many comments and corrections in the marked-up PDF.

In the figures that show the trends for the populations, I suggest that they authors eliminate symbols. The inflections are clear so no symbols are needed.

One of my greatest concerns influences the presentation and analysis of the results throughout. Specifically, the restocking of the progeny coming from the cage was performed with 200 L2, but there is no indication of how many adults this represents because no survival data is presented. On the other hand, adult OX513A adult males were introduced so their numbers are known with certainty. This is not an experimental flaw unless that data was not collected, but it does influence what the over-flooding ratio is and an attempt to address this must be made. It does not affect the fact that a stable population was established, but during the release phase, we cannot know what the return:release ratio was. In the Discussion, the authors suggest it was a 10:1 ratio but that is not a value that can be determined from the data presented.

Similarly, no data is provided that describes the sex ratio of the L2s or the AWD stock generally. *Aedes aegypti* stocks are frequently male-biased, but this is highly variable. If the authors did not determine this and still have the AWD stock, they should measure this value and adjust their reporting and calculations

accordingly. While for some species, the sex ratio seldom varies from a 1:1 and this assumption is acceptable, for *Ae. aegypti*, it is not.

The larval mortality and ignorance (or lack of reporting) of the AWD sex ratio introduces a great deal of uncertainty regarding the actual demographics of the established population and subsequent male release ratios. The authors should do whatever they can to correct this or soften some of the certainty about the demographics and release ratios.
