# Supplementary material for: Elimination of a closed population of the yellow fever mosquito, Aedes aegypti, through releases of self-limiting male mosquitoes
Source: PLoS Negl Trop Dis. 2022 May 16;16(5):e0010315. doi: 10.1371/journal.pntd.0010315 (PMC9135344; doi:10.1371/journal.pntd.0010315)
Supplement: Attachment — Submitted filename: Review of eradication Aedes aegypti.pdf [file pntd.0010315.s024.pdf]

Review of: Eradication of a closed population of the yellow fever mosquito, *Aedes aegypti*, through releases of self-limiting male mosquitoes

By Patil et al.

This is a nicely presented set of experiments that demonstrate the potential of the OX513A *Aedes aegypti* strain for suppression of wild-type mosquitoes. This has been demonstrated previously in other places, but the studies conducted here are part of the phases for testing genetically modified mosquitoes released by WHO, and they provide confidence that what was observed elsewhere is likely to be observed in India.

The studies were conducted using a well-accepted method for cage testing and the results are supported by the data that was presented. All in all, a nice study with appropriate methods and clear narrative and figures. Congratulations to the authors for successfully conducting what are often a grueling and demanding set of experiments that must be conducted flawlessly over several months.

I have made numerous specific comments in the marked-up PDF. Some are style or requests for clarification.

In keeping with the principle that M & M should contain M & M, Results, Results etc. generally, I recommend that the authors reduce the amount of the Discussion that is a repetition of Results. The repetition should be limited to only what is necessary to provide context for an interpretation or observation that is not reported in the results. I have marked a few of these instances but the practice is pervasive in the Discussion and should be reconsidered.

Moreover, the Discussion is an opportunity to describe any caveats about the interpretation of the data etc. The authors mention the Fachinelli et al. Mexico study on flightless females. They might say why the flightless female laboratory experiments (**Wise de Valdez, M. R., D. Nimmo, J. Betz, H.-F. Gong, A. A. James, L. Alphey, and W. C. Black**. **2011**. Genetic elimination of dengue vector mosquitoes. Proc. Natl. Acad. Sci. U.S.A. 108: 4772–4775.) were successful but the Mexico studies revealed deficiencies. This is the sort of discussion that will flesh out an understanding of the results obtained.

Also, the authors should check whether reference 22 should be the one above rather than the densonucleosis virus study. Not sure.
