# Supplementary material for: Elimination of a closed population of the yellow fever mosquito, Aedes aegypti, through releases of self-limiting male mosquitoes
Source: PLoS Negl Trop Dis. 2022 May 16;16(5):e0010315. doi: 10.1371/journal.pntd.0010315 (PMC9135344; doi:10.1371/journal.pntd.0010315)
Supplement: Attachment — Submitted filename: PNTD-D-20-01954_reviewer.pdf [file pntd.0010315.s025.pdf]

PLOS Neglected Tropical Diseases

# Eradication of a closed population of the yellow fever mosquito, Aedes aegypti, through releases of self-limiting male mosquitoes

--Manuscript Draft--

| | |
|---|---|
| Manuscript Number: | PNTD-D-20-01954 |
| Full Title: | Eradication of a closed population of the yellow fever mosquito, Aedes aegypti, through releases of self-limiting male mosquitoes |
| Short Title: | Eradication of Aedes aegypti mosquitoes |
| Article Type: | Research Article |
| Keywords: | Aedes aegypti; dengue; OX513A; genetically modified organisms; living modified organisms |
| Abstract: | Establishment of novel mosquito control technologies such as the use of genetically engineered insects typically involves phased testing to generate robust data-sets that support its safe and effective use as a vector control tool. In this study, we demonstrate the ability of the transgenic self-limiting OX513A Ae. aegypti strain to suppress a wild type Ae. aegypti population in an outdoor containment facility in India. OX513A is a genetically engineered Ae. aegypti strain with a repressible dominant self-limiting gene. When male adult OX513A mate with wild female adults, a single copy of the self-limiting gene is inherited by all the progeny, leading to death of >95% of progeny during larval/pupal development. A wild-type population of Ae. aegypti was established and stabilized during a 14 week period in 5 paired field cage units, each consisting of control and treatment cages, followed by weekly releases of OX513A male adults to suppress the target population. The successive introductions of OX513A male adults led to a consistent decline in wild type numbers eventually resulting in the eradication of Ae . aegypti from all treated cages within 10 to 15 weeks of releases. This study demonstrates that localized Ae. aegypti eradication may be a realistic and achievable target in relatively isolated environments. |
| Additional Information: | |
| Question | Response |
| **Financial Disclosure**<br><br>Enter a financial disclosure statement that describes the sources of funding for the work included in this submission. Review the submission guidelines for detailed requirements. View published research articles from *PLOS NTDs* for specific examples.<br><br>This statement is required for submission and **will appear in the published article** if the submission is accepted. Please make sure it is accurate. | The author(s) received no specific funding for this work. |

| | |
|---|---|
| **Unfunded studies**<br><br>Enter: *The author(s) received no specific funding for this work.*<br><br>**Funded studies**<br><br>Enter a statement with the following details:<br>• Initials of the authors who received each award<br>• Grant numbers awarded to each author<br>• The full name of each funder<br>• URL of each funder website<br>• Did the sponsors or funders play any role in the study design, data collection and analysis, decision to publish, or preparation of the manuscript?<br>• **NO** - Include this sentence at the end of your statement: *The funders had no role in study design, data collection and analysis, decision to publish, or preparation of the manuscript.*<br>• **YES** - Specify the role(s) played.<br><br>* typeset | |
| **Competing Interests**<br><br>Use the instructions below to enter a competing interest statement for this submission. On behalf of all authors, disclose any competing interests that could be perceived to bias this work—acknowledging all financial support and any other relevant financial or non-financial competing interests.<br><br>This statement **will appear in the published article** if the submission is accepted. Please make sure it is accurate. View published research articles from *PLOS NTDs* for specific examples. | The authors have declared that no competing interests exist. |

| | |
|---|---|
| **NO authors have competing interests**<br><br>Enter: *The authors have declared that no competing interests exist.*<br><br>**Authors with competing interests**<br><br>Enter competing interest details beginning with this statement:<br><br>*I have read the journal's policy and the authors of this manuscript have the following competing interests: [insert competing interests here]*<br><br>* typeset | |
| **Data Availability**<br><br>Authors are required to make all data underlying the findings described fully available, without restriction, and from the time of publication. PLOS allows rare exceptions to address legal and ethical concerns. See the PLOS Data Policy and FAQ for detailed information.<br><br>A Data Availability Statement describing where the data can be found is required at submission. Your answers to this question constitute the Data Availability Statement and **will be published in the article**, if accepted. | Yes - all data are fully available without restriction |

| | |
|---|---|
| **Important:** Stating 'data available on request from the author' is not sufficient. If your data are only available upon request, select 'No' for the first question and explain your exceptional situation in the text box.<br><br>Do the authors confirm that all data underlying the findings described in their manuscript are fully available without restriction? | |
| **Describe where the data may be found in full sentences. If you are copying our sample text, replace any instances of XXX with the appropriate details.**<br><br>• If the data are **held or will be held in a public repository**, include URLs, accession numbers or DOIs. If this information will only be available after acceptance, indicate this by ticking the box below. For example: *All XXX files are available from the XXX database (accession number(s) XXX, XXX.*).<br>• If the data are all contained **within the manuscript and/or Supporting Information files**, enter the following: *All relevant data are within the manuscript and its Supporting Information files.*<br>• If neither of these applies but you are able to provide **details of access elsewhere**, with or without limitations, please do so. For example:<br><br>*Data cannot be shared publicly because of [XXX]. Data are available from the XXX Institutional Data Access / Ethics Committee (contact via XXX) for researchers who meet the criteria for access to confidential data.*<br><br>*The data underlying the results presented in the study are available* | All relevant data are within the manuscript and its Supporting Information files. |

| | |
|---|---|
| *from (include the name of the third party and contact information or URL).*<br>• This text is appropriate if the data are owned by a third party and authors do not have permission to share the data.<br><br>* typeset | |
| Additional data availability information: | |

Manuscript Click here to access/download;Manuscript;Manuscript.docx

# Eradication of a closed population of the yellow fever mosquito, *Aedes aegypti*, through releases of self-limiting male mosquitoes

**Short title:** Eradication of *Aedes aegypti* mosquitoes

Prabhakargouda B Patil[1¶a]*, Shaibal Kumar Dasgupta[1#b], Kevin Gorman[2$], Angela Pickl-Herk[2$], Mirel Puinean[2$], Andrew McKemey[2$], Bharat Char[1@a], Usha B Zehr[1@] and Shirish R Barwale[1@]

[1]GBIT Limited, Dawalwadi, Badnapur, Jalna-431203, Maharashtra, India

[2]Oxitec Limited, 71 Innovation Drive, Milton Park, Abingdon, Oxfordshire, OX14 4RQ, United Kingdom

[a]Current address: Maharashtra Hybrid Seeds Company Pvt. Ltd., Dawalwadi, Badnapur, Jalna-431203, Maharashtra, India

[b]Current address: Tata Institute for Genetics and Society, New inStem Building, NCBS campus, GKVK Post, Bellary road, Bengaluru, India – 560065

*Corresponding author

Email: prabhakar.patil@mahyco.com (PBP); dr.prabhakarpatil8@gmail.com (PBP)

[¶]The author contributed to conceptualization, data curation, methodology, investigation, formal analysis, visualization and writing-original draft preparation.

[#]The author contributed to conceptualization, methodology, project administration, validation and writing-review and editing.

[$]These authors contributed to conceptualization, data curation, formal analysis, methodology, validation, and writing-review and editing.

[@]These authors contributed to conceptualization, methodology, resources, writing-review and editing.

## Abstract

Establishment of novel mosquito control technologies such as the use of genetically engineered insects typically involves phased testing to generate robust data-sets that support its safe and effective use as a vector control tool. In this study, we demonstrate the ability of the transgenic self-limiting OX513A *Ae. aegypti* strain to suppress a wild type *Ae. aegypti* population in an outdoor containment facility in India. OX513A is a genetically engineered *Ae. aegypti* strain with a repressible dominant self-limiting gene. When male adult OX513A mate with wild female adults, a single copy of the self-limiting gene is inherited by all the progeny, leading to death of >95% of progeny during larval/pupal development. A wild-type population of *Ae. aegypti* was established and stabilized during a 14 week period in 5 paired field cage units, each consisting of control and treatment cages, followed by weekly releases of OX513A male adults to suppress the target population. The successive introductions of OX513A male adults led to a consistent decline in wild type numbers eventually resulting in the eradication of *Ae. aegypti* from all treated cages within 10 to 15 weeks of releases. This study demonstrates that localized *Ae. aegypti* eradication may be a realistic and achievable target in relatively isolated environments.

## Author Summary

*Aedes aegypti* L. species is the primary vector responsible for transmission of the dengue virus worldwide including chikungunya, yellow fever and emerging zika virus. The experiment presented in the manuscript represents an important study undertaken to demonstrate suppression of the wild type *Ae. aegypti* population in large field cages under natural environmental settings (Physically-contained field cage facility) by sustained releases of male adults of OX513A *Ae. aegypti* strain. This investigation is a phase-2 contained study as per the World Health Organization guidelines for evaluation of genetically modified organisms and was recommended by the Indian regulatory board. This experiment first time

demonstrated suppression of wild type *Ae. aegypti* population under contained facility. The prospect of the project is to demonstrate and implement the technology for controlling/suppression of the *Ae. aegypti* vector in the open field environment.

# Introduction

Advancements in biotechnology have paved the way for novel techniques targeted towards safeguarding human and environmental health, such as the use of genetically modified organisms (GMOs), also termed living modified organisms (LMOs). LMO's have become essential tools for providing global food security and also underpin the management of specific human diseases [1]. *Aedes aegypti* (Linnaeus), commonly known as the yellow fever mosquito, is a primary vector of several dangerous arboviruses including those that cause dengue, chikungunya and Zika in humans and animals [2-3]. In India, outbreaks of dengue and chikungunya have been increasing in frequency in recent decades with annual reported cases of 188,401 and 67,769, respectively, during the year 2017 (NVBDCP [4]). A recent study estimated that due to under-reporting, the number of dengue cases documented is likely over 200 times lower than the actual occurrence of dengue infections [5].

The development of vaccines against dengue virus has shown some promising results under trials, however, implementation has identified difficulties in mitigating risks, due to the complications caused by incomplete protection [6]. The presence of multiple dengue viral serotypes can increase the likelihood of severe forms of dengue viral infection (i.e. dengue hemorrhagic fever and dengue shock syndrome) if a previously infected person is subsequently infected with a different strain [7]. The World Health Organization has reported that controlling vector populations is the key factor to combat transmission of dengue [8].

Efforts to manage *Ae. aegypti* populations using current methods pose several challenges due to their breeding behavior, anthropophilic nature, and multiple host biting behavior, which together result in an ability to transmit disease with a threshold level as low

as 0.5 female *Ae. aegypti*/person, which corresponds to 0.25 pupae/person [9-10]. Current control methods mostly involve the application of non-specific broad-spectrum synthetic insecticides like pyrethroids, carbamates and DDT, in spite of their identified risks for resistance development and potential for undesirable effects on non-target organisms [11]. One promising alternative to conventional vector control strategies is the use of genetically engineered mosquitoes, such as the OX513A *Ae. aegypti* strain, also known as the Friendly™ mosquito [12-15]. OX513A is a self-limiting strain with a tetracycline-repressible dominant self-limiting gene. Progeny fathered by OX513A male adult mosquitoes inherit a copy of the self-limiting gene, with >95% dying before reaching adulthood in the absence of the antidote, tetracycline [16]. This self-limiting approach means that there is no significant persistence of the transgene and monitoring following field releases of OX513A has documented the complete disappearance of the transgene from the environment [15]. In addition, OX513A mosquitoes have been shown to be susceptible to currently used insecticides [17]. In 2016, the WHO Vector Control Advisory Group (VCAG) recommended the use of OX513A for pilot deployments in operational contexts in response to the Public Health of International Concern triggered by the Zika pandemic (http://www.who.int/neglected_diseases/news/mosquito_vector_control_response/en/).

The study reported here was a phase-2 evaluation of OX513A under natural environmental conditions in physically-contained large field cages. The strain was tested for its mating competitiveness and for its effectiveness in suppressing a local population of *Ae. aegypti*.

## Materials and Methods

### Physically-contained field cage facility

The physically-contained field cage facility, hereafter referred as "Contained Facility", was developed at the GBIT (Gangabishan Bhikulal Investment and Trading Limited) campus,

located in a rural area near Dawalwadi village, Jalna District, Maharashtra State, India (Fig 1).

**Fig 1. (a) Site of physically-contained facility (Map data: Google, 2018 CNES/Airbus), (b) physically-contained field cage facility, (c) field cages, (d) arrows indicate larval container covered with mesh and sucrose feeding rods, (e) arrows indicate refuges for mosquitoes, (f) ant traps surrounding the supporting pillars, and rodent traps, (g) arrows indicate sucrose feeding cotton pads, and a black plastic container, holding an earthen pot containing water and covered with mesh cloth as a refuge ~~area~~.**

The site was isolated from human habitation by more than 400m and is 1 km west of Dawalwadi village (19°51'53.68"N, 75°47'30.80"E). There are typically 3 seasons during the year: the summer, between March and June, with limited rainfall and maximum temperatures up to 45°C; the wet (monsoon) season between July and September with average rainfall between 650 to 750 mm, and the winter season, between October and February, with minimum temperatures between 9 to 10°C and maximum temperatures around 30 to 31°C. The contained facility was designed following the methods reported by Facchinelli et al. [18-19] with slight modifications (Fig 2).

**Fig 2. Physically-contained field cage facility design and specifications.**

The contained facility consisted of six field cages fastened on a wooden platform of 580 $m^2$ (29 m × 20 m) elevated 1m above the ground on steel support poles. The footings of the supporting poles had ant traps containing detergent water. The ground below the platform was covered by a thick plastic sheet to prevent grass and other plants growing up to the platform. Six stitched cages of 8×8×3 m (L×W×H) size, constructed of white tricot knitted polyester woven UV (+40) treated mesh cloth (108 × 33 tpi) were supported over metal frames. Within each of these cages, experimental cages were erected by tying the nets to the metal frame supporting the outer cage with manila ropes at the sides, thereby creating a double layer of containment. The experimental inner cages were 6×6×2 m (L×W×H) stitched

of white tricot mesh cloth (mesh count of 625 per square inch) partitioned into halves, providing paired cages with each half measuring 6×3×2 m (L×W×H), and the cages were reinforced at the angles and seams with white canvas. Each inner paired cage had vestibules at the opposite ends measuring 1.5m×1.5m (except one cage unit which was used as a field laboratory) with zippered opening to access into cages through the sleeves in the vestibules. Lockable zippered openings were installed for each cage on the opposite side of the vestibules for direct access into the internal space of cages when required. For all paired five cage units with vestibules, one half served as an untreated control and the other half as a treatment cage. The cage bases were fixed to the wooden platform by aluminum strips and screws to completely contain the cages from the outer environment. One cage without vestibule was used as a field laboratory for handling of biological materials. The outer cages served as double containment to ensure no escapees by providing a buffer zone of 1m around the experimental cages, into which two BG Sentinel® traps (Biogents) holding BG lure cartridge as attractant were placed to capture any escaped mosquitoes, the attractant were replaced at 16 to 20 weeks interval and electric mosquito bats provided to manually kill mosquitoes as required.

The height of the roof at the center from platform was 6.5m, made of translucent corrugated plastic sheets to provide shade and to protect the cages from rain and exposure to direct sunlight. White color floor mats were affixed on the whole platform. The whole physically-contained structure was protected by a chain link fencing on all sides having a lockable door and a single entry point halfway from the platform to protect from any unauthorized entry or animal intrusion. The top half of the structure was also covered on all sides with green shade net (50%) to provide additional UV protection to the cages.

Data loggers (HTC RH –Temp Data Logger Easy Log) were placed in each section of the cage, and one placed outside the cage in the contained facility for recording temperature and humidity. One small potted plastic plant, a stack of clay bricks, and clay pots were placed

in each of the paired experimental cages as a refuge for mosquitoes (Fig 1). Each cage has a steel rack affixed inside the cage near the vestibule, that could be accessed through the opening sleeves. A black plastic container with a clay pot containing water and covered by mesh was placed inside each cage on the rack as a refuge and to create a humid atmosphere for mosquitoes. Ovitraps were placed outside surrounding the whole unit for detection of potential escapees from the field cages. Rodent traps were placed at the bottom of the platform to ensure safety of the contained facility. Rodent traps used were lockable tamper-proof Rodabox™ baiting containing glue board (Trubble Gum®) for trapping. ~~The rodent gets trapped on the board and can then be disposed.~~

**~~Regulatory~~ approval**

All experiments were performed in accordance with the approved experimental design and protocols received from the Review Committee for Genetic Manipulation, Department of Biotechnology, Government of India (Letter No.BT/BS/17/328/2008-PID/Vol.2 dated; 15 Feb 2017).

**Mosquito strains**

**OX513A *Aedes aegypti* strain.** Eggs of OX513A were imported from Oxitec, UK in accordance with the import permit (No.BT/BS/17/328/2008-PID) issued by the Department of Biotechnology (DBT), Government of India, New Delhi in November 2011 and the culture was maintained at an Arthropod Containment Level II (ACL II) laboratory facility [20], Dawalwadi, Jalna District, Maharashtra State.

***Aedes aegypti* wild type strain.** Aquatic stages and eggs of *Ae. aegypti* were collected locally from Aurangabad during 2011 to establish the culture. Since then, the strain (hereafter referred as AWD) has been maintained under ACL II laboratory conditions.

**Mosquito Rearing.** OX513A was maintained under ACL II laboratory conditions with a temperature of 27±2°C and relative humidity (RH) of 70-80%.

OX513A eggs were hatched under reduced atmospheric pressure for synchronized hatching and reared using tap water supplemented with 30 µg/ml tetracycline (chlortetracycline hydrochloride, 75%, Sigma), an antidote used for repressing the expression of the self-limiting gene. The larvae were provided with 2-3 drops of Liquifry (Interpet, UK) for the first day of development and later fed with ground TetraMin® fish food (Tetra Spectrum brands, USA) during the rest of the developmental period until pupation, under a standardized feeding schedule. Male and female pupae were sexed by eye based on the size difference and introduced into rearing cages of 30cm x 30cm x 30cm at 1:2 male/female ratio, prior to adult emergence. The emerged adults were subsequently provided with 10% sucrose solution and the female adults were fed with goat blood using a membrane feeding technique twice per week [21]. Eggs were collected once per week using water containers lined with seed germination paper – a substrate preferred by gravid females. Following collection, the papers were allowed to dry for 2 days before storing under laboratory conditions.

AWD *Ae. aegypti* (wild-type) were reared as indicated above for OX513A, except for the addition of chlortetracycline to the rearing water.

**Experiment 1**

**Mating competitiveness experiment.** Mating competitiveness experiments were performed to evaluate the performance of OX513A strain male adults versus male adults of AWD strain in the field cages.

Experiments were conducted as three replicates during the calendar month of March by introducing adult mosquitoes in a ratio of 100:100:100 (OX513A♂: AWD ♀: AWD ♂) into the cage. Experiments were initiated by first introducing virgin 3-4 day old male adult mosquitoes of both OX513A/AWD strains and allowing them to acclimatize for one hour. This was followed by the introduction of 100 female adult mosquitoes (AWD strain only). After 24 hours female adults were fed with animal (goat) blood. Adult females were collected

post blood meal using a standard manual aspirator ~~and transferred to the ACL-II laboratory~~. When entering the cages, operators used protective clothing and full cover protective mask (Mutex® Light+). The duration for female adult collection and the number of adults collected in each cage were recorded. Collected female adults from individual cages were transferred to laboratory mosquito rearing cages (30×30×30cm) and were fed with a second blood meal. Female mosquitoes were then transferred into individual mesh screened oviposition boxes [6.5 cm (Ø) and 8.0 cm (ht)], each provided with a container of water [2.7 cm (Ø) and 3.0 cm (ht)] lined with filter paper for oviposition. Eggs collected from each individual female were hatched and reared separately in tetracycline containing water, and screened during third instar larval stage for the presence of DsRed2 fluorescence (Fig 3) using a fluorescence microscope (Leica MZ10F stereomicroscope with fluorescence filter set ET-DsRed composed with an excitation filter model ET545/30x center wavelength at 545nm with 30nm bandwidth and emission filter center ET620/60m at 620nm with 60nm bandwidth). Presence of fluorescence indicated that the offspring were fathered by OX513A males, and absence of fluorescence indicated offspring fathered by AWD wild type males. Presence of both fluorescent and non-fluorescent larvae indicated double mating by female adults with both AWD wild type and OX513A male adults.

**Fig 3. Fluorescence imaging of OX513A *Aedes aegypti* strain larvae in comparison with AWD strain *Aedes aegypti*. OX513A larvae display bright punctate fluorescence throughout the larva, which is distinct from auto fluorescence from the larval gut that is also observed in the AWD larvae.**

**Pupal Size.** The size of male pupae from the experimental rearing lot was determined by measurement of the dorsal side of the thoracic region (Fig 4). Pupae were transferred to a small petri dish holding cold water to immobilize the pupae and a measuring scale along the side. Images were captured manually using a 14 mega pixel camera and the dorsal thoracic region measured using “ImageJ” software to determine the pupal size.

**Fig 4. Measurement of *Ae. aegypti* pupal width on the dorsal side of cephalothoracic region.**

**Wing Measurement.** Right wing length was used as an indicator of adult size. Adult mosquitoes emerged from the experimental rearing lot were preserved in 90% alcohol for dissecting of the right wing for wing measurements. Right wings were removed under a dissection microscope and mounted on slide in leptocarphin and sealed with transparent nail polish. The images of the mounted wings were taken alongside a measurement scale. The images were analyzed in Image-J software and measurements of the wing taken from the alular notch to the apex margin of the wing, excluding the fringe (Fig 5).

**Fig 5. Right wing measurement of *Ae. aegypti* male adults of OX513A and AWD strains.**

**Experiment 2**

**Population suppression experiment.** The population suppression experiment consisted of two stages; (1) Establishment of a stable population of wild type AWD strain and (2) Suppression of wild type AWD population by frequent releases of OX513A male adults in treatment cages.

**Population stabilization.** The method for stabilization of AWD population was adapted from that reported by Wise de Valdez et al. [22].

Initially all 10 cages were populated by the introduction of 200 male and 200 female adults (AWD strain) and provided with 10% sucrose. Female mosquitoes were fed with goat blood twice a week using feeding membranes. Following the blood feed, black plastic containers containing water and lined with a paper strip were added to collect eggs. Eggs laid by females were collected weekly, dried, counted and hatched under ACL II laboratory conditions. The hatched larvae were reared and returned to their respective cages as second-instar larvae ($L_2$) at a return rate of 200/week. Only during the initial period of population establishment and to ensure synchronized introduction rates in all cages, the number of larvae introduced (i.e. 200 larvae) was supplemented, if required, with larvae from the laboratory

AWD colony. Weekly egg counts were used to determine establishment of stable populations.

To monitor the population dynamics, adults were sampled weekly using BG Sentinel traps (BioGents) placed in each cage for 60 minutes and the adult males and females captured were counted and returned to their respective cage. Mortality, if any, during adult monitoring was noted. Weekly monitoring of the population using BG sentinel traps was conducted throughout the experimental period (i.e. population stabilization and population suppression period).

**Population suppression.** In all the control cages, the weekly replenishment rate of 200 $L_2$ larvae from the eggs collected weekly in the respective cages was held constant throughout the experimental period.

In treatment cages following the population stabilization, in addition to the weekly introduction of 200 $L_2$ larvae, OX513A adult male introduction was initiated. The released OX513A male adults were reared under laboratory conditions and were sex-sorted during the pupal stage. The sorted male pupae (100/container) were added to plastic containers containing water and covered with a mesh, and allowed to emerge**.** Emerged adults were visually inspected and female adults (if any) were removed using an aspirator. Male cohorts for release were transferred to the contained facility for releasing at a ratio of 10:1 (OX513A males : AWD males) in each treatment cage (i.e. 1000 male adults/treatment cage/week), approximately 10 times the weekly return rate of 200 second instar AWD larvae/week, assuming male/female emergence ratio of 1:1 (approximately 100 AWD males). The release number of OX513A male adults was constant throughout the experimental duration.

After initiating the releases of OX513A male adults, $L_2$ larvae equivalent to 10% (if egg count was >2000) of the egg yield or 200 $L_2$ larvae (if egg count is <2000) and or all the larvae hatched (if egg count was <200) in each cage were screened for the presence of DsRed fluorescence. Once fluorescent larvae were observed, thereby confirming mating with

OX513A strain male adults, the number of larvae replenished back to respective treatment cage was proportionally adjusted to reflect the egg yield and larval introduction rate (i.e. 200 larvae) of respective paired control cage [22]. For example, if a control cage produces 1,000 eggs one week with constant return rate of 200 larvae per week (1/5), and a treatment cage produced 2000 eggs in the same week than a similar proportion of the total number of eggs (1/5) is returned as $L_2$ larvae to the cage (i.e. 400 larvae). If a treated cage in the same week produces 500 eggs then 100 larvae will be returned to the cage.

$$\text{Larval return rate in treatment cage} = \frac{\text{Number of eggs in treatment cages } \times \text{Number of larval return rate in control cage}}{\text{Number of eggs in control cage}}$$

As discussed in previously published literature [22], if 200 larvae are returned constantly to each cage in both control and treated cages, the return rate will ~~artificially~~ increase the population in the treated cages if the egg density is low, or artificially decrease it if the egg density is high.

**Wing measurement.** During weeks 1-3 following release initiation, 25 emerged adults from each OX513A male adult release cohort and 25 male adults from each control cage were collected to determine and compare the body size based on wing measurement.

**Biosafety.** Transport of all insect life stages during the experimental period between the laboratory and contained facility was within escape-proof, sealed containers/bags. All waste materials including mosquitoes were autoclaved before disposal.

**Statistical Analysis**

**Mating competitiveness.** The t-test was used to compare the numbers of females mated with OX513A, AWD males and double-mated females (both with OX513A and AWD strain male adults). Chi-square tests were applied to test the observed mating index against the expected mating index. Relative mating index was calculated as the number of females mating OX513A males/total number of females mated [23].

**Population suppression experiment.** Statistical comparisons of two independent groups, such as wing size between OX513A and AWD, egg production and egg hatch between treatment and control cages, were performed using the independent t-test.

The distributions of weekly eggs produced in cages were assessed for normality using the Kolmogorov-Smirnov (K-S) test. Weekly variation of egg production in treatment and control cages were analyzed by one-way analysis of variance (ANOVA) followed by post-hoc Tukey's b test.

To compare the egg production between paired treated and control cages during the suppression phase, the data was normalized first by converting it to square root and analyzed using one-way analysis of covariance (ANCOVA). Any effect of OX513A releases in treatment cages was detected by comparing weekly egg production with the respective paired control cage.

The weekly change in the numbers of male and female adults captured by the BG Sentinel traps in the paired control and treated cages was analyzed using the non-parametric Mann-Whitney U test. The aim was to determine whether the number of males and females differ significantly between the paired control and treatment cages before and after the addition of OX513A males. In addition, for the graphical representation of the adult variation in each cage, the values were log-transformed.

To determine whether the environmental factors (temperature and humidity) ~~have~~ influenced the mosquitoes' egg production, a correlation analysis (Pearson correlation coefficient) of the weekly average temperature/humidity (maximum and minimum) and egg production in control and treatment cages before and after OX513A releases was completed. The weekly maximum and minimum averages for temperature and humidity were calculated from the daily maximum and minimum temperatures recorded between Wednesday to Tuesday in each respective cage (the day range was determined on the ovitrap retrieval day - Tuesday).

Pearson correlation coefficients were also calculated for the relation between the number of fluorescent larvae and the egg production in each of the treatment cages as well as between the number of fluorescent larvae and the larval return, to indicate whether and how the variables tested influenced each other.

Statistical analyses of the data were conducted using statistical software SPSS version 20 (IBM Corporation, New York, USA).

# Results

## Mating competitiveness

In order to assess mating competitiveness of the transgenic OX513A strain, male adults were released in equal proportion with wild AWD adult males to compete for an equal number of AWD female adults over a period of 24 hours. Measurements of the pupal size and right wing of adults indicated non-significant difference between the two strains, with a mean pupal size of 0.981±0.01 mm and 0.977±0.012 mm (p=0.789, two tailed independent T test) and mean right wing length of 2.04±0.01 mm and 2.02±0.02 mm (p=0.447, two tailed independent T test) for OX513A and AWD males respectively (Supplementary S1 and S2 Tables).

Twenty-four hours after the release, out of 79.7% of the female adults recaptured, 60.3% females laid eggs with an average of 58 eggs per female adult (Supplementary S3 Table). Eggs laid by individual females showed a hatching rate of 76.6%.

Fluorescence screening revealed that 41% and 48% of the AWD female adults mated with OX513A males and AWD males respectively, while 10.9% of the AWD adult females mated with both OX513A and AWD males (Fig 6).

**Fig 6. Percent mating of *Aedes aegypti* female adults with male adults of OX513A/wild type AWD *Aedes aegypti* strain and error bar representing standard error.**

Chi-square analysis (Supplementary S4 Table) of the observed mating proportions revealed non-significant deviation from the expected mating proportion of 1:1:0 (AWD

males: OX513A males: double mating) ($X^2$=2.886; p = 0.2362). In addition, the paternal origin did not impact on the number of eggs laid by the wild females (Table 1).

**Table 1. Average number of eggs laid by AWD females mated with OX513A males, AWD males and those double-mated.**

| | AWD female adults mated by | | |
|---|---|---|---|
| | AWD strain ♂ | OX513A Strain ♂ | Both [AWD & OX513A strain] ♂ |
| Number of eggs laid/female (Mean±SE) | 58.5±1.97[a] (n= 4914) | 60.6±1.85[a] (n=4360) | 59.7±3.97[a] (n=1134) |
| 95% confidence interval | 54.6 - 62.4 | 56.9 - 64.2 | 51.3 - 68.0 |
| One-way ANOVA | df = 2; F = 0.284; p>0.05 | | |

Figures within the rows indicated with same alphabets show non-significant difference between the values by one-way ANOVA following Tukey's-b test.

**Population suppression experiment**

The suppression experiment was initiated on 13th of April 2017 and terminated on 12 December 2017 after 34 weeks. Experiments were performed in five paired cage units (Unit A to E) with a control and treatment cage in each unit. Each paired unit had the control and treatment cages randomly assigned - cages 1, 4, 5, 7, and 10 were controls while 2, 3, 6, 8 and 9 were treatment cages.

Establishment of a stable population of wild AWD strain – defined as stable egg output, was achieved in 14 weeks in all the paired cage units (Fig 7). Number of eggs produced in each cage was analyzed by one-way ANOVA, which indicated a significant increase of the mosquito population from week-8 while egg production was stabilized by week-14 (Supplementary S5 Table).

**Fig 7. Weekly egg production in control and treatment cages during experiment. Egg production number increased from week-9 reflecting establishment and stable adult mosquito population during subsequent weeks in all cages. Post establishment, OX513A**

**male adult releases weekly were initiated (weekly-15 onwards) (vertical dashed line) in the all 5 treatment cages.**

The K-S test for normality of the distribution on weekly egg production showed non-significant difference between cages indicating a normal egg production rate during the population stabilization period from week 1-14 ($p > 0.05$). One exception was week-4 where the egg production varied significantly ($p < 0.05$) between the cages (Supplementary S5 Table).

Analysis of the weekly mean egg production during the stabilization period showed no significant differences between paired control and treatment cages (Sig 2-tailed >0.05). Egg production during week 13 and 14 increased significantly in control and treatment cages compared to the previous weeks but mean egg production for weeks 13 and 14 was not significantly different between the control and treatment cages indicating stabilization of the AWD population (Supplementary S5 Table).

Across the whole stabilization period (14 weeks) the weekly egg production in paired cages did not vary significantly (Sig. 2-tailed >0.05) indicating no bias of the cage position on the mosquitoes' performance (Fig 8).

**Fig 8. Average egg productive in paired cage units per week in control and treatment cages during stabilization period (week 1-14) with error bar representing standard error.**

Stabilization of the AWD population in cages was followed from week 15 by weekly introduction of 1000 OX513A male adults in each treatment cage, corresponding to a release ratio of 10:1 (OX513A males: AWD males), based on the weekly introductory rate of $L_2$ larvae per cage (i.e. 100 males: 100 females). OX513A male releases in treatment cages were terminated when no egg production was observed consecutively for a period of five weeks.

Following the weekly introduction of OX513A male adults in treatment cages, the eggs collected weekly were hatched and screened for presence of DsRed2 fluorescence.

Calculations of weekly larval return rate were initiated for the treatment cages following the identification of the first fluorescent larvae (Fig 9).

**Fig 9. Weekly larval return rate in control and treatment cages before and after OX513A male adult releases and fluorescence observation. Post OX513A male adult release initiation in treatment cages and following first observation of introgression of OX513A based on fluorescence screening of progeny, the larval return rate was calculated for the treatment cage in proportion to paired control cage.**

Weekly larval return rate in treatment cages for cage 2 and 3 was high during the first two weeks (i.e. week 15 and 16) post OX513A release initiation, corresponding to the egg production rate and gradually decreased in the subsequent weeks. In cage 6, 8 and 9 the larval return rate decreased proportionately with the decrease in egg production followed by the increase in fluorescence percent (Fig 9 and Supplementary S1 Fig). Mortality during the development stages following weekly introduction of larvae ($L_2$) into the cages was observed from week 18 onwards. Mortality in the treatment cages observed during the week 18 to week 24 fluctuated between 46.8% and 59.4% and reached 100% by week 28 and 29 (Supplementary S2 Fig).

Analysis of egg hatching rate in control and treatment cages during pre- and post-release period showed non-significant differences ($p>0.05$) (Fig 10).

**Fig 10. Hatching percent of eggs produced in control and treatment cages during the pre-release and post release period of OX513A male adults in treatment cages. The analysis indicates no significant difference between the Control and Treatment cages ($p>0.05$). Error bar represents standard error.**

Correlation analysis of larval fluorescence against egg production (Table 2) was found to be significant and negatively correlated in treatment cage 2, 3 and 6 and non-significant in cages 8 and 9 (Sig. 2-tailed <0.05). The correlation analysis between the larval

fluorescence and larval return rate found significant and negative correlation in cages 2, 3, 6 and 9 (Sig. 2-tailed<0.05).

**Table 2. Correlation between fluorescence vs egg production and fluorescence vs larval return rate post initiation of OX513A male adult introduction in treatment cages from week 15.**

| Correlation | Treatment cages | | | | |
|---|---|---|---|---|---|
| | Cage 2 | Cage 3 | Cage 6 | Cage 8 | Cage 9 |
| Fluorescence *Vs* Egg production | | | | | |
| Pearson Correlation (r value) | *-0.543 | *-0.646 | *-0.769 | -0.455 | -0.311 |
| Sig. (2-tailed) | 0.036 | 0.017 | 0.016 | 0.118 | 0.26 |
| Fluorescence *Vs* Larval return rate | | | | | |
| Pearson Correlation (r value) | **-0.685 | *-0.554 | **-0.947 | -0.298 | *-0.583 |
| Sig. (2-tailed) | 0.005 | 0.05 | 0.0001 | 0.323 | 0.023 |

*Correlation (negative) is significant at 0.05 (2-tailed).
**Correlation (negative) is significant at 0.01 (2-tailed).

Following the initiation of OX513A male releases in treatment cages, average egg production decreased significantly (sig 2-tailed = 0.005) from week-20 (Fig 7 and Supplementary S6 Table), while for cage-6 the decrease in egg production was visible from week-17. Egg production analysis by ANCOVA in paired cage units A, B, D and E was significant (p<0.05) except cage unit C, indicating significant reduction in egg production in respective treatment cages post initiation of OX513A male adult releases (Fig 11). In cage-6 egg production significantly decreased and no egg production was observed post 9 weeks of OX513A releases i.e., from 24$^{th}$ week and no further egg production was observed subsequent for 4 weeks till the 28$^{th}$ week from the start of the experiment.

**Fig 11. Egg production of *Aedes aegypti* AWD strain mosquito in paired cage (control versus treatment) units (A-E) during suppression period (OX513A male adult releases in treatment cages) from week-15 and ANCOVA analysis.**

The AWD population was considered eliminated when no egg production was recorded for a period of 5 consecutive weeks in the treated cages. The first treatment cage with no egg production was Cage 6 in week 24, followed by Cages 3 and 8 by week 28 and Cages 3 and 9 by week 30. In all control cages, the AWD egg production continued until they too were terminated after 5 weeks of no egg production in the paired treated cage (i.e. the control cages were terminated 5 weeks after the production ceased in the paired treated cage) (Fig 7).

At the end of the experiment the sucrose feeders were removed for a period of 10 days to ensure all mosquitoes were dead, the cages were cleaned and the biological waste was autoclaved for disposal.

Adult sampling using BG Sentinel traps was performed weekly in all the cages from week 5 to determine the variation in male and female numbers. Following the initiation of OX513A releases (week 15), the number of adult males captured in the treatment cages significantly increased compared to control cages (Fig 12), ~~as expected.~~

**Fig 12. Male adult sampling using BG sentinel traps in control and treatment cages (weeks 5 to 33).**

The number of adult females captured in the treated cages decreased significantly compared to the control paired cages starting from week 23 (cage 6) and week 27 (cages 2, 3, 8 and 9). No female mosquitoes were captured in treated cages from weeks 30 (cage 2), 29 (cage 3), 24 (cage 6), 30 (cage 8) and 31 (cage 9) (Fig 13).

**Fig 13. Female adult sampling using BG sentinel traps in control and treatment cages (week 5 to 33).**

Statistical analysis of total males and females captured in paired cages has indicated that before releases the males and females number captured was not significantly different

between the paired treated and control cages (Supplementary S7 Table). However, during the release period, both the total number of males and females captured was significantly ($p<0.05$) different between the paired control and treated cages (except for Unit B which narrowly missed the significance threshold – $p=0.06$) (Supplementary S8 Table).

To determine whether the environmental factors had an impact on the mosquito egg production, the temperature and humidity were recorded hourly in all the cages throughout the experimental period, using dataloggers. The conditions in the treated and control cages were found to be similar for both temperature and humidity (Supplementary S3 Fig). The correlation analysis between the egg production and the temperature and humidity variation revealed a number of statistically significant positive and negative correlations, however, none that would provide support to a decrease in egg production in the treated cages due to environmental conditions (Supplementary S9 Table).

## Discussion

Earlier laboratory assessments of OX513A strain for mating competitiveness and self-limiting gene expression have shown the OX513A strain to be effective and equally fit in comparison with wild type *Ae. aegypti* mosquitoes [24].

Testing under confined conditions that limit the release of the organisms into the environment forms part of the phased testing approach recommended by some regulatory authorities to assess the safety and efficacy of novel technologies [25]. A lack of fitness could potentially undermine performance by reducing the competitiveness with wild type males under open field conditions. Consequently, for developmental and deployment of such biological approaches it is useful to define and test fitness, to help predict the likelihood of success of an intervention strategy [26]. In contrast, another topic that arose is the fitness of the AWD wild type strain used to populate the cages for demonstrating population suppression. The cyclic colony of the AWD strain was maintained under laboratory

conditions for >6 years, a lack of fitness of the wild type strain used for the study could possibly undermine the outcome of the experiment. In this context, our studies on mitochondrial cytochrome oxidase I (COI) region of laboratory strains including AWD strain and field collected wild type strains have shown genetic similarity with field collected wild type strains and Indian/worldwide isolates [27]. In addition, laboratory studies on fitness parameters support the AWD strain to be an ideal wild type strain for cage studies [24]. In the present study, the OX513A strain of *Ae. aegypti* was tested for mating competitiveness and suppression of laboratory maintained wild type AWD *Ae. aegypti* strain in large field cages under natural environmental conditions.

~~The mating experiments evaluated the mating competitiveness of OX513A male adults against wild type AWD strain male adults.~~ The sizes of OX513A males and AWD males under test conditions were analyzed by comparing the size of the pupae during development and wing length for emerged adult size. Some reports suggest that size of the mosquitoes is associated with fitness and reproductivity, and that larger mosquitoes could be at an advantage over smaller ones [28-30]. Measurements of OX513A and AWD pupae and adults for comparisons of size indicated no significant difference between the two strains. Results of the mating experiments indicated OX513A male adults to be equally fit in comparison with the wild AWD strain, with 41.1 % of the females being mated by OX513A males compared to 48.0% mated by AWD males. Although a small percent of females (10.9%) were double-mated, the chi-squared test showed that the deviation from relative mating proportion (observed versus expected - 1:1:0, OX513A mated: AWD mated: double mated) was non-significant. The calculated expected proportion for double mating was considered to be nil based on the fact that the *Ae. aegypti* are monoandrous and typically mate once in their life span, and are commonly refractory towards a second mating, which is a rare occurrence in natural environments (up to 14%) [31-32]. In an earlier laboratory study, Massonnet-Bruneel et al. (2013) [33] found that the OX513A males are as efficient as wild-

type males at inducing refractoriness to re-mating in wild-type females and the low incidence of double-mating found in this study confirm these earlier findings. In this context, it is important to emphasize that double mating observed in the field cage study may be attributable to the restriction of movement for mated female adults in the cage which could have led to frequent encounters with the males of both the strains leading to second mating.

The proportion of mating success of OX513A strain male adults and AWD strain male adults with respect to total eggs laid by the females was found to be similar, with no significant differences between the females mated by OX513A males, AWD males or the double-mated ones.

Short-term laboratory competition studies have been shown to underestimate the fitness costs of the strains analyzed and that a better way to assess the mating competitiveness before field release is through large caged experiments under field conditions [19]. The results of the present study indicate that the transgenic OX513A males have similar mating competitiveness with the AWD males and that the AWD females do not preferentially discriminate between the two strains.

~~For the population suppression experiment, a stable AWD population was established in all the experimental cages over a period of 14 weeks. During this population establishment/stabilization period, egg production was found to be variable in the early weeks but increased and stabilized post week 8, and by weeks 13 and 14 reached an average of 5000 eggs/cage, indicating stability and ideal mosquito population size in all the cages. The stabilization point for the mosquito population (weekly egg output) in the cages was calculated based on the observation from the mating competitiveness experiment and weekly larval return regimen.~~ Week 14, at the end of July 2017, with its favorable climatic conditions (temperature/humidity) for mosquito breeding, was considered ideal for initiation of OX513A male releases in the treatment cages. Previous large cage studies using *Aedes aegypti*

populations have also used pre-release periods of up to 20 weeks with the mosquito population showing stability in egg output after 10 weeks of maintenance [19, 34].

During the pre-treatment phase of the study, the number and ratio of adult males and females in the cages did not differ significantly (Mann Whitney test) indicating a similar reproductivity output. After 14 weeks of stabilization of the caged population, the OX513A male adult releases were initiated. Previous lab studies [35], cage studies [19] and open field releases [12-15] have used a 10:1 ratio (OX513A males: AWD males) that was shown to favor population extinction. Here we used a similar over flooding ratio with a weekly release of 1000 OX513A adult males and the return of 200 larvae. Following the detection of fluorescent larvae and the decrease in overall cage reproductive output, the number of larvae returned represented 10% of the total number of eggs produced in a week. This strategy was previously used in other studies [22] to avoid the artificial increase or decrease of a population by using a constant larval return.

Weekly adult sampling of male adults during the OX513A release period confirmed the significant increase in the number of adult males compared to the control cages throughout the duration of the suppression phase. The analysis of the number of females trapped indicated that after week 20, their number started to decrease and by week 24, in cage 6 there were no AWD individuals. Cages 3 and 8 achieved complete suppression by week 28 while 2 and 9 by week 30. These results are consistent with the progression of the fluorescent proportion in the population, which started at 2.5% in the week 15 (for cage 2, 6 and 10) and reached 100% by week 22 (cage 6), 27 (cage 3 and 8) and 28 (cage 2 and 9). In effect, the negative correlation identified between the fluorescence level and egg production supports the observation that the release of OX513A males has led to a gradual reduction of AWD female numbers.

The reduction in the number of AWD females impacted on the production of eggs which followed a similar decreasing pattern after the initiation of OX513A releases: by week

19 the number of eggs has decreased significantly in the treated cages while by week 22 the difference was significant.

Our experimental findings clearly demonstrate that OX513A strain is competitive and fit under natural environmental conditions in India, indicating its potential to produce the desired effect under open field environment and suppress natural populations of *Aedes aegypti* in India effectively. OX513A has previously been shown to be effective in suppressing wild populations of *Aedes aegypti* in Brazil, the Cayman Islands and Panama [13,15,36-37].

## Conclusion

The present study demonstrated that OX513A strain has a similar mating competitiveness with the mosquito population collected locally and that it is an effective tool for the control of the wild population of *Aedes aegypti* by completely suppressing the wild populations in the 5 replicated cages within 10 to 16 weeks of releases. As the experiments were conducted in open environment under natural conditions it can be assumed that when released in the environment the OX513A mosquitoes will perform similarly, and be capable of suppressing local *Aedes aegypti* populations.

The study was conducted following the containment measures agreed by the national Review Committee for Genetic Manipulation (RCGM), the competent authority under the Department of Biotechnology that monitors the safety related aspects of the projects involving genetically modified organisms. Additionally, representatives from five nearby villages, together with government representatives from State and District Health Departments, and the District Malaria Officer, were invited to the experimental site to disseminate information on the ongoing activities and raise awareness of this technology. Transparency and public involvement opportunities are essential for gaining the confidence

of the local communities and provide a sustainable implementation of the technology in the field in the future.

## Acknowledgements

The authors would like to specially thank Institutional Biosafety committee (IBSC) members of GBIT, Dr. L. S. Shashidhara, Dr. Ananth Pandhare, Rushikesh Naigaonkar, Dr Girish Rao, and Dr K V Seshu Reddy for their suggestions and productive views. The authors would like to thank Oxitec colleagues for their constructive discussion and suggestions throughout.

## References

1. CBD Secretariat. Cartagena protocol on biosafety to the convention on biological diversity. Secretariat of the Convention on Biological Diversity, Montreal, Canada; 2000.
2. Waltz E. GM mosquitoes fire first salvo against Zika virus. Nat Biotechnol. 2016; 34(3): 221-222. Doi: 2.10.1038/nbt0316-221
3. Stanaway JD, Shepard DS, Undurraga EA, Halasa YA, Coffeng LE, Brady OJ, et al. The global burden of dengue: an analysis from the global burden of disease study 2013. Lancet Infect Dis. 2016; 16(6): 712-723. doi: 10.1016/S1473-3099(16)00026-8
4. NVBDCP. National Vector Borne Disease Control Programme, Directorate General of Health Services, Ministry of Health and Family Welfare, Government of India. http://www.nvbdcp.gov.in/index.php.
5. Shepard DS, Halasa YA, TyagiBK, Adhish SV, Nandan D, Karthiga KS, et al. Economic and disease burden of dengue illness in India. Am J Trop Med Hyg. 2014; 91(6): 1235–1242. doi: 10.4269/ajtmh.14-0002
6. Swaminathan S, Khanna N. Dengue vaccine development: Global and Indian scenarios. Int J Infect Dis. 2019; 84S: S80–S86. doi: 10.1016/j.ijid.2019.01.029

7. Martina BE, Koraka P, Osterhaus AD. Dengue virus pathogenesis: an integrated view. Clin Microbiol Rev. 2009; 22(4): 564–581. doi: 10.1128/CMR.00035-09
8. World Health Organization. Global strategy for dengue prevention and control 2012-2020. Geneva: WHO Press; 2012.
9. Focks DA, Daniels E, Haile DG, Keesling JE. A simulation model of the epidemiology of urban dengue fever: literature analysis, model development, preliminary validation, and samples of simulation results. Am J Trop Med Hyg. 1995; 53 (5): 489-506. doi: 10.4269/ajtmh.1995.53.489
10. Focks DA. A review of entomological sampling methods and indicators for dengue vectors. WHO, Geneva; 2003.
11. Bharati M, Saha D. Multiple insecticide resistance mechanisms in primary dengue vector, *Aedes aegypti* (Linn.) from dengue endemic districts of sub Himalayan West Bengal, India. PLoS One 2018; 13(9): e0203207. doi: 10.1371/journal.pone.0203207
12. Harris AF, Nimmo D, McKemey AR, Kelly N, Scaife S, Donnelly CA, et al. Field performance of engineered male mosquitoes. Nat Biotechnol. 2011; 29(11): 1034-1038. doi: 10.1038/nbt.2019
13. Harris AF, Mckemey AR, Nimmo D, Cutis Z, Black I, Morgan SA, et al. Successful suppression of a field mosquito population by sustained release of engineered male mosquitoes. Nat Biotechnol. 2012; 30(9): 828-830. doi: 10.1038/nbt.2350
14. Lacroix R. Mckemey AR, Raduan N, Wee LK, Ming WH, Ney TG, et al. Open field release of genetically engineered sterile male *Aedes aegypti* in Malaysia. PLoS One 2012; 7(8): 1-9. doi: 10.1371/journal.pone.0042771
15. Carvalho DO, Mckemey AR, Garziera L, Lacroix R, Donnelly CA, Alphey L. Suppression of a field population of *Aedes aegypti* in Brazil by sustained release of transgenic male mosquitoes. PLoS Neg Trop Dis. 2015; 9(7): e0003864. doi: 10.1371/journal.pntd.0003864

16. Phuc HK, Andreasen MH, Burton RS, Vass C, Epton MJ, Pape G, et al. Late-acting dominant lethal genetic systems and mosquito control. BMC Biology. 2007; 5: 1-11. doi: 10.1186/1741-7007-5-11
17. Patil PB, Gorman KJ, Dasgupta SK, Seshu Reddy KV, Barwale SR, Zehr UB. Self-limiting OX513A *Aedes aegypti* demonstrate full susceptibility to currently used insecticidal chemistries as compared to Indian wild-type *Aedes aegypti*. Psyche- J Entomol. 2018; Vol 2018. https://doi.org/10.1155/2018/7814643
18. Facchinelli L, Valerio L, Bond JG, Wise de Valdez MR, Harrington LC, Ramsey JM, et al. Development of a semi-field system for contained field trials with *Aedes aegypti* in Southern Mexico. Am J Trop Med Hyg. 2011; 85(2): 248-256. doi: 10.4269/ajtmh.2011.10-0426
19. Facchinelli L, Valerio L, Ramsey JM, Gould F, Walsh RK, Bond G, et al. Field cage studies and progressive evaluation of genetically-engineered mosquitoes. PLoS Neg Trop Dis. 2013; 7(1): 1-12. Doi: 10.1371/journal.pntd.0002001
20. Arthropod containment levels (ACLs). Vector Borne and Zoonotic Diseases. 3: 75-90 2003.
21. Carvalho DO, Nimmo D, Naish N, Mckemey AR, Gray P, Wilke ABB, et al. Mass production of genetically modified *Aedes aegypti* for field releases in Brazil. J Vis Exp. 2014; 83(e3579): 1-10. doi: 10.3791/3579
22. Wise de Valdez MR, Suchman EL, Carlson JO, Black WC. A large-scale laboratory cage trial of the *Aedes Densonucleosis* Virus (AeDNV). J Med Entomol, 2010; 47: 392-399. doi: 10.1603/me09157
23. Li C, Marrelli MT, Yan G, Jacobs-Lorena M. Fitness of transgenic *Anopheles stephensi* mosquitoes expressing the SMI peptide under the control of a vitellogenin promoter. J Hered. 2008; 99(3): 275-282. doi: 10.1093/jhered/esn004

24. Patil PB, Niranjan Reddy BP, Gorman K, Seshu Reddy KV, Barwale SR, Zehr UB, et al. Mating competitiveness and life table comparisons between transgenic and Indian wild type *Aedes aegypti* L. Pest Manag Sci. 2015; 71: 957-965. doi: 10.1002/ps.3873
25. World Health Organization, Guidance framework for testing of genetically modified mosquitoes. 2013.
26. Scott TW, Rasgon JL, Black WCIV, Gould F. Fitness studies: developing a consensus methodology, in Bridging laboratory and field research for genetic control of disease vectors, ed. Knols BGJ & Frontis LC. Wageningen, The Netherlands, Ch. 16, 171-181, 2005.
27. Gandhi R, Yadav KK, Patil PB, Bihani P, Char B, Dasgupta SK, et al. Molecular analysis of mitochondrial cytochrome oxidase I gene of *Aedes aegypti* L. mosquitoes. J Asia Pac Entomol. 2020; 23: 51–59. https://doi.org/10.1016/j.aspen.2019.10.006
28. Ponlawat A, Harrington HC. Age and body size influence male sperm capacity of the dengue vector *Aedes aegypti* (Diptera Culicidae). J Med Entomol. 2007; 44(3): 422–426. doi: 10.1603/0022-2585(2007)44[422:aabsim]2.0.co;2
29. Ng'Habi KR, Huho BJ, Nkwengulila G, Killeen GF, Knols BGJ, Ferguson HM. Sexual selection in mosquito swarms: may the best man lose? Anim. Behav. 2008; 76(1): 105-112. https://doi.org/10.1016/j.anbehav.2008.01.014
30. Lang B. J.  Idugboe S, McManus K, Drury F, Qureshi A, Cator LJ. et al. The effect of larval diet on adult survival, swarming activity and copulation success in male *Aedes aegypti* (Diptera: Culicidae). J Med Entomol. 2018; 55(1): 29-35. doi: 10.1093/jme/tjx187
31. Gwadz RW, Craig GBJr. Female polygamy due to inadequate semen transfer in *Aedes aegypti*. Mosq News. 1970; 30: 355–360.
32. Helinski MEH, Valerio L, Facchinelli L, Scott TW, Ramsey J, Harrington LC. Evidence of polyandry for *Aedes aegypti* in semifield enclosures. Am J Trop Med Hyg. 2012; 86(4): 635–641. doi: 10.4269/ajtmh.2012.11-0225

33. Massonnet-Bruneel B.  Corre Catelin N, Lacroix R, Lees RS, Hoang KP, Nimmo D, et al. Fitness of a transgenic mosquito *Aedes aegypti* carrying a dominant lethal genetic system. PLoS One. 2013; 8(5): e62711. doi: 10.1371/journal.pone.0062711

34. Gato R. Lees RS, Bruzon RY, Companioni A, Menendez Z, González A, et al. Large indoor cage study of the suppression of stable *Aedes aegypti* populations by the release of thiotepa-sterilized males. Mem Inst Oswaldo Cruz. 2014; 109(3): 365-370. doi: 10.1590/0074-0276130519

35. Wise de Valdez MR, Nimmo D, Betz J, Gong H, James AA, Alphey L, et al. Genetic elimination of dengue vector mosquitoes. Proc Natl Acad Sci USA. 2011; 108(12): 4772–4775. doi: 10.1073/pnas.1019295108

36. Gorman K, Young J, Pineda L, Marquez R, Sosa N, Bernal D, et al. Short-term. suppression of *Aedes aegypti* using genetic control does not facilitate *Aedes albopictus*. Pest Manag Sci. 2016; 72(3): 618–628. doi: 10.1002/ps.4151

37. Garziera L, Pedrosa  MC, de Souza FA, Gómez M, Moreira MB, Virginio JF, et al. Effect of interruption of over-flooding releases of transgenic mosquitoes over wild population of *Aedes aegypti*: two case studies in Brazil. Entomol Expt Appl. 2017; 164: 327–339.  https://doi.org/10.1111/eea.12618

## Supporting information

**S1 Fig. Average of weekly larval return rate in control and treatment cages before and after OX513A male adult releases and fluorescence observation. Post OX513A male adult release initiation in treatment cages and following first observation of introgression of OX513A based on fluorescence screening of progeny, the larval return rate was calculated for the treatment cage in proportion to paired control cage. Error bar represents standard error.**

**S2 Fig. Average of total mortality (from week 18) and fluorescence observed (from week 15) in control and treatment cages during suppression period (OX513A male adult releases in treatment cages). Total mortality represents larval/pupal mortality. Error bar represents standard error.**

**S3 Fig. Effect of temperature and humidity on average of weekly egg production in control (A) and treatment cages before and after OX513A release (B & C) during week 1 – 34. *The temperature / humidity represented here is average of maximum / minimum during the week (Wednesday to Tuesday) and average for control and treatment cages.**

**S1 Table. Pupal width measurement (mm) on cephalothoracic region.**

**S2 Table. Measurements of right wing of OX513A and AWD strain *Aedes aegypti* male adults.**

**S3 Table. Reproductivity of adult females recaptured post mating period in field cages.**

**S4 Table. Mating competitiveness between *Aedes aegypti* male adults of transgenic OX513A and wild type AWD strain with wild type AWD strain *Aedes aegypti*.**

**S5 Table. Mean egg count of *Aedes aegypti* AWD strain mosquito in control and treatment cages during the population establishment and stabilization period (week 1 to 14).**

**S6 Table. Average egg count of *Aedes aegypti* AWD strain mosquito in control and treatment cages during the population suppression period, post initiation of OX513A male adult releases in treatment cages from week 15 to 34.**

**S7 Table. Male adult sampling weekly by BG sentinel traps in paired cage unit during pre-release of OX513A in treatment cages.**

**S8 Table. Female adult sampling weekly by BG sentinel traps in paired cage unit during pre-release of OX513A in treatment cages.**

**S9 Table. Correlation between egg laying and temperature and humidity in control and treatment cages (before and after OX513A release).**

Figure 1

Click here to access/download;Figure;Fig1.tif

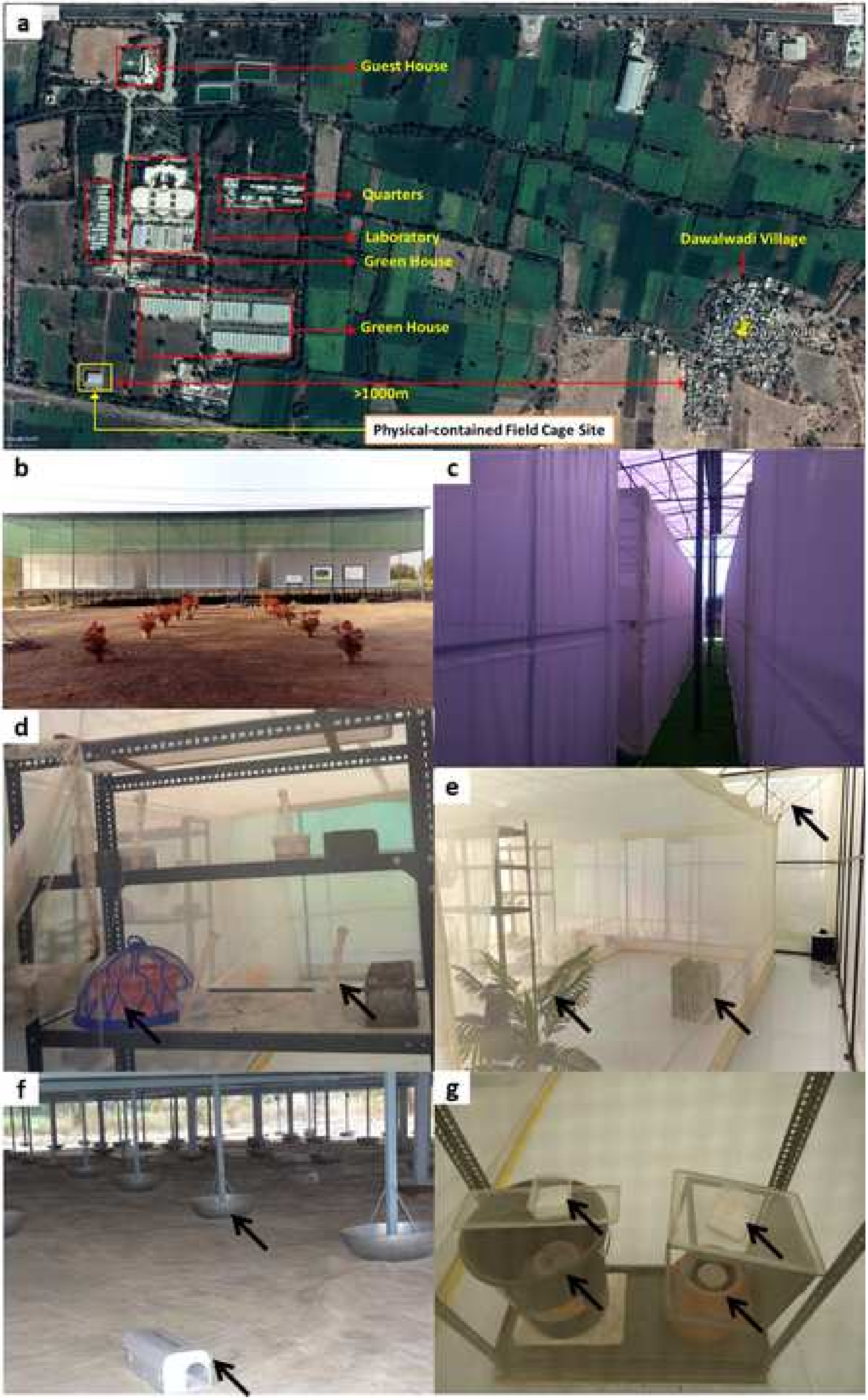

Figure 2

Click here to access/download;Figure;Fig2.tif

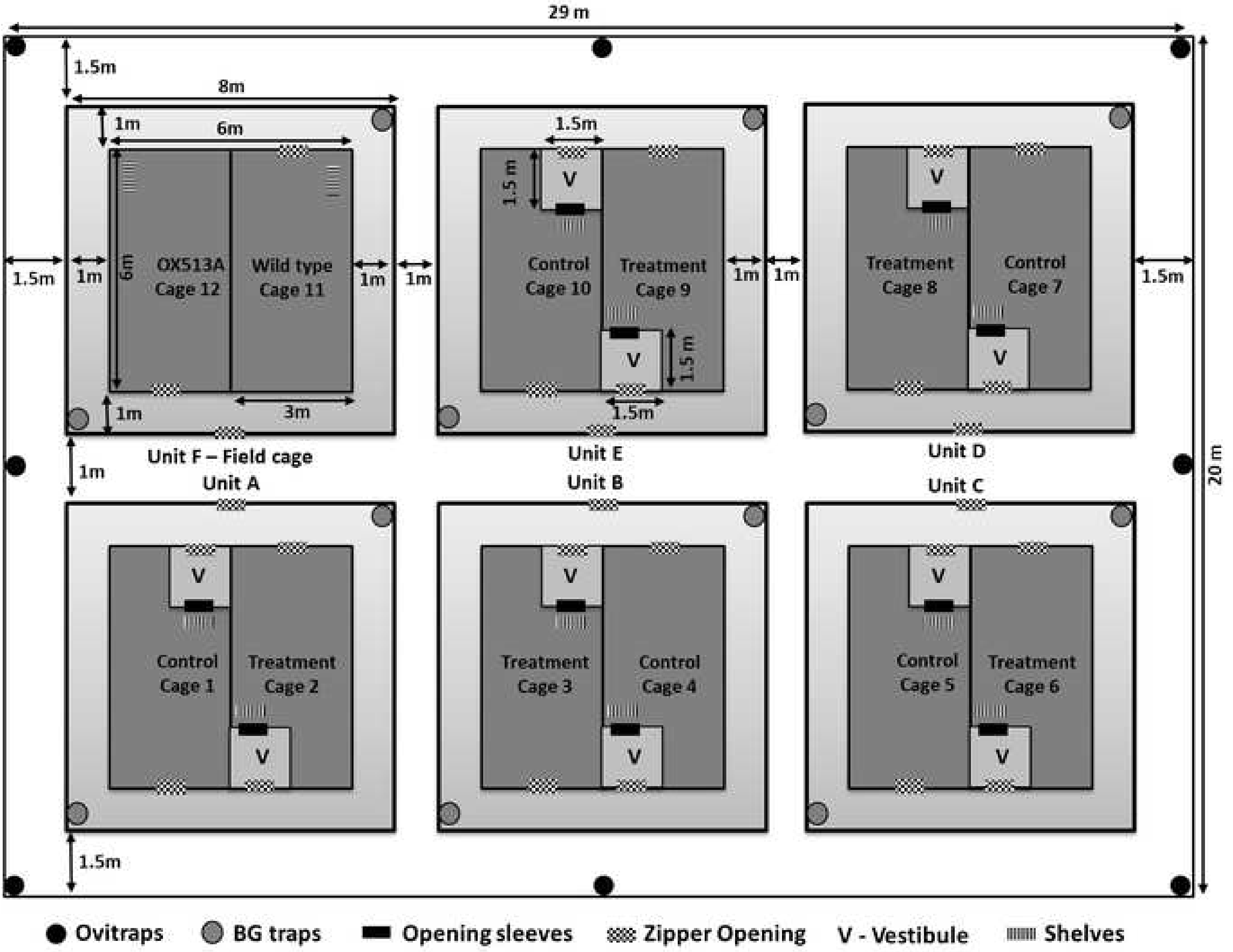

Figure 3

Click here to access/download;Figure;Fig3.tif

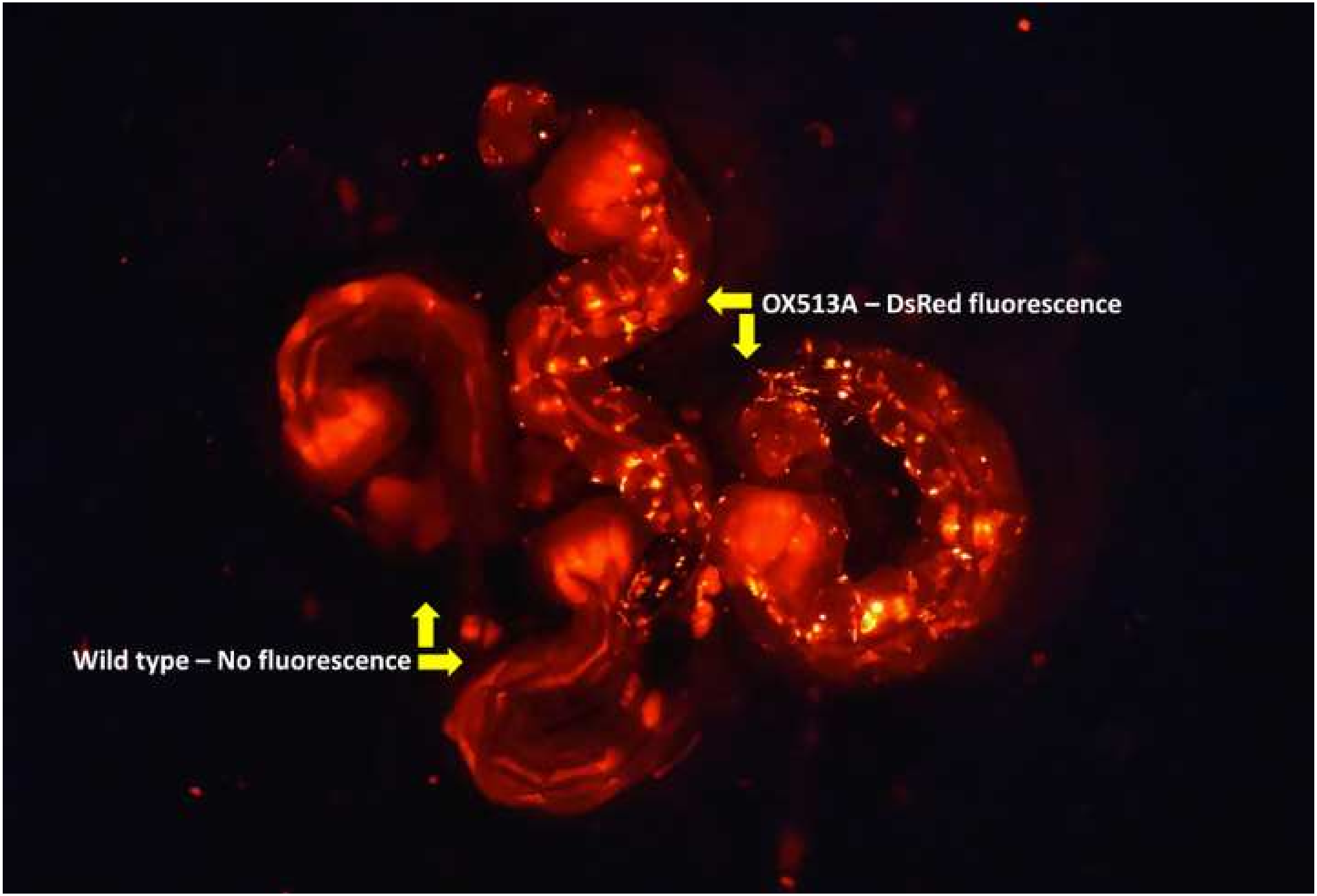

Figure 4

Click here to access/download;Figure;Fig4.tif

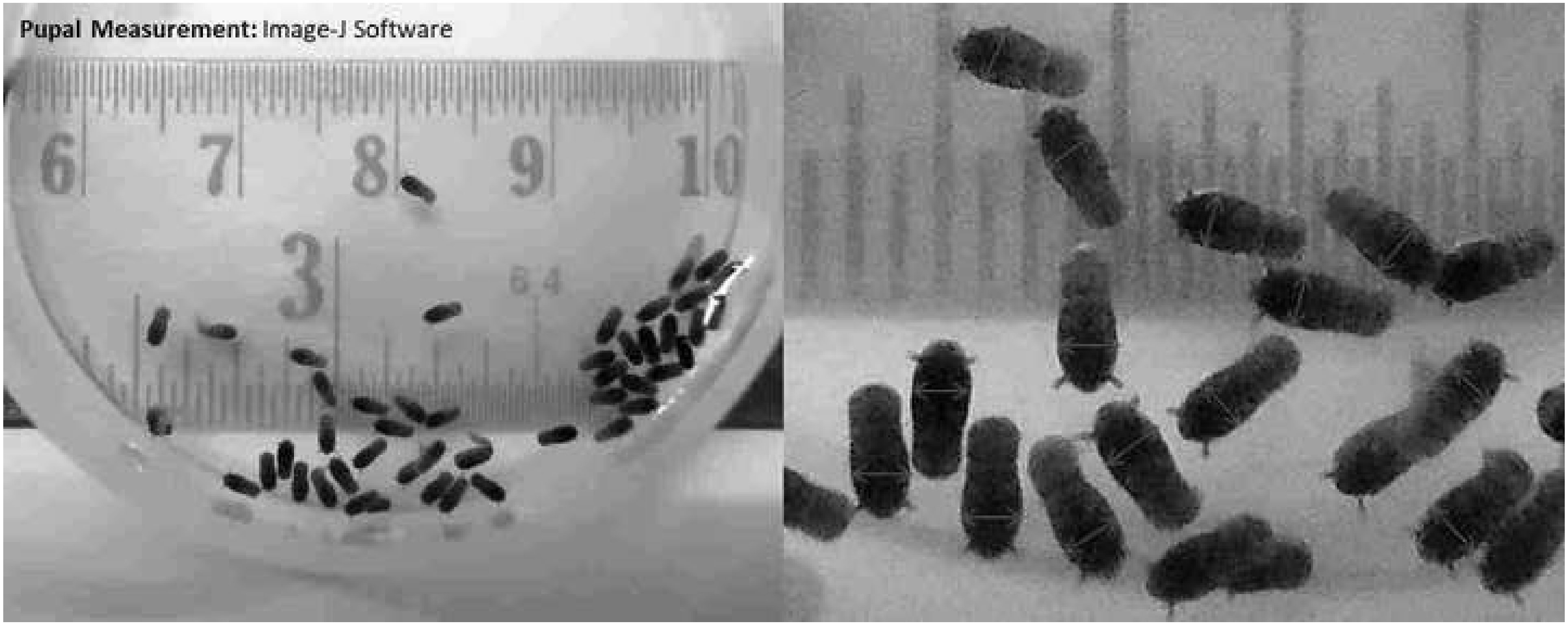

Figure 5

Click here to access/download;Figure;Fig5.tif

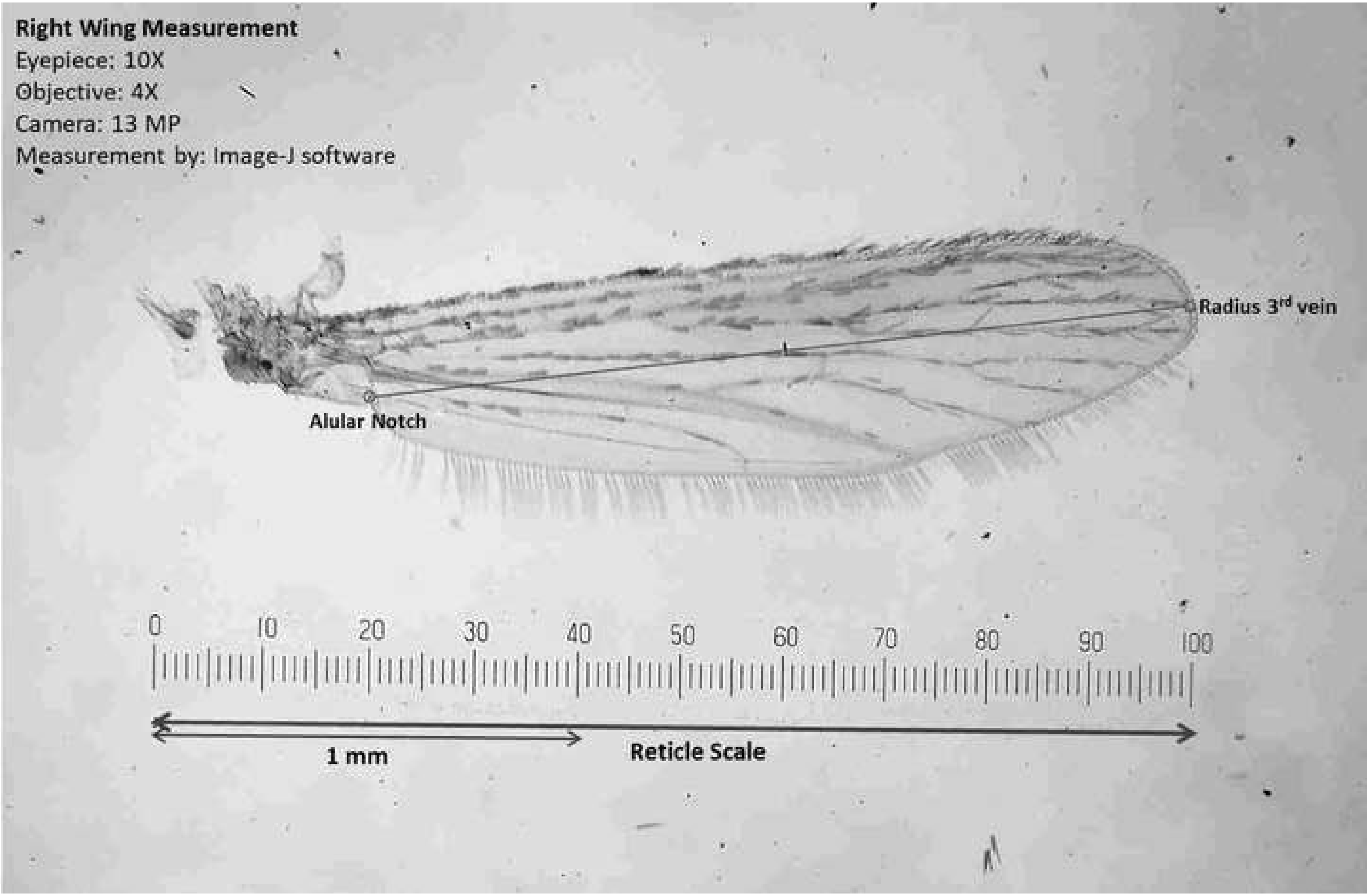

Figure 6

Click here to access/download;Figure;Fig6.tif

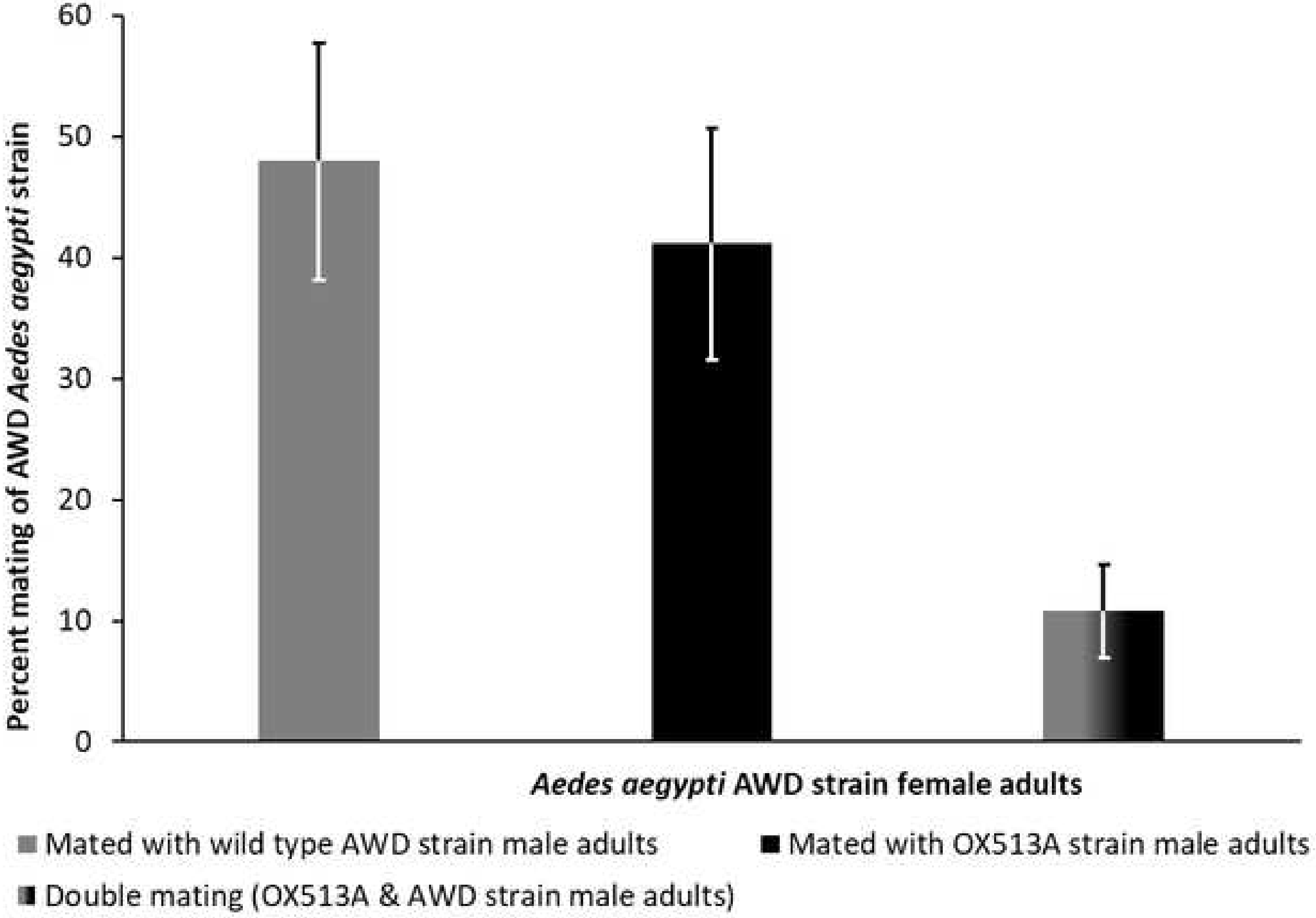

Figure 7

Click here to access/download;Figure;Fig7.tif

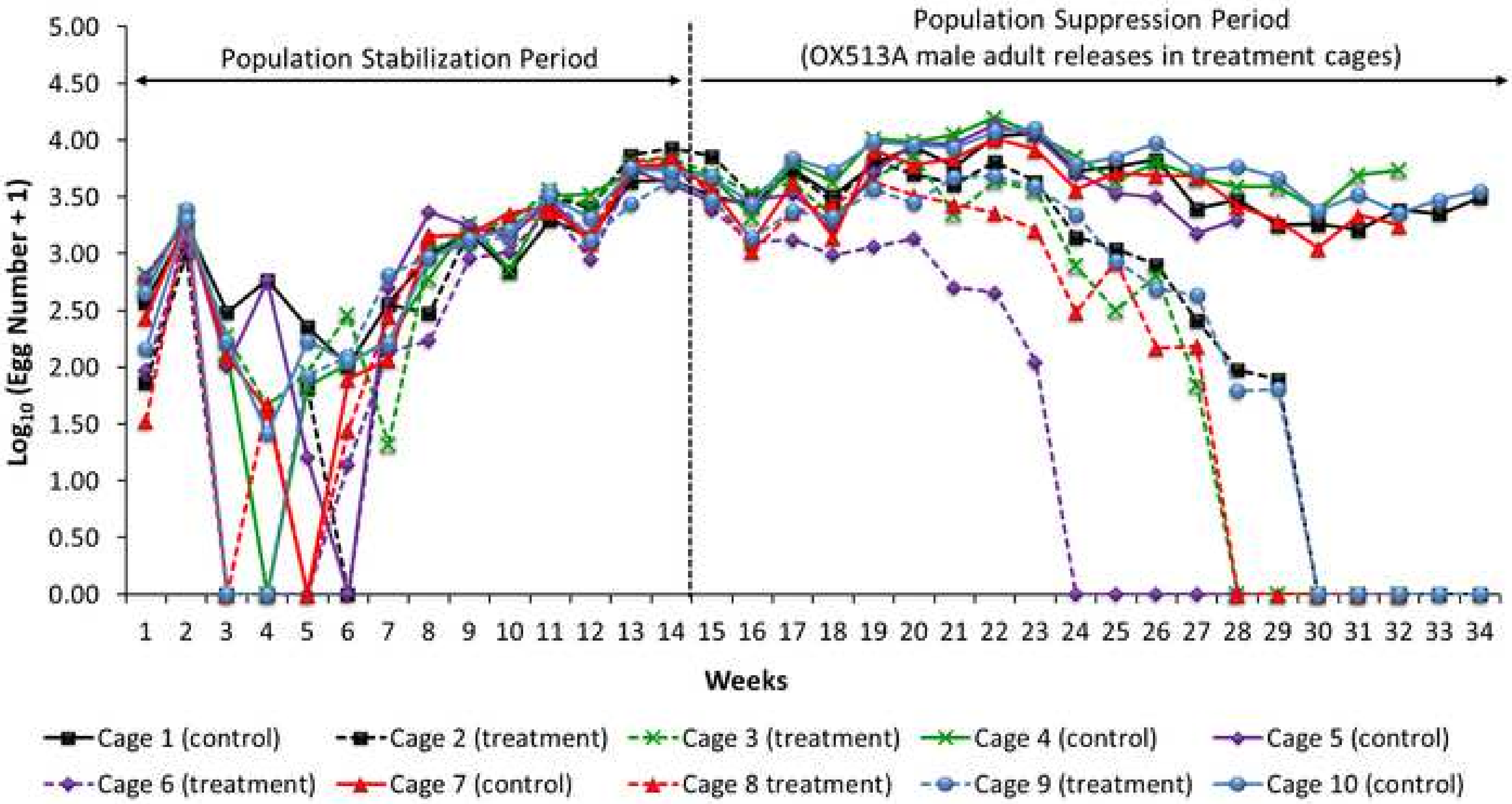

Figure 8

Click here to access/download;Figure;Fig8.tif

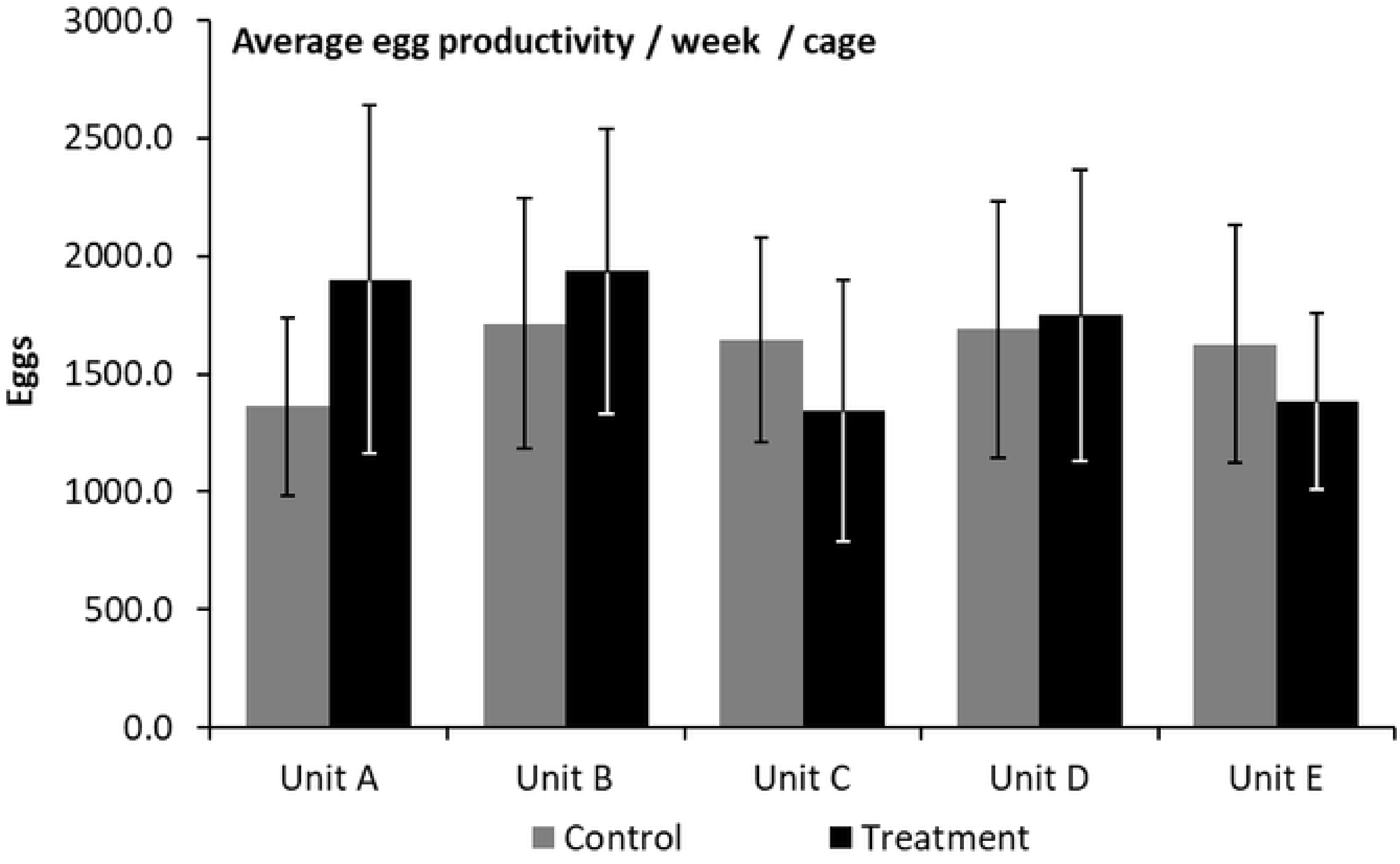

Figure 9

Click here to access/download;Figure;Fig9.tif

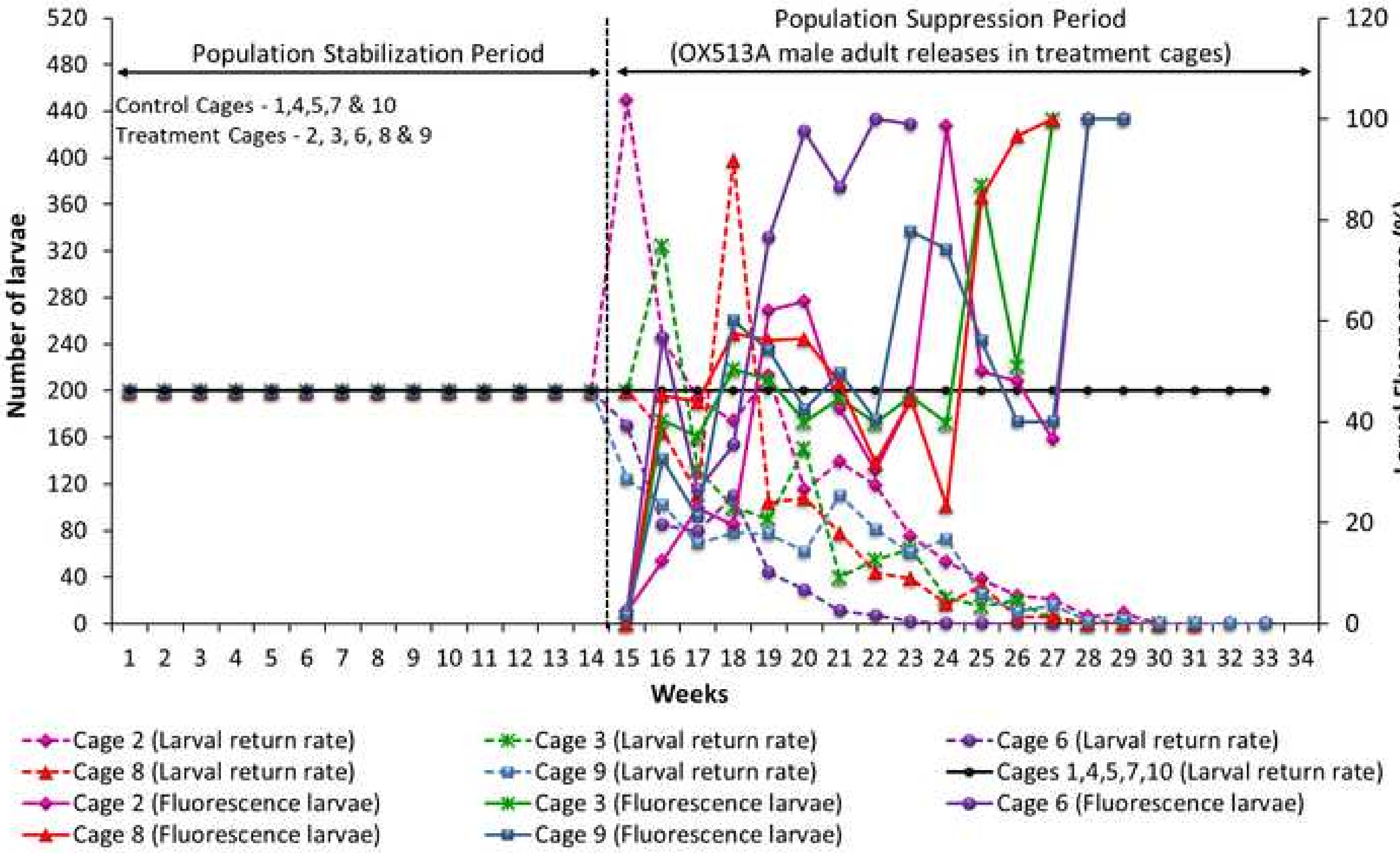

Figure 10

Click here to access/download;Figure;Fig10.tif

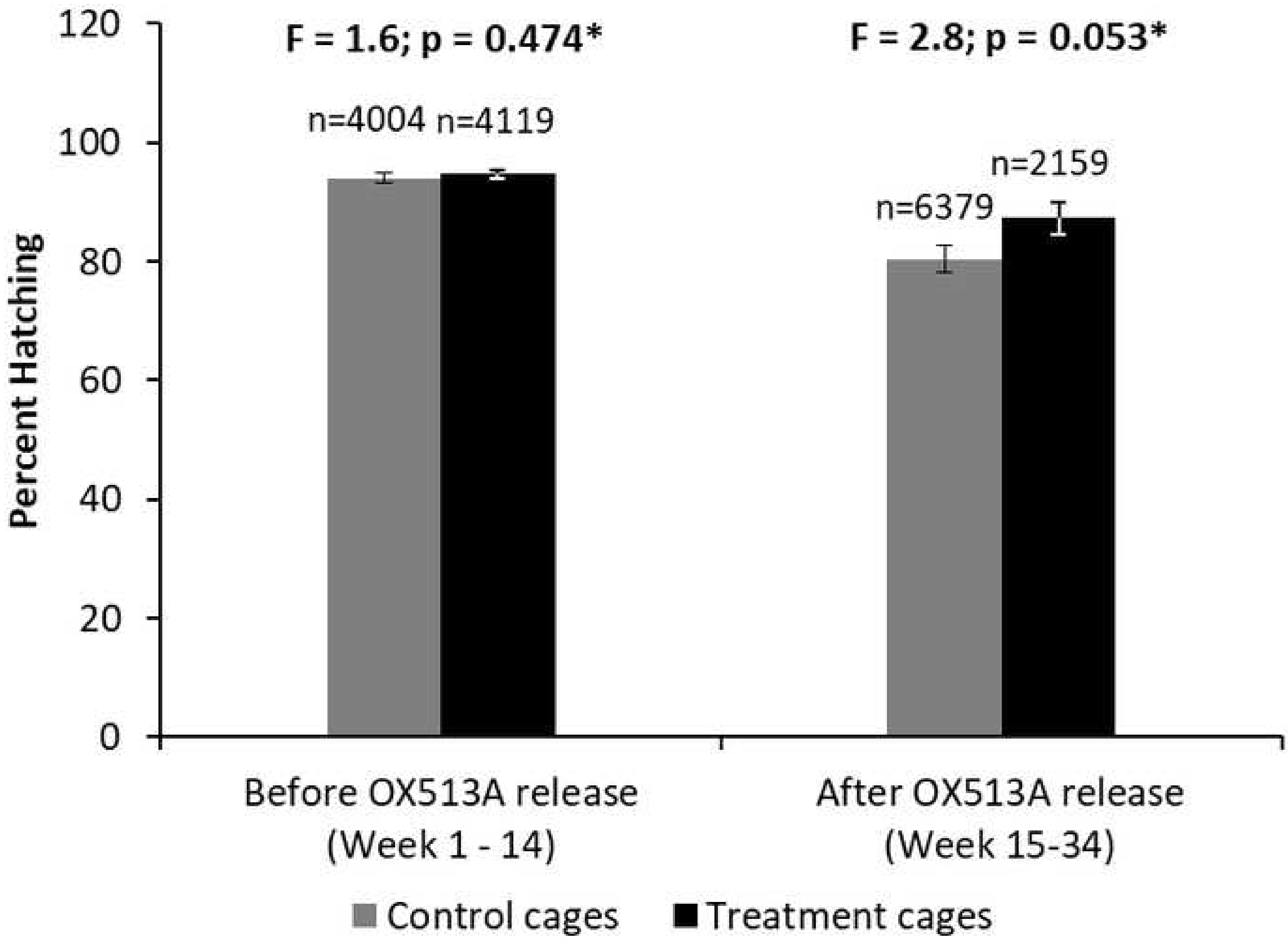

Figure 11

Click here to access/download;Figure;Fig11.tif

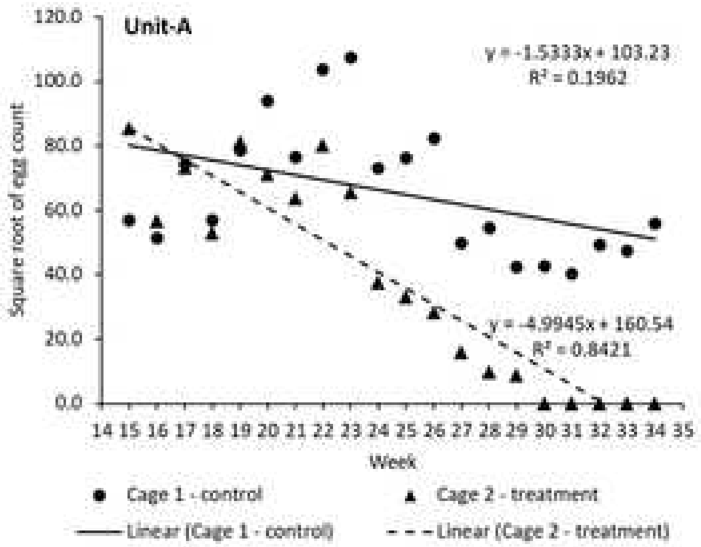


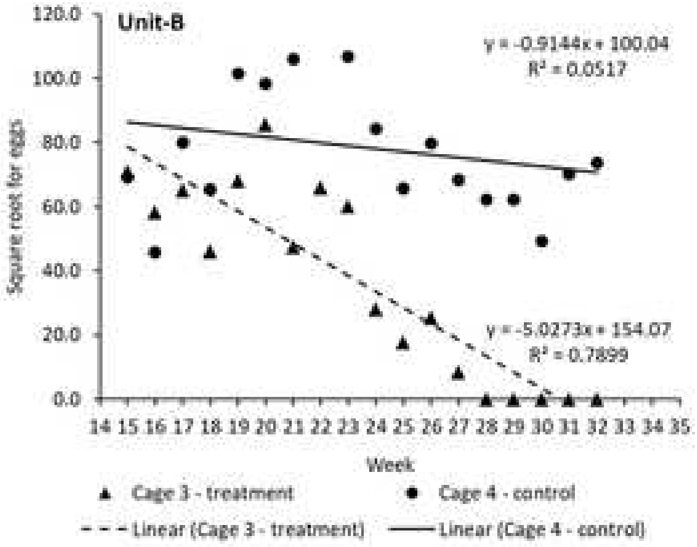


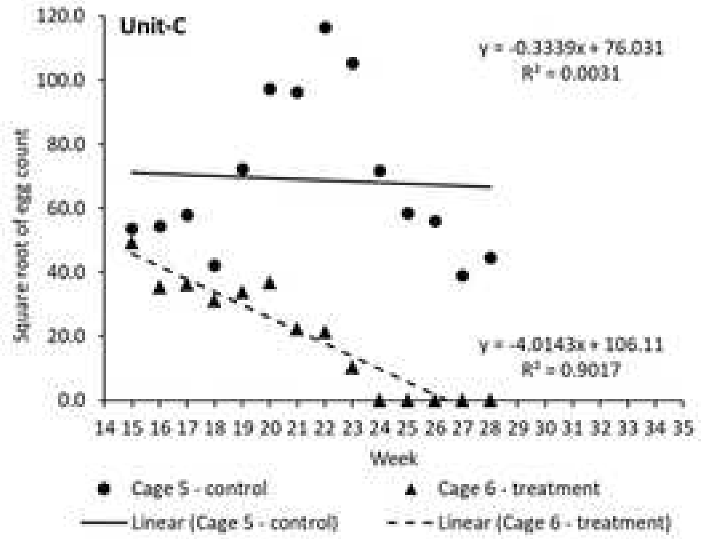


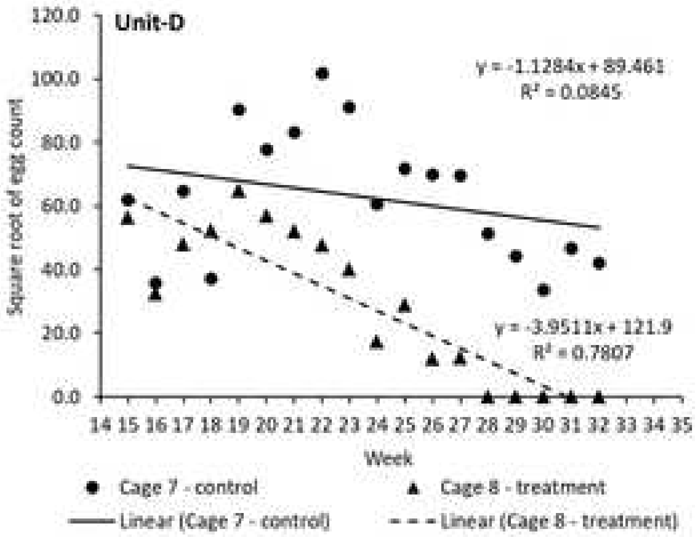


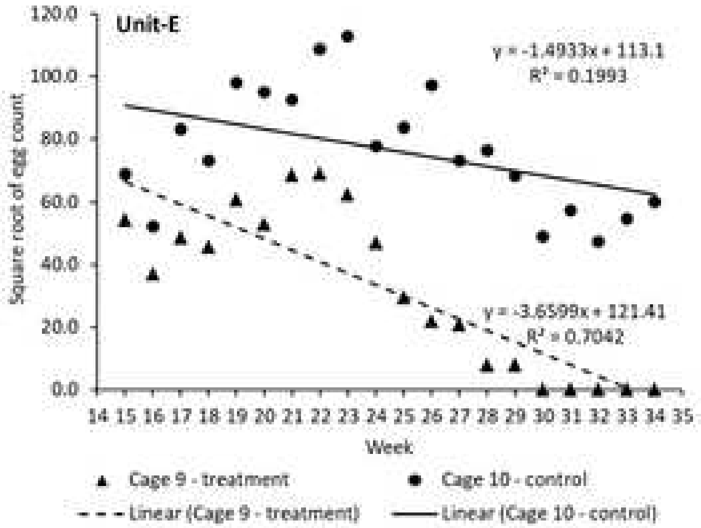


| Paired cage | F value | *p value* |
|---|---|---|
| Unit A | 16.767 | *0.001 |
| Unit B | 7.186 | *0.016 |
| Unit C | 0.157 | 0.699 |
| Unit D | 7.846 | *0.013 |
| Unit E | 24.525 | **<0.001 |

**ANCOVA analysis**

*Difference is significant at <0.05

**Difference is significant at <0.001

Figure 12

Click here to access/download;Figure;Fig12.tif

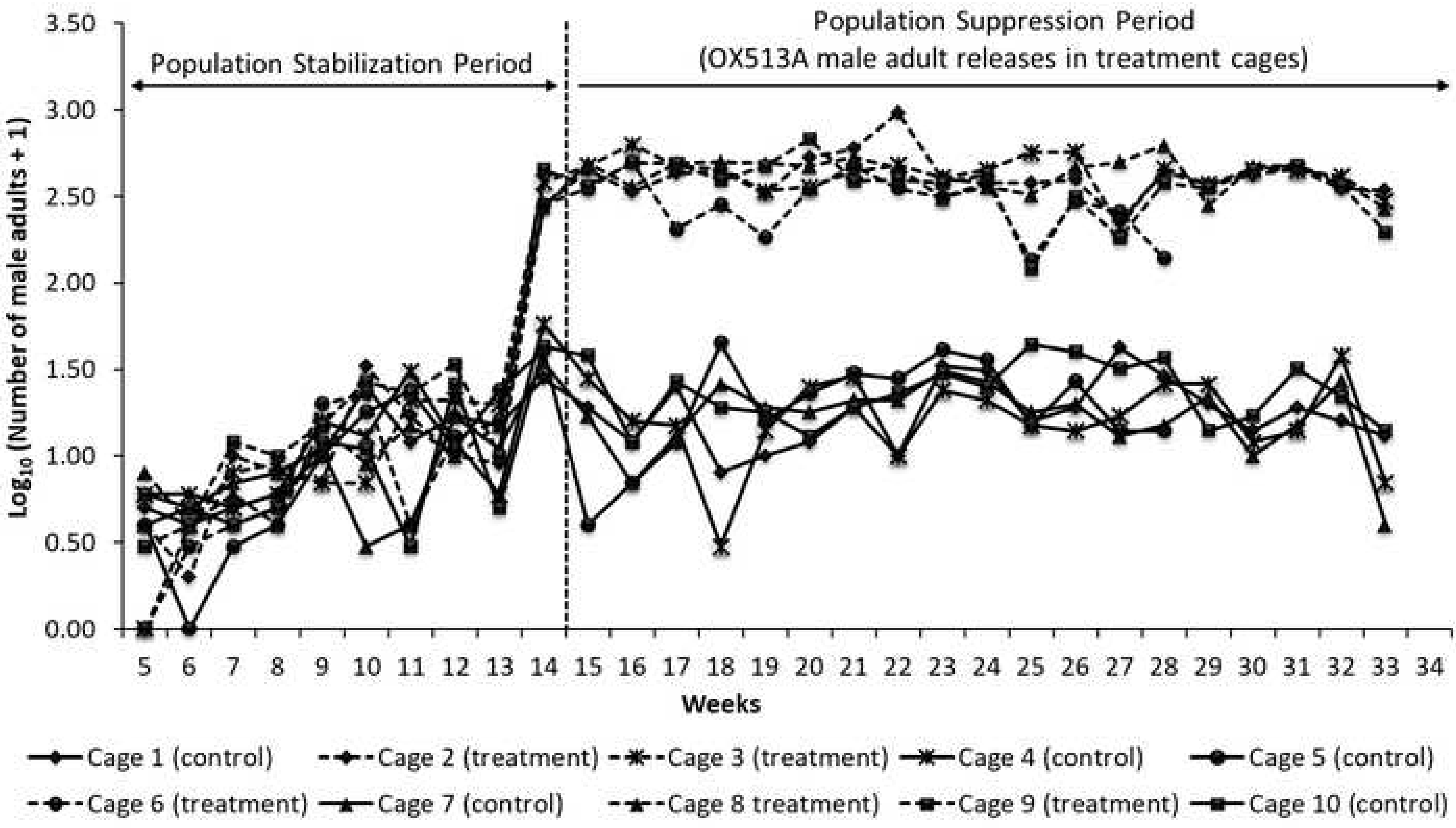

Figure 13

Click here to access/download;Figure;Fig13.tif

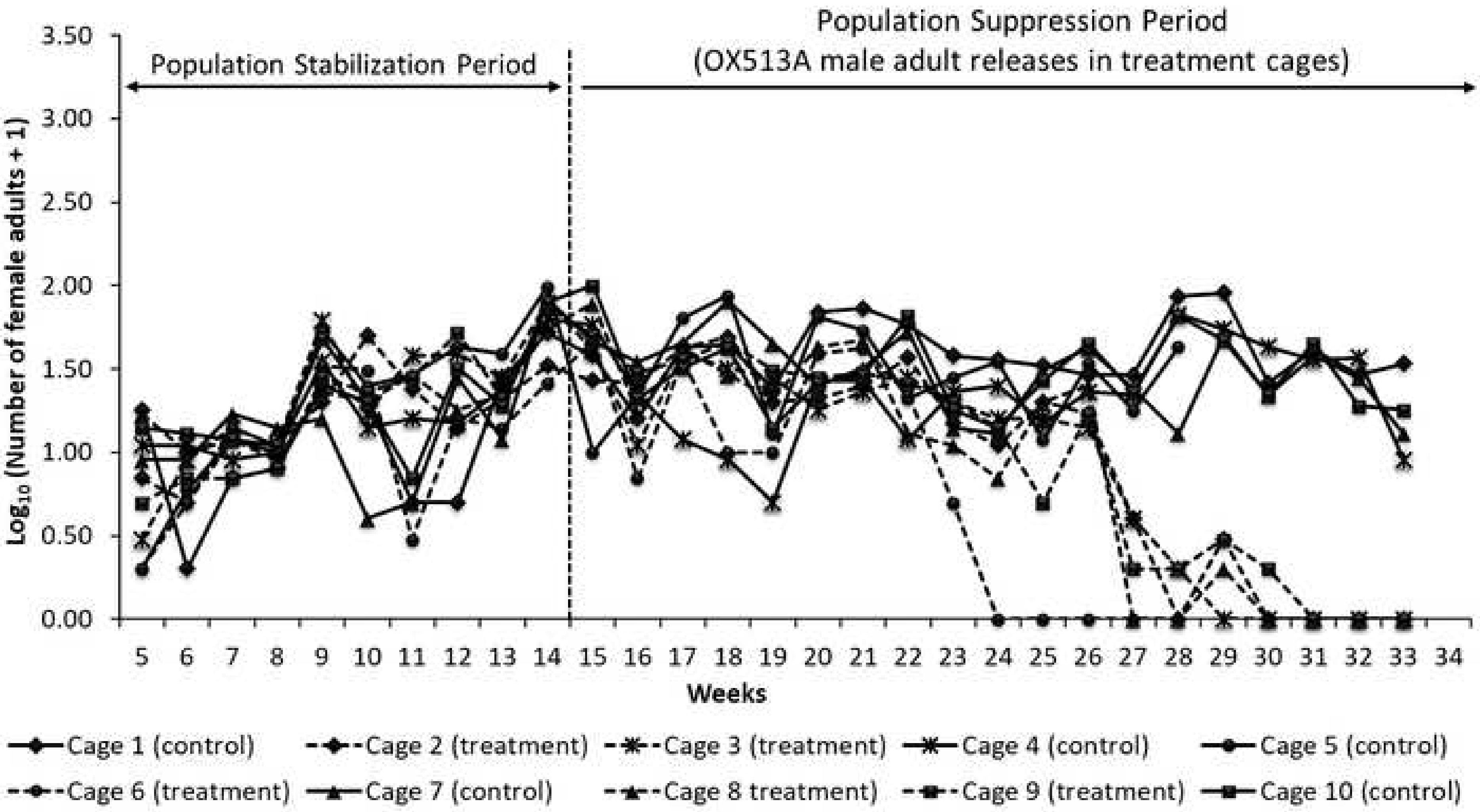

S1 Table

Click here to access/download
**Supporting Information**
S1_table.pdf.pdf

S2 Table

Click here to access/download
**Supporting Information**
S2_table.pdf.pdf

S3 Table

Click here to access/download
**Supporting Information**
S3_table.pdf.pdf

S4 Table

Click here to access/download
**Supporting Information**
S4_table.pdf.pdf

S5 Table

Click here to access/download
**Supporting Information**
S5_table.pdf.pdf

S6 Table

Click here to access/download
**Supporting Information**
S6_table.pdf.pdf

S7 Table

Click here to access/download
**Supporting Information**
S7_table.pdf.pdf

S8 Table

Click here to access/download
**Supporting Information**
S8_table.pdf.pdf

S9 Table

Click here to access/download
**Supporting Information**
S9_table.pdf.pdf

S1 Figure

Click here to access/download
**Supporting Information**
S1_fig.tif.tif

S2 Figure

Click here to access/download
**Supporting Information**
S2_fig_tif.tif

S3 Figure

Click here to access/download
**Supporting Information**
S3_fig.tif.pdf
