# Supplementary material for: Elimination of a closed population of the yellow fever mosquito, Aedes aegypti, through releases of self-limiting male mosquitoes
Source: PLoS Negl Trop Dis. 2022 May 16;16(5):e0010315. doi: 10.1371/journal.pntd.0010315 (PMC9135344; doi:10.1371/journal.pntd.0010315)
Supplement: Attachment — Submitted filename: Responses to Reviewers.pdf [file pntd.0010315.s026.pdf]

Responses to Reviewers Comments

Title of the manuscript: “Elimination of a closed population of the yellow fever mosquito, *Aedes aegypti*, through releases of self-limiting male mosquitoes”.

REVIEWER #1

Response: *All the marked-up comments in the PDF file have been addressed and responded and the manuscript has been revised as per the comments / suggestions.*

REVIEWER #2

Comment: Clarify the identity of the transgenic strain used, which is essentially obsolete.

This was the original “1st generation Friendly “strain, OX513A. This can now be considered obsolete, replaced by the “2nd generation strain, OX5034".

The difference is important because whereas the original strain was lethal to all the offspring of females mated with the transgenic strain, the new strain is only lethal to the females; half of the male offspring carry the gene and survive to pass it onto the next generation.

I do not suggest that the study is obsolete but suggest that this difference should be made clear.

Response*: A paragraph has been added in the discussion section (at the end) on the transition from the first generation technology to second generation technology has been added to clarify on the identity.*

****
