# Supplementary material for: Elimination of a closed population of the yellow fever mosquito, Aedes aegypti, through releases of self-limiting male mosquitoes
Source: PLoS Negl Trop Dis. 2022 May 16;16(5):e0010315. doi: 10.1371/journal.pntd.0010315 (PMC9135344; doi:10.1371/journal.pntd.0010315)
Supplement: Attachment — Submitted filename: Review of eradication Aedes aegypti 1st revision.pdf [file pntd.0010315.s028.pdf]

Review of first revision of: Eradication of a closed population of the yellow fever mosquito, *Aedes aegypti*, through releases of self-limiting male mosquitoes

By Patil et al.

The paper has been extensively revised and is much better organized and very readable. I have put sever comments in the marked-up PDF regarding mainly style issues.

The studies conducted here are part of the phases for testing genetically modified mosquitoes released by WHO, and they provide confidence that what was observed elsewhere is likely to be observed in India.

My comments about the organization of the paper have been taken into consideration and it is much improved.

I have only one further over-arching comment. There are too many figures. Some could be combined (e.g. 12 and 13) into one figure. Others could be moved to the supplemental information. For an average reader, they will be most interested in 'the money figures' which consist of solid demonstrations of population suppression. Projects like this create too much data to present it all.
