# Supplementary material for: Elimination of a closed population of the yellow fever mosquito, Aedes aegypti, through releases of self-limiting male mosquitoes
Source: PLoS Negl Trop Dis. 2022 May 16;16(5):e0010315. doi: 10.1371/journal.pntd.0010315 (PMC9135344; doi:10.1371/journal.pntd.0010315)
Supplement: Attachment — Submitted filename: PNTD OX513A.docx [file pntd.0010315.s022.docx]

The manuscript describes a trial of the efficacy of transgenic male Aedes aegypti in suppressing a cage population of a strain (OX513A) derived from eggs collected in a small village close to Jalna, a city in Maharashtra State in India.

Despite an immense amount of detailed description, extensive data and exhaustive statistical analysis I regret to say that I see little difference between this study and other cage studies using the same transgenic strain that have been published in the past 15 years. The only difference is that a local strain was used.

One piece of information, however, intrigues me: 10.9% of females of the local strain mated with both local males and OX513A. Although there are reports of a second mating a few hours after the first, it is generally assumed that the species is monogamous.

In my opinion, the manuscript should only be accepted if whittled down to a short report presented as confirmatory to previous studies. On the other hand, I suggest that further exploration of the question of polygamy could be interesting.

(Details of the OX513A transgenic strain can be accessed at: <https://www.oxitec.com/en/public-health>)
